# A high-efficiency bioinspired photoelectric-electromechanical integrated nanogenerator

**DOI:** 10.1038/s41467-020-19987-0

**Published:** 2020-12-02

**Authors:** Sicheng Liu, Xi Liu, Guilin Zhou, Fuxiang Qin, Mingxing Jing, Lin Li, Wenlong Song, Zhuangzhi Sun

**Affiliations:** 1 Province Key Laboratory of Forestry Intelligent Equipment Engineering, College of Mechanical and Electrical Engineering, Northeast Forestry University, 150000 Harbin, People’s Republic of China; 2 Key Laboratory of Bio-based Material Science & Technology, Ministry of Education, Northeast Forestry University, 150000 Harbin, People’s Republic of China; 3 Key Laboratory for Photonic and Electronic Bandgap Materials, Ministry of Education, School of Physics and Electronic Engineering, Harbin Normal University, 150000 Harbin, People’s Republic of China

**Keywords:** Devices for energy harvesting, Mechanical engineering

## Abstract

Currently, the key challenge in triboelectric nanogenerators (TENGs) is how to efficiently enhance the surface charge density. Here, a new strategy is proposed to increase the surface charge density by comprehensively utilizing solar energy and tidal energy, and a bioinspired photoelectric-electromechanical integrated TENG (Pem-iTENG) is developed. This enhancement of output performance is greatly attributed to the accumulation of photoelectrons from photocatalysis and the triboelectric negative charges from contact electrification. Pem-iTENG shows a maximal open-circuit voltage of 124.2 V and a maximal short-circuit current density of 221.6 μA cm^−2^ under tidal wave and sunlight, an improvement by nearly a factor of 10 over that of reported TENGs based on solid-liquid contact electrification. More importantly, it exhibits a high energy conversion efficiency according to the evaluation method for solar cells. This work provides insights into development of high-performance TENGs by using different natural energy sources.

## Abstract (web summary)

Increasing the surface charge density of a triboelectric nanogenerator (TENG) under a single energy source is of wide interest. Here, the authors increase the surface charge density by comprehensively utilizing solar energy and tidal energy to develop a bioinspired photoelectric-electromechanical integrated TENG.

## Introduction

Recently, with the increasing shortage of global energy, technologies for energy collection from the surrounding environment, such as geothermal energy^1,2^, ocean energy^3,4^, solar energy^5–7^, electromagnetic energy^8,9^ and chemical energy^10^, have attracted worldwide attention. The triboelectric nanogenerator (TENG)^11^, first proposed in 2012 by coupling the physical effects of contact electrification and electrostatic induction^12,13^, can harvest irregular mechanical motions, offering great potential to alleviate the energy crisis. However, TENGs induced by vertical contact-separation mode exhibit certain disadvantages in harvesting tidal energy, and its electrification effect is significantly weakened in contrast to their use in energy harvesting from human motion and wind^14^. Hence, the energy conversion efficiency of this energy system needs to be further explored. In fact, TENGs are repeatedly submerged during the interaction of water and insulating polymers^15–17^, and their power generation depends strongly on the surface charge density. The principle of the superposition of photocatalysis and contact electrification to increase the surface charge density is inspired^18^. Therefore, certain limitations are still encountered in driving TENGs under a single energy source, such as mechanical energy (especially in solid-liquid contact, vertical contact-separation) or solar energy^19–21^. The comprehensive utilization of multiple natural energies and the realization of efficient energy conversion efficiency are the current challenges in power generation.

Because the micro/nanostructure (2–5 μm) of the mastoid cilia on the lotus leaf has advantages such as self-cleaning and super-hydrophobic, we introduce this flexible rod-like structure onto Pem-iTENG surface in a reciprocating scouring process. Meanwhile, polyaniline (PANI) as a P-type conductive polymer is well matched with titanium dioxide (TiO_2_) as a N-type semiconductor to construct a P-N heterojunction. Similar to the photoreaction stage of photosynthesis in green plants^22–24^, this heterojunction can suppress the recombination of photogenerated electron-hole pairs and improve the quantum efficiency of photocatalytic reactions^25,26^. Hence, it creates the possibility that the photoelectrons or holes in photocatalysis can be retained to increase the surface charge density.

Here, we propose a new strategy to enhance the surface charge density by comprehensively utilizing solar energy and tidal energy, and develop a high-efficiency bioinspired photoelectric-electromechanical integrated triboelectric nanogenerator (Pem-iTENG) based on solid-liquid contact electrification. Topological super- hydrophobic bionic cilia using polydimethylsiloxane (PDMS)/cobalt (Co) powder are self-grown on the surface of PDMS by a template-free method, and a type-II P-N heterojunction (TiO_2_/PANI) is planted on the surface of bionic cilia to construct composite membrane (Pem-iCM) by a layer-by-layer self-assembly method. Pem-iTENG shows a maximal open-circuit voltage of 124.2 V and a maximal short-circuit current density of 221.6 μA cm^−2^ under the action of tidal waves and sunlight. It also exhibits high energy conversion efficiency referring to the evaluation method for solar cells. This work demonstrates a photoelectric-electromechanical integrated energy conversion technology, and proposes insights into the development of high-performance TENGs by using different natural energy sources.

## Results

### The working principle of Pem-iTENG

**Fig. 1:**
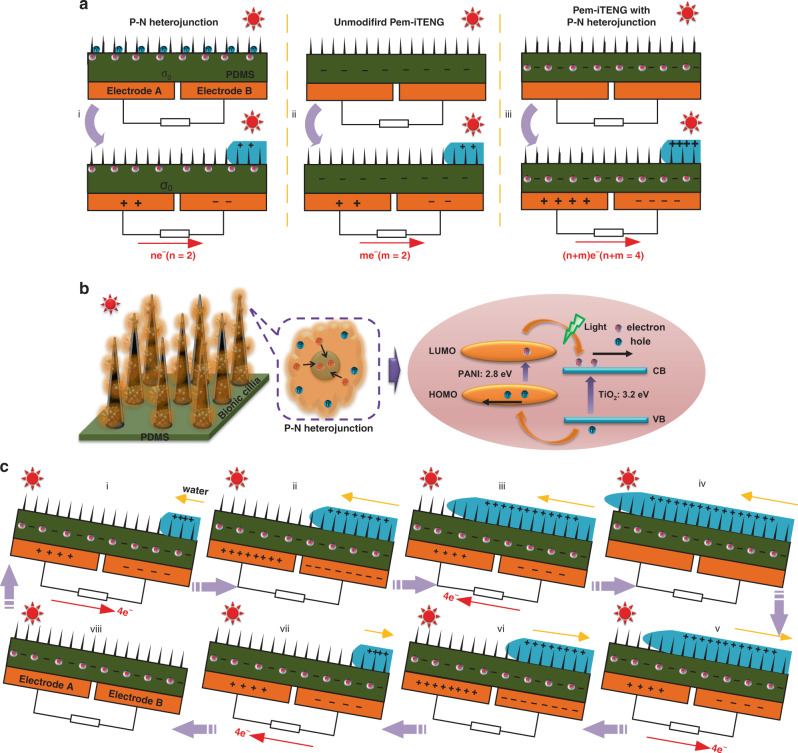
The working principles of Pem-iTENG. **a** Pem-iTENG with P-N heterojunction (i). Unmodified and modified Pem-iTENG during the two flow states of the tidal waves (ii and iii). **b** Principles of photocatalysis of bionic cilia with a P-N heterojunction. **c** The contact electrification of Pem-iTENG in a cycle of reciprocating tidal waves and free electron transfer direction between the two electrodes.

The designed Pem-iTENG has a surface topology consisting of bionic cilia covered with TiO_2_/PANI (Supplementary Figs. 1, 2 and Supplementary Note 1, 2). Our Pem-iTENG accumulates numerous negative charges in the PDMS film due to the excess photogenerated electrons (also called photoelectrons) from photocatalysis and the triboelectric negative charges from contact electrification. Here, the transfer electrons in the contact electrification induced by photoelectrons (Fig. 1(a)i), triboelectric negative charges (Fig. 1(a)ii), and the superposition of two charges (Fig. 1(a)iii) are described separately to explain this enhancement effect. The transfer electron represents the difference per unit area between the latter flow state of the tidal wave (e.g., in Fig. 1(c)i) and the former flow state of the tidal wave (e.g., in Fig. 1(c)viii). We assume that there is no triboelectric negative charge in Fig. 1(a)i, only photoelectrons, and that the transfer electron is *n* under tidal waves and sunlight. The transfer electron unmodified TiO_2_/PANI, only triboelectric negative charge, is *m* under tidal waves and sunlight (Fig. 1(a)ii). Therefore, the transfer electrons of Pem-iTENG are *m* + *n* by superposition photoelectrons and triboelectric negative charge under tidal waves and sunlight (Fig. 1(a)iii).

The working principle of our Pem-iTENG can be described as follows. A type II P-N heterojunction is planted on the surface of bionic cilia. When it is illuminated by the ultraviolet (UV) and visible portions of incident sunlight, the effective separation of the photogenerated electrons and holes (Fig. 1b) can be realized under the built-in field of the P-N heterojunction^27–29^. Excess photoelectrons are retained in TiO_2_ because the TiO_2_ particles are enclosed by PANI and bionic cilia. The photogenerated holes are transferred to PANI, where they contact and react with moist sea water mist or organic impurities. This enhances the surface negative charge density of the PDMS film. Subsequently, according to solid-liquid contact electrification^19,30–34^, we propose the working principle of Pem-iTENG under the synergy of contact electrification and photocatalysis in a cycle of reciprocating tidal waves (see Fig. 1c).

Once water waves immerse the surface of the PDMS film with bionic cilia, the contact sea water forms an electric double layer with the PDMS film that partly screens the negative charges on the PDMS film. Due to the asymmetric distribution of charges, the unbalanced potential between the two electrodes drives free electrons to flow from one electrode to the other and forms an alternating current through the external circuit (see Fig. 1(c)i, iii, v, vii). When the tidal wave rises or falls to the gap between the two electrodes, there is no transfer electron (Fig. 1(c)ii, vi). When the tidal wave rises to the top (Fig. 1(c)iv), the potential difference between the two electrodes decays to zero without transfer electrons due to the symmetrical screening of the electric double layer. During the first rise of the water wave, the PDMS film continues to accumulate triboelectric negative charges and photoelectrons without dissipating for a period of time, and there is no transfer electron between the two electrodes (Fig. 1(c)viii). It is worth noting that the sea water remains electrically neutral throughout the entire contact electrification.

### Morphology characterization of bioinspired Pem-iCM

**Fig. 2:**
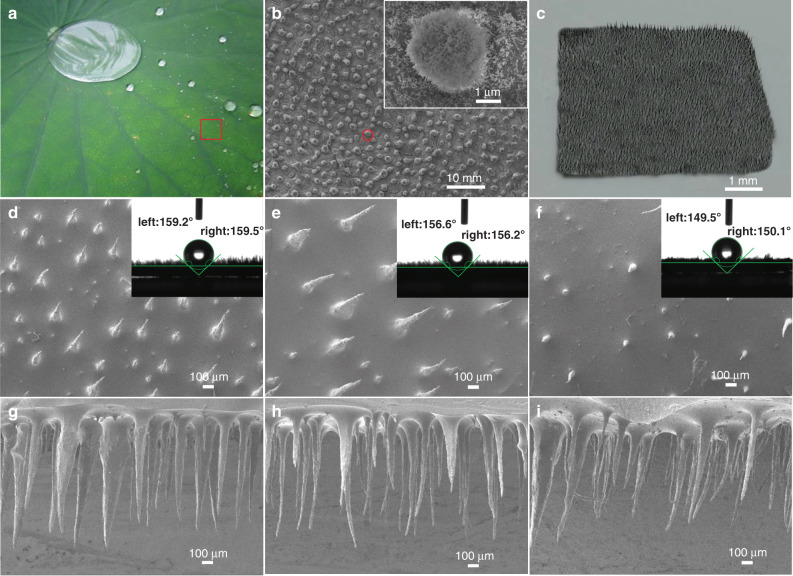
Morphological characterization of lotus leaf and bionic cilia. **a** Prototype of lotus leaf. **b** SEM image of lotus leaf. The inset shows a papillary protrusion on the surface of lotus leaf. **c** The prepared physical sample of Pem-iCM. **d**–**f** Different aspect ratios of bionic cilia (Supplementary Table 1, aspect ratio (47, 25, 13) = Length (mm)/Diameter (mm)). The insets show the water contact angles of three samples. **g**–**i** Cross-sectional SEM images of bionic cilia with different aspect ratios.

The surface of a lotus leaf is covered with a large number of micromastoid and wax materials, and each micromastoid is covered with a large number of nanoscale branchlike super-hydrophobic cilia (Fig. 2a). When the water droplets contact the lotus leaf surface, the effective flow of the water droplets will occur (Fig. 2b). According to Cassie and Wenzel wetting theory, the horizontal reciprocating motion will increase air pocket contact in the surface, and the droplets will leave minimal residue on the cilia surface during reciprocating scouring. Inspired by it, magnetic growth of bionic cilia induced by cobalt powder (sizes of 300 nm, 800 nm, 1300 nm) strategy is proposed. The experimental results show the size of Co powder affects the density and length of the bionic cilia (Fig. 2c). As the size of Co particle increases, the density of bionic cilia gradually decreases (Supplementary Table 1). This is because the larger the particle size is, the greater the gravitational force, leading to the poor dispersibility in mixture solution with PDMS/Co.

Moreover, as the size of the Co nanoparticle increases, the contact angle of bionic cilia decreases gradually. The contact angle of Pem-iCM with a size of Co nanoparticle of 300 nm is the largest in the three samples (>150°), which verifies its surface super-hydrophobic property (Fig. 2d–f). As the size of the Co nanoparticle increases, the length of bionic cilia decreases gradually (Fig. 2g–i), and the roughness of a single cilium is the largest (Fig. 2i). When bionic cilia are modified by PANI/TiO_2_, the surface roughness of Pem-iCM is not changed, and its influence on electrical properties of Pem-iTENG during the reciprocating scouring can be ignored (Supplementary Figs. 3, 5). The surface of Pem-iCM is similar to the conical structure of lotus leaf with many finer fibers bundled together. The diameter of bionic cilia is about 10–80 μm, and the length of bionic cilia is ~1 mm.

### Electrical characteristics and photoelectric mechanism of Pem-iTENG

**Fig. 3:**
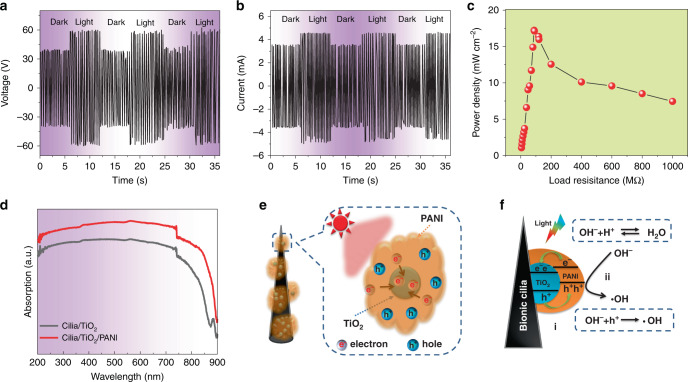
Electrical performance and photoelectric mechanism of Pem-iTENG. **a** The curve of open-circuit voltage under dark/light cycle. **b** The curve of short-circuit current under dark/light cycle. **c** Dependence of output power density of Pem-iTENG on the resistance of external load under light irradiation. **d** Absorption spectrum of Pem-iCM. **e** Schematic illustration of electron-hole separation and transport between TiO_2_ and PANI. **f** Schematic illustration of charge transfer and photocatalysis reaction.

The open voltage (Fig. 3a) and the short-circuit current (Fig. 3b) of Pem-iTENG are measured under periodic motion (2 Hz) under dark/light cycle. It can be seen that the open-circuit voltage increases from 38.8 V (dark) to 59.87 V (light), a factor of 1.54. The open-circuit voltage recovered quickly to ~38 V when the light is turned off. Figure 3b shows that the output current increases rapidly from dark to light, and the positive short-circuit current increases from 2.82 mA (dark) to 4.55 mA (light), a factor of 1.67. The negative short-circuit current increases from −3.55 mA (dark) to −4.97 mA (light), a factor of 1.40.

The output power density of Pem-iTENG first increases and then decreased as the load resistance increases under light irradiation (Eq. (1) and Fig. 3c). When the load resistance is 88 MΩ, the maximal output power density is 17.23 mW cm^−2^ under the light. It indicates that our Pem-iTENG with this external resistance can obtain the maximal output power density under illumination of light.

Figure 3d shows the absorption spectra of Pem-iCM modified with and without PANI. It can be seen that the Pem-iCM modified with PANI shows an enhance absorption characteristic over the entire measurement range (200–900 nm) due to the good absorption property of PANI. It is very helpful for Pem-iCM to absorb sunlight.

The photoelectric working principle of Pem-iTENG is revealed in Fig. 3e, f. When TiO_2_ on bionic cilia is covered with PANI, a type-II P-N heterojunction is formed at the surface of bionic cilia. When it is illuminated by sunlight, TiO_2_ and PANI primarily absorb the UV and visible portions of the incident light, respectively. The photogenerated electrons and holes are separated by the built-in field of the P-N heterojunction^27–29^, and they move into TiO_2_ (photoelectrons) and PANI (holes) (Fig. 3e). When Pem-iTENG is placed in a marine environment, the PANI covered at the surface of bionic cilia is exposed to moist water mist, organic matter, etc. The holes (h^+^) at the surface of PANI react with water molecules to form hydroxyl radicals (Fig. 3(f)ii), which contribute to the oxidation and degradation of organic molecules (Supplementary Fig. 4), while excess photoelectrons will remain in TiO_2_ because the TiO_2_ particles are enclosed between PANI and bionic cilia (see Fig. 3(f)i). Hence, the surface negative charge density of the PDMS film will increase. Subsequently, as the tidal waves move, solid-liquid contact between sea water and the PDMS film induces the contact electrification^19,32,35,36^. In addition, our experiments confirmed that the increase of surface charge density of Pem-iTENG is derived from photocatalysis (Supplementary Figs. 5, 6, and Supplementary Table 2).

### Design and structure of Pem-iTENG

**Fig. 4:**
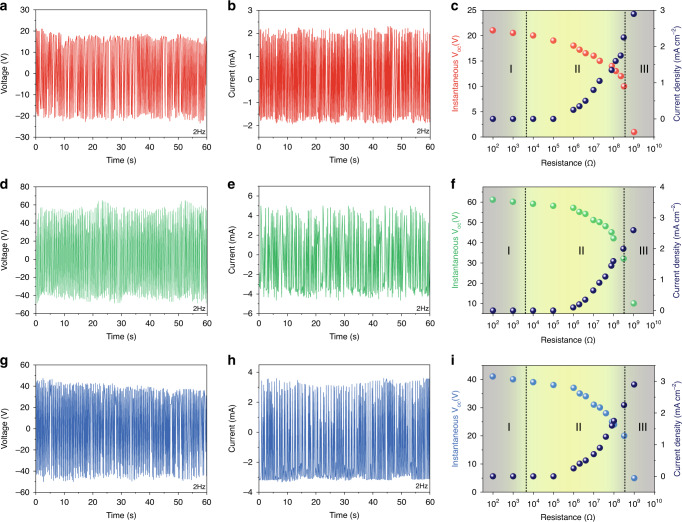
The electrical performance under different aspect ratios. **a**, **d**, **g** The open-circuit voltages of Pem-iTENG with different aspect ratios. **b**, **e**, **h** The short-circuit currents of Pem-iTENG with different aspect ratios. **c**, **f**, **i** The transient voltage and current density of the Pem-iTENG at the external resistance of 10^2^ Ω–10^9^ Ω.

In our experiments, our Pem-iTENG with the aspect ratio (25) of bionic cilia exhibits the highest open-circuit voltage (64.2 V) (Fig. 4a, d, g) and short-circuit current (5.1 mA) (Fig. 4b, e, h). These values are much higher than that of the reported solid-liquid contact based TENGs (Supplementary Fig. 7). The self-growth of bionic cilia on the surface of the PDMS film causes the conversion of the triboelectric interface from 2D to 3D, which improves the surface solid-liquid contact state. When the aspect ratio of bionic cilia decreases from 47 to 13, the short-circuit current increases from 2.4 mA to 4.5 mA and then decreases to 2.5 mA (Fig. 4b, e, h). Our Pem-iTENG exhibits a stable output performance after 125 cycles.

By connecting with the external loads from 10^2^ Ω to 10^9^ Ω, different resistive characteristics including voltage and current density are presented under different aspect ratios of bionic cilia (Fig. 4c, f, i). It is observed that the curve characteristic of each pattern under the external loads is consistent with Ohm’s law. When the external resistance is in working area I (10^2^ Ω−10^4^ Ω), the resistance value is far less than the internal resistance of Pem-iTENG, which is in a short-circuit current state. When the external resistance is in working area II (10^4^ Ω–10^9^ Ω), the external resistance value is equivalent to the internal resistance of Pem-iTENG, and it greatly restricts the transfer charges between the two electrodes. The amount of transfer charges in working area II is lower than that in working area I. Hence, with increasing external loads, the output current drops and the output voltage rises in working area II. When the external resistance is in working area III (>10^9^ Ω), the external resistance is far greater than the internal resistance of Pem-iTENG. The output voltage of Pem-iTENG is in an open-circuit voltage state. As known, the maximal power density is located at the external resistance matching with the internal resistance of TENGs. The maximal power density under the three groups of Pem-iTENG is different, and this is the reason why output performance of Pem-iTENG changes under different aspect ratios of bionic cilia.

### Performance of Pem-iTENG for water wave harvesting

**Fig. 5:**
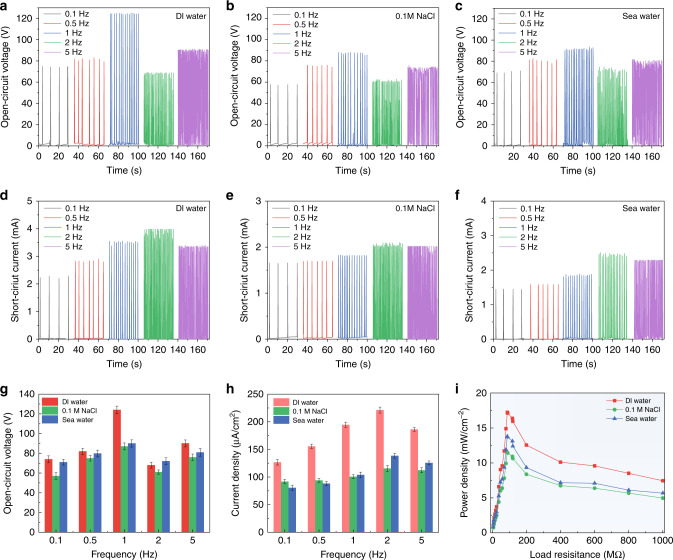
Effects of water wave on the electrical performance. **a**, **d** The effects of DI water on the electrical performance of Pem-iTENG. **b**, **e** The effects of 0.1 M (mol/L) NaCl solution on the electrical performance of Pem-iTENG. **c**, **f** The effects of sea water on electrical output performance of Pem-iTENG. **g**, **h**, **i** The frequency dependence of the open-circuit voltage, short-circuit current density and power density^37^ of Pem-iTENG working in three liquids, respectively.

Here, a linear motor was used to simulate water wave with operating frequencies from 0.1 to 5 Hz. For deionized (DI) water, open-circuit voltage first increases and then fluctuates with increasing water wave frequency, and the highest peak value is ~124.2 V at 1 Hz (Fig. 5a). For NaCl solution and sea water, the change trend of open-circuit voltage is similar to that of DI water (Fig. 5b, c). The peak voltage depends strongly on the charge density in contact electrification. The solid-liquid contact area between water and Pem-iTENG in contact electrification is variable when the water wave frequency is lower than 1 Hz. Hence, the peak voltage is obtained at 1 Hz because the water wave can cover the entire area of Pem-iTENG. However, when the contact water wave frequency continues to increase (>1 Hz), the charge density with opposite polarities on Pem-iCM and liquid surface is easily neutralized due to the parts of liquid retained on Pem-iCM, resulting in fluctuation of the peak voltage^37^. Consequently, the Pem-iTENG exhibits the largest peak voltage at 1 Hz in our experiment. The short-circuit current shows a trend of first increasing and then decreasing with the increasing of the water wave frequency (Fig. 5d–f). Therefore, the electrical performance can be optimized by adjusting the water wave frequency of Pem-iTENG.

Figure 5g, h shows the impact of frequency on the electrical performance of the Pem-iTENG working in three liquids (DI water, 0.1 M NaCl solution, sea water). It can be seen that Pem-iENG operating in 0.1 M NaCl solution exhibits the smallest open-circuit voltage and current density in the three liquids during the solid-liquid sliding process. The output electrical performance of Pem-iTENG gradually increases, when it works in sea water and DI water, respectively. The experimental result illustrates that the impurity concentration in liquid will affect the output performance of Pem-iTENG^19,32^. Hence, Pem-iTENG working in DI water and sea water will exhibit better capacity of harvesting water wave energy compared with that working in 0.1 M NaCl solution.

Hence, Pem-iTENG working in DI water shows the maximal output power density (17.23 mW cm^−2^) (Fig. 5i), open-circuit voltage (124.2 V) and the short-circuit current density (221.6 μA cm^−2^).

### Energy conversion efficiency and its application

**Fig. 6:**
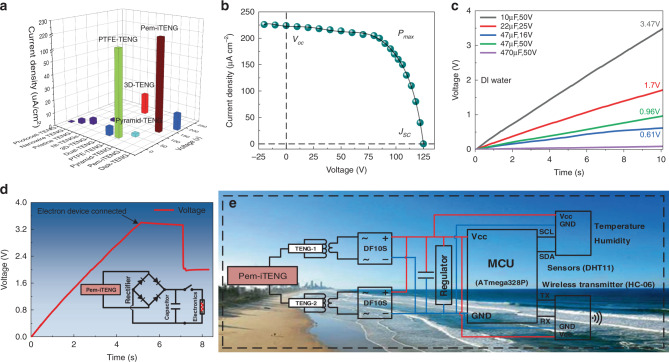
Power conversion efficiency and application of Pem-iTENG. **a** Comparison of electrical performance between Pem-iTENG and reported solid-liquid contact based TENGs. **b** Current density-voltage (*J-V*) curve of Pem-iTENG under solar light intensity and tidal waves. **c** Voltage curves of charging five commercial capacitors using Pem-iTENG, respectively. **d** Charging curve for a 0.47 μF capacitor. **e** Schematic diagram of the circuit of a self-powered wireless environmental monitoring system.

Results show that 3D-TENG, PTFE-TENG, and pyramid TENG exhibit outstanding electrical performance in the reported solid-liquid contact based TENGs^37–45^ (Fig. 6a and Supplementary Fig. 7 and Supplementary Note 3). Compared with the above three TENGs, our Pem-iTENG not only reaches the highest open-circuit voltage but also achieves a breakthrough in short-circuit current, which is nearly ten times larger than that of 3D-TENG (Fig. 6a). The results of these electrical performance are mainly attributed to the efficient energy conversion efficiency of the photoelectric-electromechanical integrated system.

To evaluate energy conversion capacity of our Pem-iTENG, the current density- voltage (*J-V*) curve under various external loads is recorded, and the power conversion efficiency (*PCE*) is calculated referring to solar cells^35,46–49^ in Eqs. (2)–(3) (Fig. 6b). Hence, our Pem-iTENG exhibits a high energy conversion efficiency of 16.72% under sunlight intensity and tidal waves. Meanwhile, we also calculated the *PCE* values of the above three TENGs according to the same method (Supplementary Table 3 and Supplementary Note 4). The power conversion efficiencies of Pem-iTENG, PTFE-TENG, 3D-TENG, and pyramid TENG are 16.72%, 12.59%, 7.33%, and 10.69%, respectively (Supplementary Fig. 8 and Supplementary Note 5). The *PCE* of Pem-iTENG is ~2.28 times higher than 3D-TENG. This clearly demonstrated that our Pem-iTENG exhibits excellent energy conversion efficiency. The improvement energy conversion efficiency in our Pem-iTENG provides a new idea for boosting the output performance of TENGs by the comprehensive and efficient utilization of various natural energies in the future.

Figure 6c shows the output power of Pem-iTENG to charge five capacitors through a commercial rectifier bridge. The speed using Pem-iTENG to charge the capacitor decreases with increasing capacity. When the capacitor is of constant capacitance value (for example, the capacitance value of 47 μF), the higher the rated voltage is, the slower the charging speed is. Among these reported TENGs, our Pem-iTENG was the fastest to charge a 10 μF capacitor, charging to 3.5 V within 10 s. In order to test the power supply capability of our Pem-iTENG to electronic device, a 10 μF capacitor can be charged to 3.3 V within 6 s, then it is used to drive the wireless transmitter to send a trigger signal (Fig. 6d).

In addition, we designed a self-powered wireless environmental monitoring system. It can be integrated into the network to capture coastal tidal energy and solar energy effectively (Fig. 6e, Supplementary Fig. 9, Supplementary Note 6 and Supplementary Movie 1). This system can service for wireless transmission and temperature- humidity monitoring devices, which are convenient to provide coastal residents with real-time marine environment conditions. Meanwhile, this system can also be placed in the water to supply power for life-saving equipment to reduce the occurrence of marine accidents. Overall, our Pem-iTENG is low-cost and universal, which can be supplied to offshore power consumption units, such as lighthouses, buoys and water temperature detection in aiding workers at sea.

## Conclusion

In summary, we proposed a bioinspired Pem-iTENG based on solid-liquid contact electrification. It can comprehensively utilize solar energy and tidal energy and realize high energy conversion efficiency. The enhanced output performance of Pem-iTENG is attributed to the increase of its surface charge density due to the accumulation of photoelectrons from photocatalysis and the triboelectric negative charges from contact electrification. Compared with the electrical performance of reported TENGs based on solid-liquid contact electrification, our Pem-iTENG has a maximal open-circuit voltage of 124.2 V, and a maximal short-circuit current density of 221.6 μA cm^−2^ under tidal waves and sunlight, which represents an improvement by nearly a factor of 10. More importantly, by referring to the method of evaluating *PCE* in solar cells, our Pem-iTENG exhibits a high energy conversion efficiency of 16.72%, which is enhanced by a factor of nearly 2.28 compared with 3D-TENG. This study demonstrates a high-efficiency photoelectric-mechanical integrated system and proposes new attempts for the comprehensive utilization of natural energies developing high-performance TENGs. And it also has great potential for long-term self-powered marine equipment such as lighthouses, buoys, and water temperature detectors.

## Methods

### Materials

Polydimethylsiloxane (PDMS, sylgard184) was purchased from Dow Corning Co., Ltd. Cobalt powder (particle size 300 nm, 800 nm, 1300 nm) was purchased from Alibaba. Nanoscale titanium dioxide (TiO_2_, particle size 30 nm) and polyaniline (PANI, doping rate >30%, particle size <30 μm) were purchased from Queer Biology Co., Ltd. Xylene (AR analytical purity, content no <99.0%) and anhydrous ethanol (mass fraction > 99.7%) were purchased from Tianjin Fuyu Fine Chemical Co., Ltd. (Tianjin, China). A Nd-Fe-B magnet (Nd_2_Fe_14_B, 50 × 50 × 30 mm) was purchased from Hengchang Trading Strong Magnet Co., Ltd. (China). A xenon light source (CEL-S500) was purchased from Beijing Zhongjiao Jinyuan Technology Co., Ltd. A Keithley 6514 electrometer was used to record the output electrical performance (current and voltage) of Pem-iTENG. Under an optical microscope (XTL-500, Guiguang Instrument Manufacturing Co., Ltd.), the top view of the samples was characterized by the uEye lens.

### Preparation of bioinspired Pem-iCM

Super-hydrophobic Pem-iCM was grown on the surface of the substrate (copper plate) by a template-free process. Then, the surface of bionic cilia was modified by the adsorption of nanoscale TiO_2_/PANI. TiO_2_ is adsorbed onto the surface of bionic cilia. After that, situ polymerization of aniline was carried out using the template of the cilia/TiO_2_ composite films. Finally, Pem-iCM (cilia/TiO_2_/PANI) was prepared and discussed (for details, Supplementary Fig. 1 and Supplementary Note 1).

### Calculation of power density under illumination

The power density of Pem-iTENG can be calculated as follows.

1 $$P = \frac{{VI}}{S}$$

Whereby, *V* and *I* are the peak output voltage and current values under various load resistances, respectively, and *S* is the effective area (i.e., 9 cm^2^).

### Calculation method of power conversion efficiency (*PCE*)

Here, the method of calculating *PCE* for solar cells is adopted to evaluate the energy conversion efficiency of Pem-iTENG, as described below in Eqs. (2) and (3).

2 $$FF \,=\, \frac{{V_{\mathrm{{max}}} \times J_{\mathrm{{max}}}}}{{V_{\mathrm{{oc}}} \times J_{\mathrm{{sc}}}}} \times 100\%$$

Whereby, *FF* stands for the fill factor, which is a physical quantity that evaluates the external load capacity of TENGs. The capacity of the external load of the device can be compared according to the value of *FF*. The relationship between the *PCE* and the fill factor (*FF*) is as follows.

3 $$PCE(\% ) \,=\, \frac{{P_{{{\max }}} \times 100\% }}{{P_{\mathrm{{in}}}}} = \frac{{V_{\mathrm{{oc}}} \times J_{\mathrm{{sc}}} \times FF}}{{P_{\mathrm{{in}}}}} \times 100\%$$

Whereby, *P*_max_ is the maximal output power of the device under external loads. Open-circuit voltage (*V*_oc_) refers to the potential between the two electrodes of the device when the external circuit is open-circuited. Short-circuit current density (*J*_sc_) refers to the current value through the device when the external circuit is short-circuited. *P*_in_ is the input power of the device.

## Supplementary information

Supplementary Information

Peer Review File

Supplementary Movie 1

Description of Additional Supplementary Files

## Data Availability

The data that support the plots within this paper and other findings of this study are available from the corresponding author upon reasonable request.
