# Supplementary material for: A high-efficiency bioinspired photoelectric-electromechanical integrated nanogenerator
Source: Nat Commun. 2020 Dec 2;11:6158. doi: 10.1038/s41467-020-19987-0 (PMC7710745; doi:10.1038/s41467-020-19987-0)
Supplement: Supplementary file 1 — Supplementary Information [file 41467_2020_19987_MOESM1_ESM.pdf]

Supplementary Materials

A high-efficiency bio-inspired photoelectric-electromechanical integrated nano-generator

Zhuangzhi Sun *et al*

Brief description of what this file includes:

Supplementary Fig. 1. Preparation of Pem-iCM and self-assembly of Pem-iTENG.

Supplementary Fig. 2. XRD/FT-IR characterization of self-growing Pem-iCM.

Supplementary Fig. 3. Solid-liquid contact angles of different samples**.**

Supplementary Fig. 4. Self-degradation mechanism of organic impurities of Pem-iTENG.

Supplementary Fig. 5. Charge density diagram under different experimental conditions.

Supplementary Fig. 6. The light effect experiments under dark/light cycle.

Supplementary Fig. 7. Comparison of electrical performance of reported TENGs.

Supplementary Fig. 8. Comparison of PCE between Pem-iTENG and reported TENGs.

Supplementary Fig. 9. Design of self-powered wireless environment monitoring system.

Supplementary Table 1. Size parameters of bionic cilia samples induced by magnetic particles.

Supplementary Table 2. The charge density of Pem-iCM before and after illumination.

Supplementary Table 3. PCE parameters of Pem-iTENG and reported TENGs.

Supplementary Note 1. Preparation of Pem-iCM with bionic cilia.

Supplementary Note 2. Self-assembly of solid-liquid contact-based Pem-iTENG.

Supplementary Note 3. Electrical performance of solid-liquid-based TENGs.

Supplementary Note 4. The total input power of Pyramid TENG.

Supplementary Note 5. PCE calculation of developed Pem-iTENG.

Supplementary. Note 6. Details of self-powered wireless environment monitoring system.

Supplementary Figures

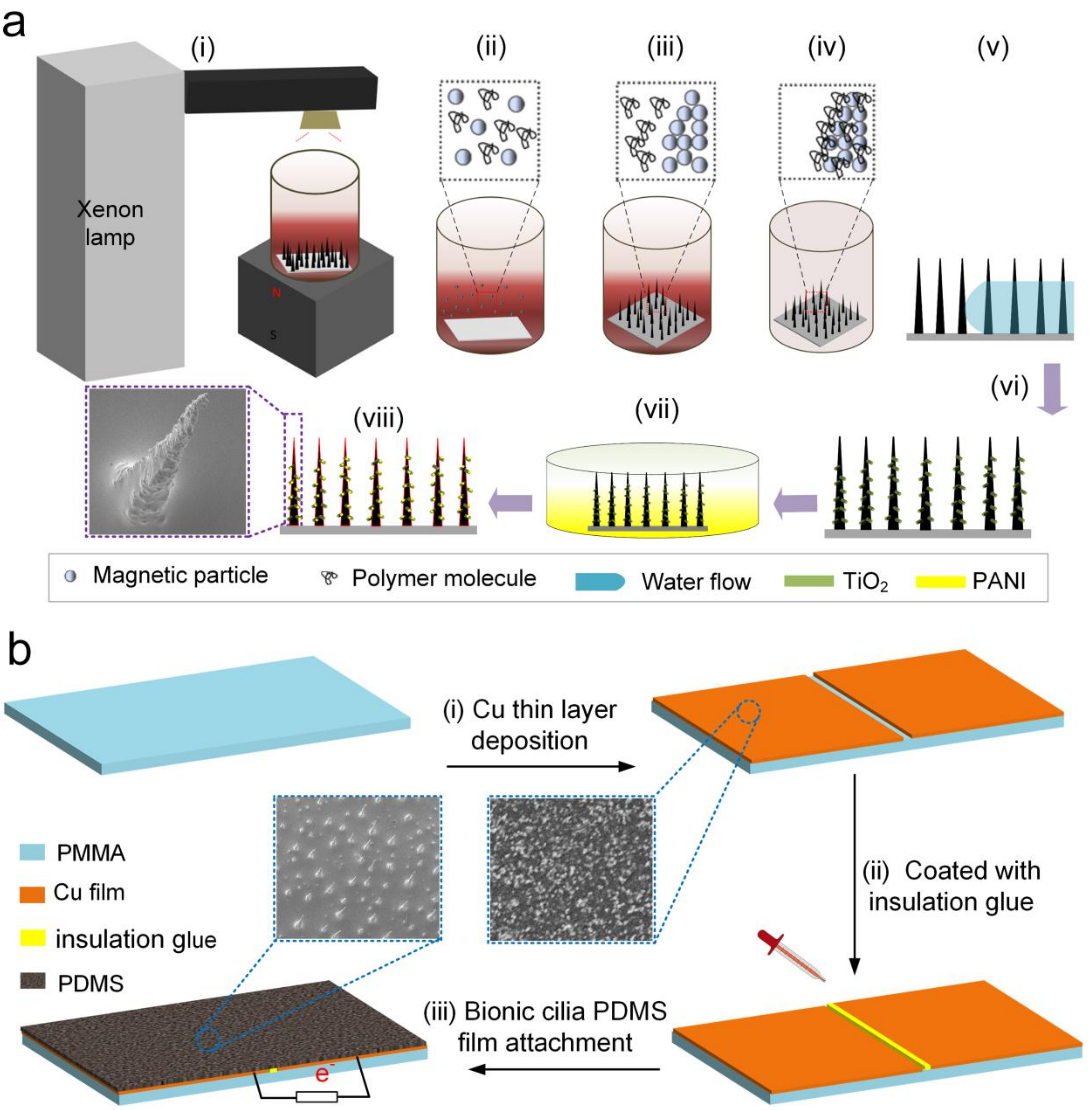

Supplementary Figure 1. Preparation of Pem-iCM and self-assembly of Pem-iTENG. a The preparation process diagram of Pem-iCM: (i) schematic diagram of the improved experimental device (heating with xenon lamp), (ii) initial mixed state (no magnetic field), (iii) phase separation state (magnetic field direction perpendicular to the substrate), (iv) state of solvent after complete volatilization (no magnetic field), (v) functional bend (flow at any angle), (vi)~(viii) are the flow chart of TiO_2/PANI on the surface of cilia. **b** The assembly process of Pem-iTENG: (i) paste the copper film on PMMA board, (ii) apply insulating glue on the edge of the copper film, (iii) the Pem-iCM with micro/nano scaled bionic cilium is attached to the Cu electrode.

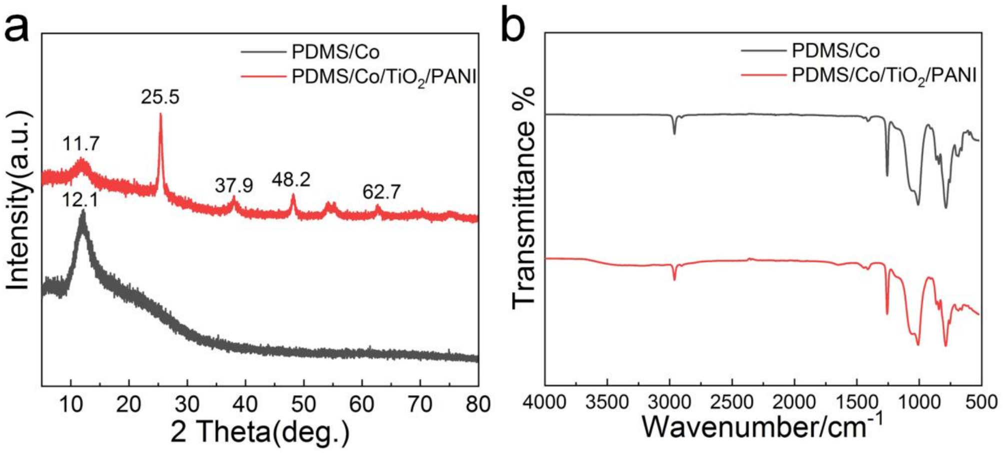

Supplementary Figure 2. XRD/FT-IR characterization of self-growing Pem-iCM. a XRD diagram of Pem-iCM， **b** FT-IR diagram of Pem-iCM.

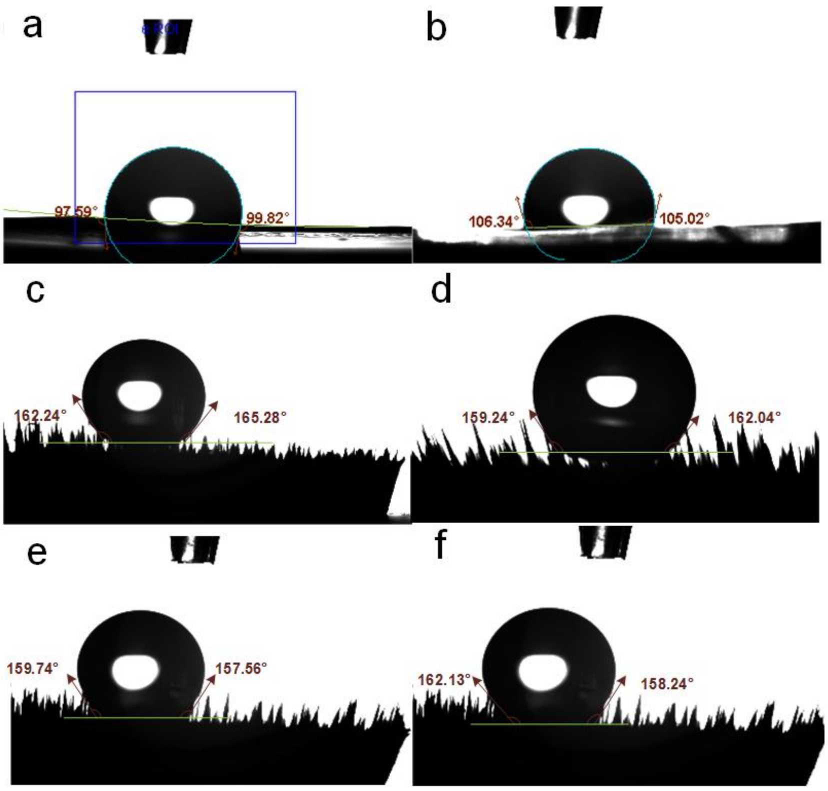

Supplementary Figure 3. Solid-liquid contact angles of different samples. a The contact angle of pure PDMS, **b** The contact angle of the PDMS doped with cobalt powder, **c** The contact angle of PDMS with bionic cilia unmodified TiO_2/PANI, **d** The contact angle of PDMS with bionic cilia modified TiO_2/PANI, **e** The contact angle of PDMS with bionic cilia modified TiO_2/PANI after illumination for 1 h, **f** The contact angle of PDMS with bionic cilia modified TiO_2/PANI after illumination for 3 h.

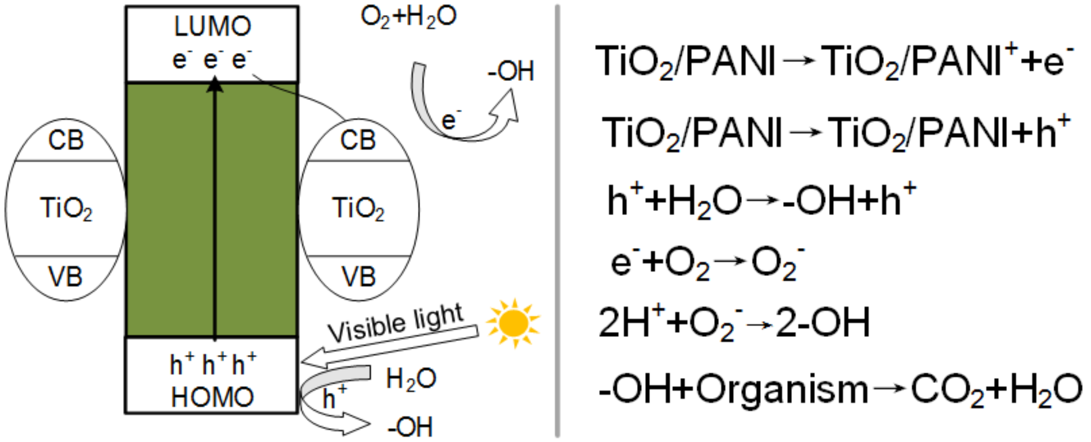

Supplementary Figure 4. Self-degradation mechanism of organic impurities of Pem-iTENG.

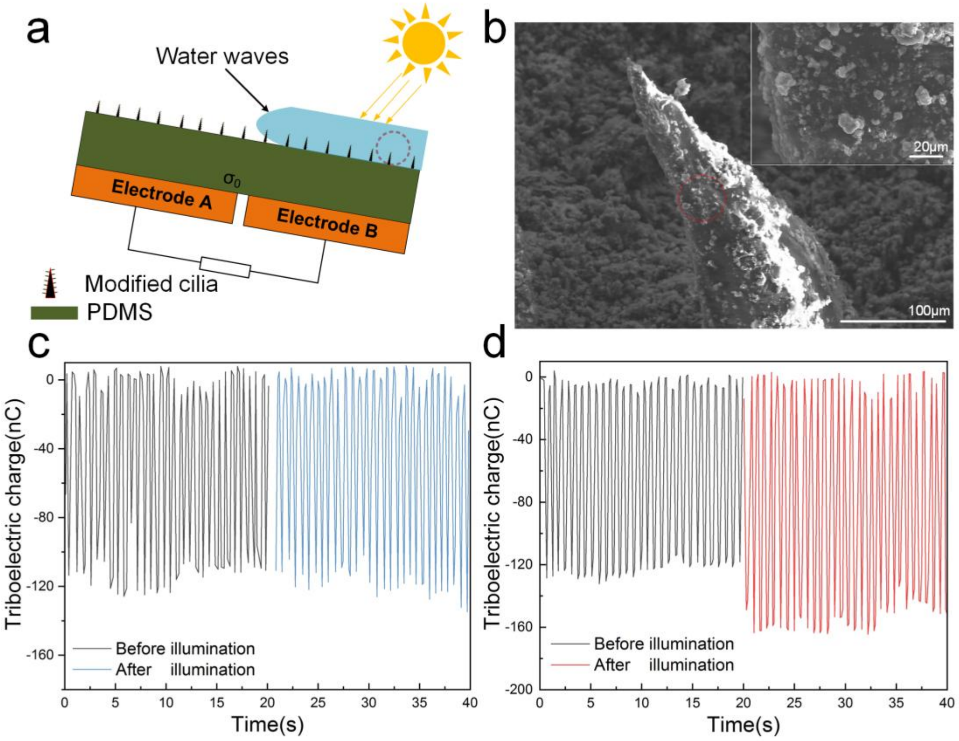

Supplementary Figure 5. Charge density diagram under different experimental conditions. a simple structure diagram of Pem-iTENG, **b** Single bionic cilia modified TiO_2/PANI, **c** Charge density unmodified TiO_2/PANI before and after the illumination, **d** Charge density modified TiO_2/PANI before and after the illumination.

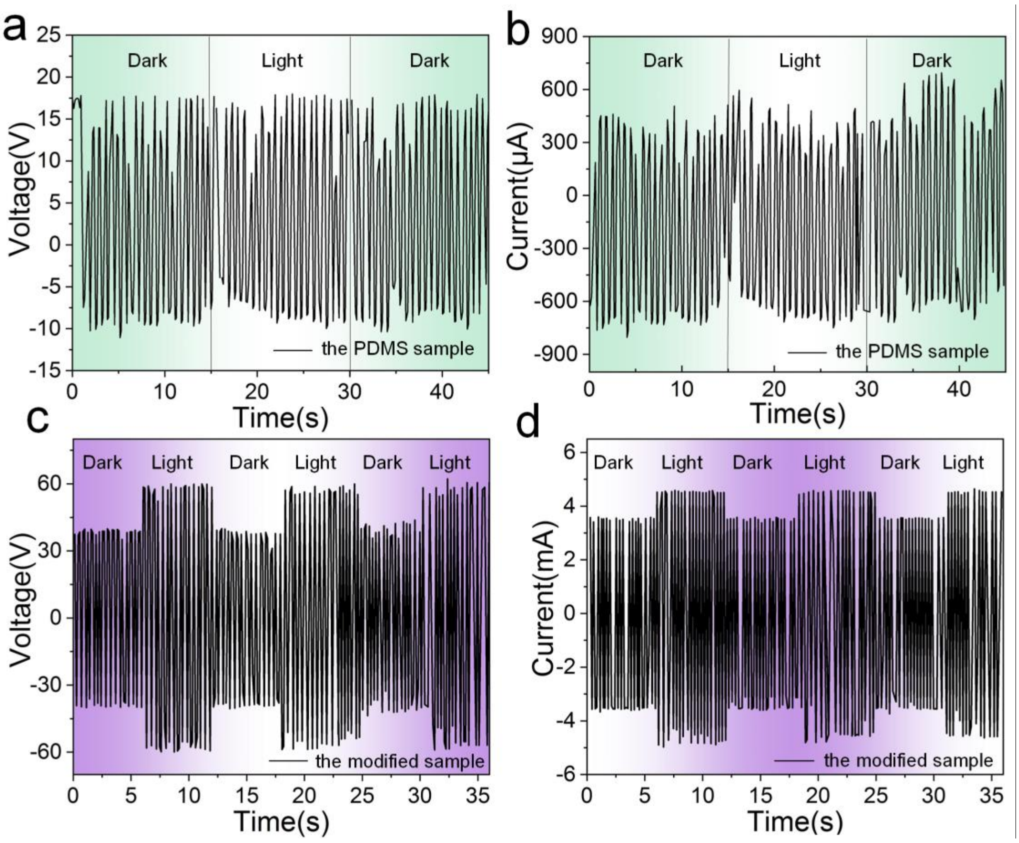

Supplementary Figure 6. The light effect experiments under dark/light cycle. a Open circuit voltage and short circuit current **b** of PDMS without bionic cilia unmodified TiO_2/PANI, **c** Open circuit voltage and short circuit current **d** of PDMS with bionic cilia modified TiO_2/PANI.

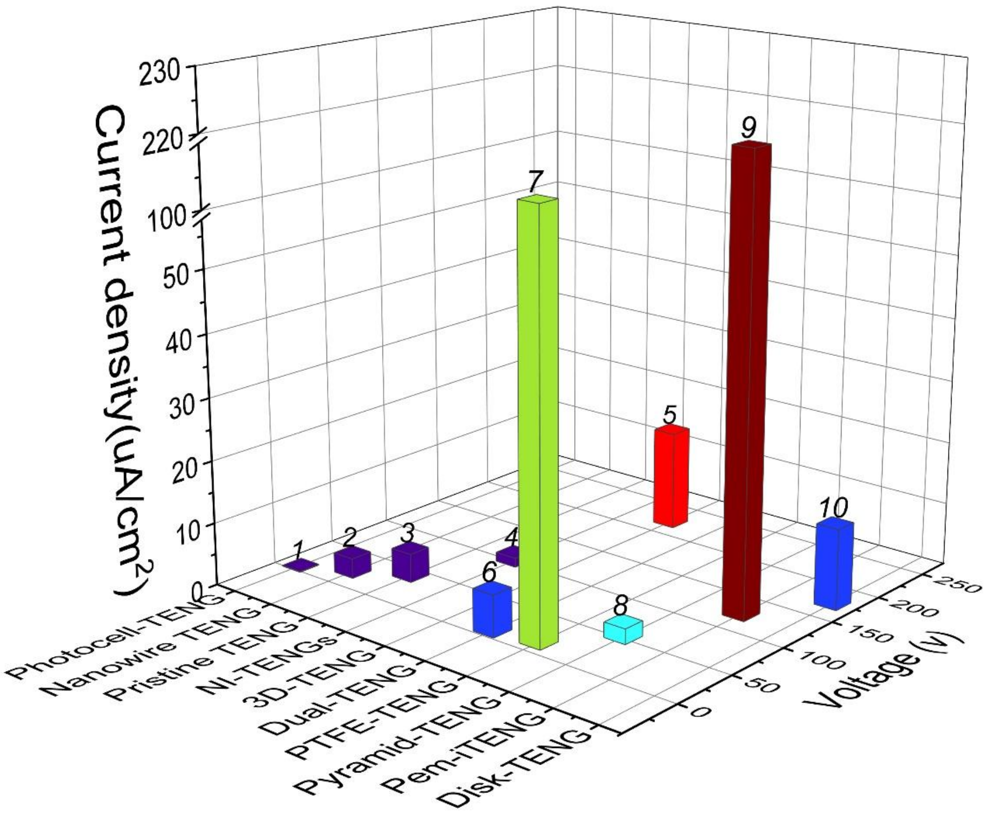

Supplementary Figure 7. Comparison of electrical performance of reported TENGs.

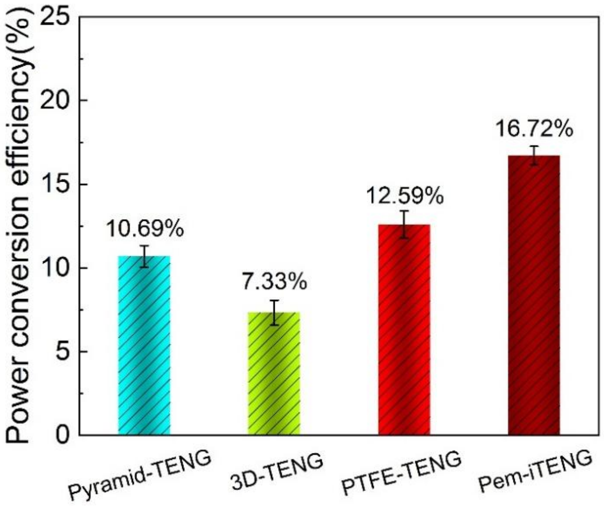

Supplementary Figure 8. Comparison of PCE between Pem-iTENG and reported TENGs. Pyramid-TENG (10.69% ±0.65%), 3D-TENG (7.33% ±0.74%), PTFE-TENG (12.59% ±0.81%), PTFE-TENG (16.72% ±0.54%).

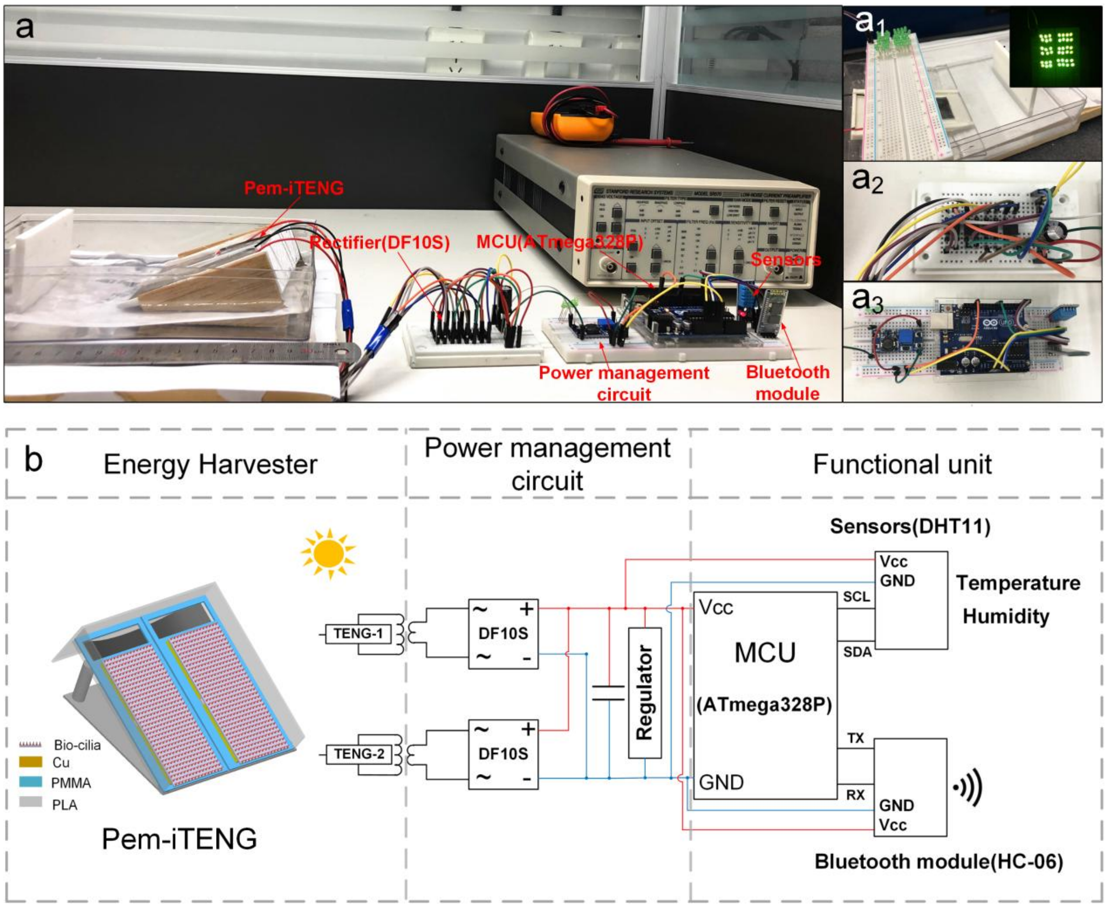

Supplementary Figure 9. Design of self-powered wireless environment monitoring system. a The experimental prototype manufacturing. **b** The system circuit diagram.

Supplementary Tables

Supplementary Table 1. Size parameters of bionic cilia samples induced by magnetic particles.

**Magnetic particle size (nm)**	300	800	1300
**Density (N·cm^{-2})**	~945	~630	~298
**Spacing (μm)**	~278	~373	~465
**Length (mm)**	~1.35	~1.15	~0.86
**Diameter (μm)**	~28.5	~45	~64
**Aspect ratio**	~47	~25	~13

Supplementary Table 2. The charge density of Pem-iCM before and after illumination.

**Materials**	**Illumination state [Dark/light]**	**Current [mA]**	***Q_{sc}* [nC]**	**Charge Density [nC cm^{-2}]**
Unmodified TiO_2/PANI	D	3.42	120.56	13.395
Unmodified TiO_2/PANI	L	3.49	121.11	13.457
Modified TiO_2/PANI	D	3.53	128.41	14.267
Modified TiO_2/PANI	L	4.52	164.67	18.296

Supplementary Table 3. PCE parameters of Pem-iTENG and reported TENGs.

TENGs	V_{oc} [V]	J_{sc} [μA cm^{-2}]	P_{max} [mW cm^{-2}]	FF [%]	P_{in} [mW cm^{-2}]	PCE [%]
**Pyramid TENG**	52	0.245	0.005	39.24	0.0467	10.69
**3D-TENG**	221	16.1	2.2	61.18	30	7.33
**PTFE-TENG**	13.96	106.18	0.962	64.90	7.64	12.59
**Pem-iTENG**	124.2	221.6	17.23	62.60	103.012	16.72

Supplementary Notes

Supplementary Note 1. Preparation of Pem-iCM with bionic cilia.

The fast template-free method is used to grow magnetic bionic cilia on the surface of substrate (glass plate, aluminum foil or others). When an external magnetic field is applied, the self-growth array structured cilia forms rapidly in a bottom-up in the solution containing magnetic nanoparticles and elastic polymers. The prepared cilium is millimeter grade, and its aspect ratio can even exceed 100. Then, P-N heterojunction (formed by TiO_2/PANI) is planted on the surface of bionic cilia by layer by layer self-assembly method which is based on the opposite charge of polyanion and polycation, and the electrostatic attraction of positive and negative charges is the main driving force.

Supplementary Note 2. Self-assembly of solid-liquid contact-based Pem-iTENG.

The self-assembly process of Pem-iTENG with bionic cilia is shown in Supplementary Fig. 1b. Firstly, 1 mm thick PMMA is cut into 8×4 cm (L×W) by laser cutting machine. On the PMMA substrate, two areas of 3.5×3.5 cm (L×W) are pasted with copper film (0.1 mm thickness). Two areas of deposited copper film are isolated and non-conductive, which are used as the positive and negative poles of the independent layer typed nano-generator. Pem-iCM is then attached to the top of the copper film. Finally, Pem-iTENG is fixed in the water tank, and a linear motor (torque range 0.5-10N) is used to simulate the water wave fluctuation. Under the periodic fluctuation of simulated seawater, the water wave continuously washes Pem-iTENG (see Fig.1 for the working principle of Pem-iTENG). The maximal angle between the base plate (PMMA) and the water tank is 20°, and the volume of liquid in the water tank is set as 100 mL, which is equivalent to 40 % of the total volume of Pem-iTENG. The experiment is carried out at 25 °C. In addition, light passes through a 10 mm diameter circular hole under the xenon lamp is used to irradiate Pem-iCM surface. The relative position of the light source is fixed to ensure that the light intensity is the same after each light passes through the hole. Finally, copper metal is the fixed electrode of Pem-iTENG, and the output end of the electrode is connected to the test equipment via a rectifier bridge.

Supplementary Note 3. Electrical performance of solid-liquid-based TENGs.

In order to demonstrate the outstanding advantages of Pem-iTENG, which can collect marine energy and solar energy at the same time, we compared the electrical performance of Pem-iTENG with nine traditional solid-liquid-based TENG (as shown in Supplementary Fig. 4).

1. Photocell-TENG is made of super-hydrophobic PDMS and ITO/PEN substrate. The current of the output voltage is 7 V and 128 nA, respectively. The maximal output power is 0.27 μW.

2. Nanowire-TENG is a kind of nanowire structure, which is grown on the surface of friction film by hydrothermal synthesis. The open-circuit voltage of the nanogenerator can reach 21.3 V, and the short-circuit current can reach 3.2 μA cm^{-2}.

3. For Pristine-TENG without PDMS surface treatment, the open-circuit voltage and the short-current density are 40 V and 4.6 μA cm^{-2}, respectively.

4. For the meshed integrated triboelectric nanogenerator (NI-TENG), the surface morphology composed of dense nanowire arrays. NI-TENG can stably generate the short-circuit current of 1.5 μA cm^{-2} and the open-circuit voltage of 101 V in water.

5. For the three-dimensional structure TENG with super-hydrophobic interlayer (3D-TENG), it shows high adaptability to humidity and environmental pollution. They used particle lithography technology to prepare super-hydrophobic interlayer with three-dimensional (3D) porous pattern, resulting in 161° high static water contact angle, the open-circuit voltage up to 221 V, and the short-circuit current density of 16.1 μA cm^{-2}.

6. For the hybrid triboelectric nano-generator (Dual-TENG), the electrostatic energy and mechanical energy of flowing water can be collected at the same time. The Dual-TENG is mainly composed of super-hydrophobic TiO_2 layers with layered micro/nano structure, which is used to collect the electrostatic energy of water (output 1), and the Contact-TENG, which is composed of polytetrafluoroethylene film and a layer of nano-level SiO_2, is used to collect the mechanical energy of water (output 2). The short-circuit current generated by output 1 and output 2 of Dual-TENG can reach 43 μA and 18 μA, respectively.

7. For PTFE-TENG, the original PTFE can reach a short-circuit current density of 106.186 μA cm^{-2} at a contact frequency of 0.1 Hz.

8. For PDMS friction film surface modified by pyramid pattern (Pyramid-TENG), it can provide the open-circuit voltage of 52 V and the short-circuit current density of 0.245 μA cm^{-2}. The peak power density is closed to 1.3 W m^{-2}, and can drive 60 LEDs at the same time.

9. The maximal output open-circuit voltage and short-circuit current of Pem-iTENG proposed in this paper can reach 124.2 V and 221.6 μA cm^{-2}, respectively.

10. Disk-TENG is composed of the water turbine-TENG and the solid-liquid contact TENG of the water turbine blade. The water turbine blade is the solid-liquid contact surface, which can

obtain the impact energy and the solid-liquid contact friction energy from the tap water of the household. The open-circuit voltage of water turbine TENG and Disk TENG is about 72 V and 102 V, and the short circuit-current is about 12.9 μA cm^{-2} and 3.8 μA cm^{-2}.

Supplementary Note 4. The total input power of Pyramid TENG.

Since the specific input parameters of the total input power (P_{in}) is missed in the report, the output power value of 60 LED (electrical parameters used in the paper) is adopted as the total input power value of Pyramid TENG. In this way, the calculated result of Pyramid TENG (10.69%) is slightly higher than the real PCE value (Supplementary Table 3).

Supplementary Note 5. PCE calculation of developed Pem-iTENG.

The total input power (P_{in}) of Pem-iTENG can be calculated using the formula of $P_{in} = P_m / S + P_{light}$. Whereby, P_m is the output power of the linear motor that simulates water waves (3.5 W), S is the area of the water wave baffle (50 cm^2). P_{light} is the illumination intensity of solid-polymer surface when the xenon lamp simulates solar illumination (33.012 mW cm^{-2}). Hence, the total input power of P_{in} can be calculated as 103.012 mW cm^{-2} (Supplementary Table 3).

Supplementary Note 6. Details of self-powered wireless environment monitoring system.

A linear motor (Linmot E1100) was used to drive seawater forward and backward to simulate wave motion, and the maximal continuous sliding displacement was 15 mm. The relative symmetrical acceleration of the Pem-iCM was ± 20 m $s^{-2,}$ and the maximal speed was 0.55 m s^{-1}. The open-circuit voltage and the short-circuit current were measured by a programmable electrometer (Keithley 6514).

A typical structure of a composite power generation unit, which adopts an independent layer operation mode, was applied for collecting blue energy. The device was composed of two parts. The upper part was Pem-iTENG. In this structure, Pem-iCM was not only a friction layer for water wave energy to electric energy conversion but also a layer for light to electric energy conversion. Cu film formed the electrode layer. The lower part was the supporting structure, which was constructed by 3D printing technology and had the characteristics of light weight and corrosion resistance. The total transverse dimension of the device was 12×9 cm^2, and the actual effective contact area between the water flow and Pem-iCM with bionic cilia was 8×7 cm^2. The electric energy output by Pem-iTENG was not suitable for directly supplying power to electronic equipment. On the one hand, Pem-iTENG periodically generates alternating current. On the other hand, for most self-powered sensors, TENG only needs to supply power to the sensor for only a short time to enable the working mode, while the sensor is in sleep mode most of the time. Therefore, during the sensor sleep mode, the energy collected by Pem-iTENG can be rectified and stored in an energy storage device (such as a battery or capacitor), and when the sea level fluctuates at low frequency, the stored energy of the capacitor can be used to power the device for sensing, data processing and wireless signal transmission.

As shown in Supplementary Fig. 5b, a self-powered wireless environmental monitoring system supported by Pem-iTENG was developed to observe the stability parameters of seawater. The system consisted of three parts: the energy collection device, the power management power, and the functional components. The energy collection device converted tidal energy and solar energy into electrical energy. The power management circuit (PMC) with an energy storage unit supplies power for a self-powered wireless sensor. The PMC consisted of a transformer, rectifier, voltage regulator, supercapacitor and switch. The rectified output was connected in parallel to a supercapacitor of 10 μF to store electrical energy. The voltage regulator maintained a 5 V DC voltage to power the electronic equipment. Functional components included the environment monitoring sensor, microcontroller and Bluetooth module. The commercial environment sensor (DHT11, temperature and humidity measuring) was used as a physical sensor node to monitor environmental properties. In addition, a commercial Android board with a micro controller

(Atmega328P) was used as the signal processing unit, and a Bluetooth module (HC-06) was used for wireless communication.
