# Supplementary material for: A high-efficiency bioinspired photoelectric-electromechanical integrated nanogenerator
Source: Nat Commun. 2020 Dec 2;11:6158. doi: 10.1038/s41467-020-19987-0 (PMC7710745; doi:10.1038/s41467-020-19987-0)
Supplement: Supplementary file 4 — Description of Additional Supplementary Files [file 41467_2020_19987_MOESM4_ESM.pdf]

Description of Additional Supplementary Files

File name: Supplementary Movie 1
Description: A self-powered wireless environmental monitoring system
