# Supplementary material for: A high-efficiency bioinspired photoelectric-electromechanical integrated nanogenerator
Source: Nat Commun. 2020 Dec 2;11:6158. doi: 10.1038/s41467-020-19987-0 (PMC7710745; doi:10.1038/s41467-020-19987-0)
Supplement: Supplementary file 2 — Peer Review File [file 41467_2020_19987_MOESM2_ESM.pdf]

Reviewer #1 (Remarks to the Author):

The general scope of the paper is well justified and of interest to a sufficiently broad audience. The main finding of this study appears to comprehensively collect solar energy and tidal energy by introducing a cilia-like structure as well as a p-n heterojunction. There are several issues that I believe need to be addressed before the publishing of this manuscript.

1. Motivation. The advantages of this work lie in the combination of the photoelectric and triboelectric effects. However, the photoelectric effect relies on the sunlight, thus why not harness the solar energy directly since it has much higher energy density. Thus, the motivation of this work should be elaborated.

2. As shown in Fig.2 the surface of the device is superhydrophobic due to the design of cilia, which is used to increase the contact area and more friction as claimed by the authors. In fact, according to the Cassie and Wenzel theory, the superhydrophobic property relies on more air pocket, thus the real contact area between water and solid might be reduced rather than increased. Thus, is the superhydrophobic property is really the optimized design?

3. Mechanisms. As illustrated in Fig.1d, initially at the state 1 the PDMS surface possess plenty of negative charges, the mental electrodes should carry induced charges and there is no output. The output only occurs when the water comes in which dynamically changes the electrical double layer. If the output is indeed from the contact electrification according to what the authors pointed out, the output is actually very low. However, the authors showed that the output without illumination is still about 7 times higher than other devices. Thus, I would tend to think the mechanism might be quite similar to the recent report on the electricity generation based on a droplet (Wanghuai Xu, et al, Nature), in which the droplet gates the charge transfer. I strongly suggest the authors to measure the charge density before and after illumination, as well as the charges generation during the wave simulate test.

4. The stability of the device should be considered, as TiO_2 is always regarded to accelerate degradation of polymer (PDMS in your device).

5. Why the changes of OV and SA are out of sync, wherein the biggest OV appears when frequency of fluctuation is 1 Hz and SA at 2 Hz.

Reviewer #2 (Remarks to the Author):

A bio-inspired liquid-solid photoelectric electromechanical integrated triboelectric nano-generator (Pem-iTENG) is proposed. It is claimed that the electron transfer between water and the modified samples is enhanced by the photocatalysis. A new idea for improving the output performance of the TENGs is presented in this work. However, the experiments in this manuscript is not systematically carried out, and the working mechanism of the Pem-iTENG is not fully convincing. The biggest problem in this work is lack of the controlled experiments to verify the hypothesis. The only evidence is the enhanced output after illuminated by light. As we know that the contact electrification between liquid and solid is very sensitive to the contact angle of the solid surfaces. The modification of TiO_2/PANI, and light irradiation may change the contact angle of the solid surfaces and lead to the increase of electron transfer. Therefore, it is suggested that the contact angle of the PDMS without cilia, TIO_2/PANI modified PDMS with and without light irradiation should be measured to exclude the effect of contact angle on the electron transfer. Also, it is demonstrated that the photon excitation effect of the insulator surfaces may lead to change of the electron transfer between two contact materials. In order to confirm that the increase of electron transfer is induced by photocatalysis instead of photon excitation effect, the light effect experiments (Fig. 3a,3b) of the unmodified sample

surfaces should be tested as a control group. I think this manuscript cannot be published in current version. Following are my additional questions and concerns:

1. In the photocatalytic system (Fig. 1b), the authors claim that the holes tend to be retained in the VB of the TiO2, while the electrons will transfer to cilia root. There are no evidences to support this explanation, because there is no field to separate the electron from the holes. Why the holes react with the water molecules and the impurities in liquid water, and the electrons don’t?
2. I think the working principle of the Pem-iTENG is incorrect in Fig. 1d. Why the water is positively charged in the schematic? Here are two references for authors (Adv. Mater. 26 (2014) 4690; Nano Energy, 36 (2017), 250-259; Adv. Mater. Interfaces, 6 (2019), 1901547.), in which the principle of the liquid-solid TENG is described clearly and correctly.
3. The Pem-iTENG is inspired by the lotus leaf, to obtain the advantages of hydrophobicity, self-cleaning and surface roughness. However, the effects of the hydrophilia and roughness of the TENG on the output performance are not discussed.
4. It is suggested that the surface charge density of the samples in the experiments should be measured or calculated.
5. The academic term for “friction electrification” is “triboelectrification” or “contact electrification”.

Reviewer #3 (Remarks to the Author):

The authors developed a bio-inspired solid-liquid interface contact-based photoelectric-electromechanical integrated triboelectric nano-generator. The energy conversion efficiency can reach 67.4%. The authors contributed this high efficiency to the efficient sunlight capture by PN heterojunction. The topic is interesting, but it seems that there are many experimental results/proposed mechanism which needs carefully being addressed before publication.
1. The diagrams of energy level alignment between PANI and TiO2 in Figure 1b and c are contradictory. In figure 1b, the interface seems like a type Ⅰ heterojuction. But in the figure 1c, the energy alignment of the heterojunction is type Ⅱ. Additionally, I not sure that it is suitable to descript this interface by P-N junction? Polyanine is conducting polymer instead of semiconductor one. And in figure 1c, the bandgap values between PANI and TiO2 are different. But in the picture the distances between CB and VB of two different materials are almost the same. It is contradictory.
2. There are less evidences to show that the efficient charge separation takes place between PANI and TiO2. Such as surface potential difference, photovoltaic effect. Assuming that the carrier separation can take place at the interface, is it high likely that the recombination happens during operation since the seawater can work as a conductor?
3. The calculation of the “average power density” in method part is confusing. What dose the “average” mean? Since the V(t) and I(t) are the functions of the time, is it a time average or something else? Why did the authors use the peak voltage and peak current to conduct the calculation?
4. Following the aforementioned question, it is highly suspected that the energy efficiency calculation is proper used. The authors claims that the power conversion efficiency is as high as 67%. It is a very high value. Here, how do the author define the power conversion efficiency? It means the ratio of electrical power output dividing water mechanical energy as well as adding up the solar (light) illumination? I strongly suggest that the authors refer to the solar cell power conversion efficiency, it is widely accepted and applied both in industry and academy.
5. The authors claims that there are charge trsanfer/exciton dissociation at the interface between PANI and TiO2. However, there is no experiment data either external quantum efficiency or transient optical measurement. Since it is the basic point of this manuscript, I would suggest that authors need to provide solid evidence to verify this point.
6. In the part of energy efficiency calculation, the data used to calculate output electrical energy are collected before rectification or after? Are there any difference between the calculated values by using the data collected before or after rectification? Please give the details. Additionally, what is the value

of the “η”? And why use the “η” during input light energy calculation?

Response to Reviewers' comments:

Reviewer #1:

The general scope of the paper is well justified and of interest to a sufficiently broad audience. The main finding of this study appears to comprehensively collect solar energy and tidal energy by introducing a cilia-like structure as well as a p-n heterojunction. There are several issues that I believe need to be addressed before the publishing of this manuscript.

1. Motivation. The advantages of this work lie in the combination of the photoelectric and triboelectric effects. However, the photoelectric effect relies on the sunlight, thus why not harness the solar energy directly since it has much higher energy density. Thus, the motivation of this work should be elaborated.

Response: Thank you for your comments. Firstly, although solar energy has a higher energy density, it has to be said that the use of solar energy is uncertain, especially under some cloudy and rainy conditions, and solar energy cannot achieve continuous energy collection. Relatively speaking, tidal energy is relatively stable and can be used stably under cloudy and rainy conditions.

Secondly, this work is to propose a photoelectric and electromechanical integrated energy system to improve energy conversion efficiency, and it is oriented to the comprehensive collection of solar energy and tide energy. Although solar energy has a relatively high energy density (especially the development in photovoltaic cells), there is a certain limit to the improvement of its photoelectric conversion rate. Compared with single solar power generation, we believe that the comprehensive utilization of nature energies is a massive potential from the perspective of energy utilization efficiency.

Finally, from the perspective of improving the energy conversion efficiency of the triboelectric nanogenerators, our Pem-iTENG is a new creation. This method is achieved by the superposition of the photocatalysis and the contact electrification to increase the surface charge density. Specifically, the charge density in the PDMS film with bionic cilia modified TiO_2/ PANI is greatly enhanced by the accumulation of photoelectrons in the photocatalysis and the triboelectric negative charges in the contact electrification. This provides a new idea for the further design of solid-liquid

based TENGs in contact electrification.

2. As shown in Fig.2, the surface of the device is superhydrophobic due to the design of cilia, which is used to increase the contact area and more friction as claimed by the authors. In fact, according to the Cassie and Wenzel theory, the superhydrophobic property relies on more air pocket, thus the real contact area between water and solid might be reduced rather than increased. Thus, is the superhydrophobic property is really the optimized design?

Response: Thanks for your comments. From the wetting theory, we know that, the water droplets contact the surface in a wetting mode as if nailed to the surface in the Wenzel state (see Fig s1.1a), while the water droplets contact the surface in a non-wetting style in the Cassie state, and the water droplets are easily rolled away from the surface due to the small adhesion force at this time (see Fig s1.1b).

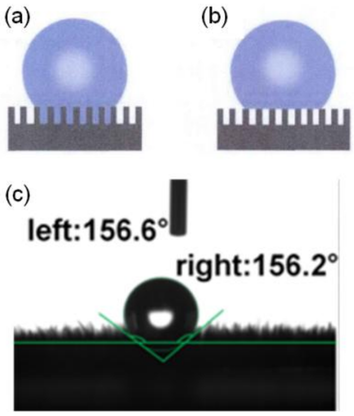

Fig s1.1 wetting theory models: (a) Wenzel state, (b) Cassie state, (c) solid-liquid contact angle of experimental sample.

Since our design is aimed at the rolling friction of the reciprocating motion of tidal water, the main advantages focus on these two aspects. Firstly, the horizontal or inclined surface motion of tidal water is different from the frontal impact of liquid droplets. This horizontal reciprocating motion does increase the air pocket contact in the surface-plane contact compared to the flat-plane contact. Secondly, considering the adhesion force and contact angle hysteresis of the droplets, seawater on the bionic cilia surface can have minimize residue droplets after reciprocating scouring.

Therefore, in order to express our design of superhydrophobic properties in this article more clearly, we have modified the description in the article.

3. Mechanisms. As illustrated in Fig.1d, initially at the state 1 the PDMS surface possess plenty of negative charges, the metal electrodes should carry induced charges and there is no output. The output only occurs when the water comes in which dynamically changes the electrical double layer. If the output is indeed from the contact electrification according to what the authors pointed out, the output is actually very low. However, the authors showed that the output without illumination is still about 7 times higher than other devices. Thus, I would tend to think the mechanism might be quite similar to the recent report on the electricity generation based on a droplet (Wanghuai Xu, et al, Nature), in which the droplet gates the charge transfer. I strongly suggest the authors to measure the charge density before and after illumination, as well as the charges generation during the wave simulate test.

Response: Thanks for your comments. The principle of the solid-liquid contact nano-generator is roughly explained in the following two ways. A working model is based on the fact that liquid droplets constantly strike on the surface of the friction polymer film, and the contact electrification phenomenon occurs (Nano Energy, (2017) 36, 250-259). Another solid-liquid contact model is that the water flow continuously washes the friction polymer film by the principle of electrostatic induction to collect electric charges, and an alternating current is generated (Nano Energy (2014) 9, 186-195). In fact, the working principle of the power generation driven by liquid droplets or water waves is similar, and the output electrical performance is realized by the contact electrification.

From the theoretical point of view, Xu et al in Nature's work is more inclined to the former. Because of the contact electrification between the droplets and the friction polymer film, the alternating current is induced between the electrodes. But our work is the latter, the principle of electrostatic induction collects triboelectric charges by water flow and produces an alternating current between two electrodes. However, it must be said that Xu et al in Nature's work and ours work have their own innovations.

Xu et al proposed a new type of power generation by droplet impact like a triode. This nano-generator consists of a three-layer structure of aluminum (Al) electrode, polytetrafluoroethylene (PTFE) film and indium tin oxide (ITO) electrode, and this structure is very similar to the field effect transistor structure (FET triode). It is composed of three electrodes, a gate, a source, and a drain. The basic principle is to

control the formation of the channel between the source and the drain by adjusting the gate voltage on the gate, and further control the flow of charges between the two electrodes.

Our work mainly proposes a photoelectric and electromechanical integrated energy system. That is to say, the working principle is attributed to the superposition of the photocatalysis and the contact electrification to increase the surface charge density (see Fig.1). Specifically, the charge density in the PDMS film with bionic cilia modified TiO_2/ PANI is greatly enhanced by the accumulation of photoelectrons in the photocatalysis and the triboelectric negative charges in the contact electrification. Subsequently, the asymmetric distribution of induced charges between the two electrodes establishes an unbalanced potential, which drives the transfer of electrons, leading to the generation of the alternating current. The experimental results show that under the tidal waves and sunlight, our Pem-iTENG shows a maximum open-circuit voltage of 124.2 V, and a maximum short-circuit current density of 221.6 μA cm^{-2}, which improves nearly 10 times. More importantly, our Pem-iTENG has higher energy conversion efficiency of 67.4%, which increases nearly 6 times. Therefore, our Pem-iTENG has certain advantages in energy conversion efficiency and comprehensive utilization of different natural energy sources.

In addition, we also carefully read and quoted recent literature on droplet power generation (Wanghuai Xu, et al, Nature) obtaining a lot of inspiration. The author realized a large industry through a small drop of liquid. We believe that this type of droplet power generation will lead the revolution in the solid-liquid contact power generation. We hope that our proposed photoelectric-electromechanical integrated triboelectric nano-generator can also provide a certain reference in this field, and can trigger more inspiration for solid-liquid contact power generation.

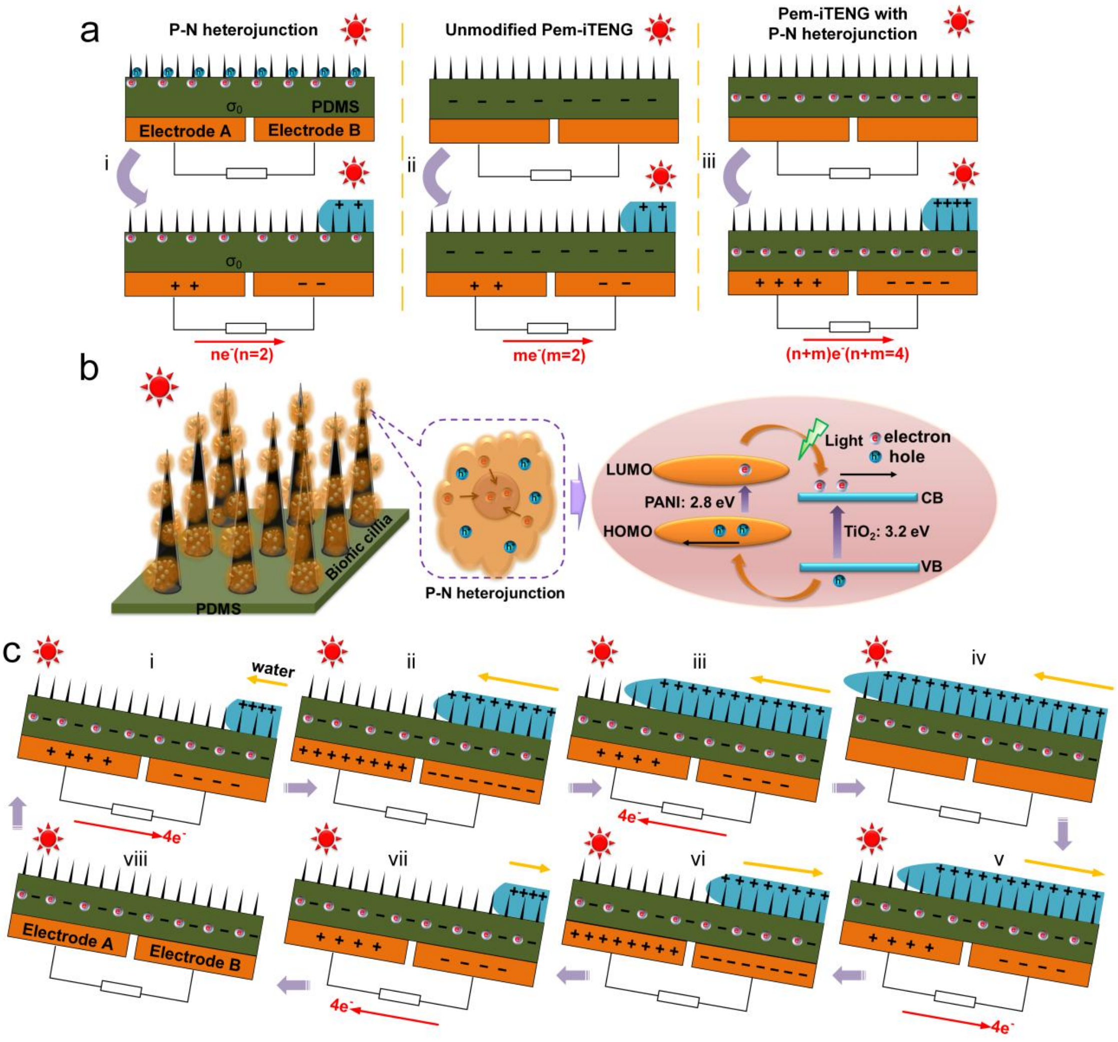

Fig. 1 the working principles of Pem-iTENG. (a) Pem-iTENG with P-N heterojunction (i), unmodified and modified Pem-iTENG during the two flow states of the tidal waves (ii and iii). (b) principles of photocatalysis of bionic cilia with P-N heterojunction. TiO_2 (bandgap 3.2 eV) can only absorb ultraviolet light, while PANI (bandgap 2.8 eV) can absorb both ultraviolet and visible light (c) the contact electrification of Pem-iTENG in a cycle of reciprocating tidal wave, and free electrons transfer direction between the two electrodes.

Finally, we supplemented the experiment as requested by the reviewers.

Firstly, the supplementary experiment of the charge density is performed during the fluctuation simulation test. And the charge density of Pem-iTENG with bionic cilia unmodified or modified TiO_2/PANI is tested before and after the illumination (see Fig. s1.2). The metal copper is the fixed electrodes (see Fig.s1.2a), and the output end of the electrode is connected to the test equipment through a rectifier bridge. The surface structure of single cilia indicted that the surface of bionic cilia is wrapped with a layer of modified TiO_2/PANI (see Fig.s1.2b).

The charge density of Pem-iTENG with bionic cilia unmodified TiO_2/PANI

remains at 122.38 nC in the dark and light (see Fig.s1.2c). This indicates that sunlight cannot enhance the charge density of Pem-iTENG after the illumination. The charge density of Pem-iTENG with bionic cilia modified TiO_2/PANI increases from 122.73 nC to 164.12 nC in the dark and light (see Fig. s1.2d), and it reveals that after modified with TiO_2/PANI, sunlight can enhance the charge density of Pem-iTENG. It is worth noting that compared with unmodified TiO_2/PANI in dark, the charge density of Pem-iTENG modified TiO_2/PANI in the dark is very close. After eliminating the influence of sunlight, it indicates that factors of surface roughness, etc. have basically no effect on the charge density before and after modification. Therefore, we demonstrate that sunlight can excite a significant change in the charge density of our Pem-iTENG modified TiO_2/PANI during the fluctuation simulation test.

Secondly, we undergo the light effect experiments (open circuit voltage and short circuit current) during the process of light-dark-light state (Fig.s1.3). As a control group, light effect experiments of Pem-ITENG with PDMS film are carried out (Fig.s1.3a,b). The frequency of the reciprocating motion of the driving motor is set to 2 Hz. The open circuit voltage (about 15V) and short circuit current (700μA) of Pem-iTENG without bionic cilia unmodified TiO_2/PANI are not significantly changed in the light-dark-light state (see Fig.s1.3 a,b). Hence, it indicates that the increase of electron transfer is induced by photon excitation effect of the insulator surfaces can be eliminated.

After the surface covered with bionic cilia modified TiO_2/PANI, the open circuit voltage and short circuit current of Pem-iTENG modified TiO_2/PANI are significantly improved in light compared with in dark. The open circuit voltage increases from 38.8 V in dark to 59.87 V in light, and the short circuit current increases from 2.4 mA in dark to 4.5 mA in light (see Fig.s1.3c,d). This indicates the enhancement of light effect experiments of Pem-iTENG is attributed to the photocatalysis. Therefore, compared with the improvement of the light effect experiments of Pem-iTENG with bionic cilia modified TiO_2/PANI in dark and light, we confirmed that the increase of electron transfer is induced by photocatalysis after excluding the light excitation.

Thirdly, through magnetic self-assembly technology, the solid-liquid contact angle of the PDMS film with bionic cilia is 165.28 ° (see Fig.s1.4 c), while bionic cilia modified TiO_2/PANI is 162.04 ° (see Fig.s1.4d). It indicates that the PDMS films with bionic cilia modified or unmodified TiO_2/PANI have certain super-hydrophobic properties, and the modification of TiO_2/PANI has no significant effect on the

solid-liquid contact angle. Moreover, PDMS film modified TiO_2/PANI with a light irradiation result shows that the solid-liquid contact angle after the light illumination of 1h is 159.74°, and the solid-liquid contact angle after the light illumination of 3h is 162.13°. Comparing the contact angles of bionic cilia modified or unmodified TiO_2/PANI, it shows that the light irradiation time has no significant effect on the solid-liquid contact angle. Therefore, we can exclude the influence of the contact angle on the solid-liquid surfaces leading to the increase of electron transfer.

In summary, we have verified that a significant enhancement in the charge density of Pem-iTENG modified TiO_2/PANI is illuminated by sunlight during the fluctuation simulation test. Then, we have confirmed that the increase of electron transfer is induced by photocatalysis instead of photon excitation effect. Finally, the influence of the contact angle on the solid-liquid surfaces leading to the increase of electron transfer is excluded. Therefore, we demonstrate that through systematic experiments, our hypothesis for the photoelectric-mechanical integrated triboelectric nano-generator is correct, and the working mechanism of the proposed Pem-iTENG is fully convincing.

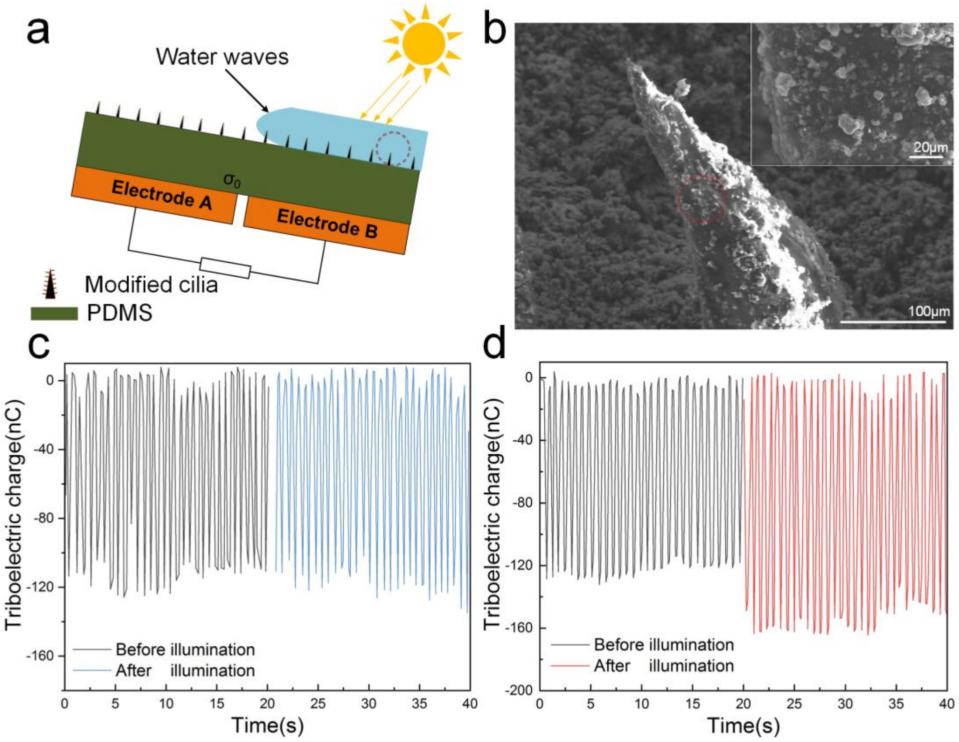

Fig.s1.2 Charge density diagram under different experimental conditions: (a) simple structure diagram of Pem-iTENG, (b) single bionic cilia modified TiO_2/PANI, (c) charge density unmodified TiO_2/PANI before and after the illumination, (d) charge density modified TiO_2/PANI before and after the illumination.

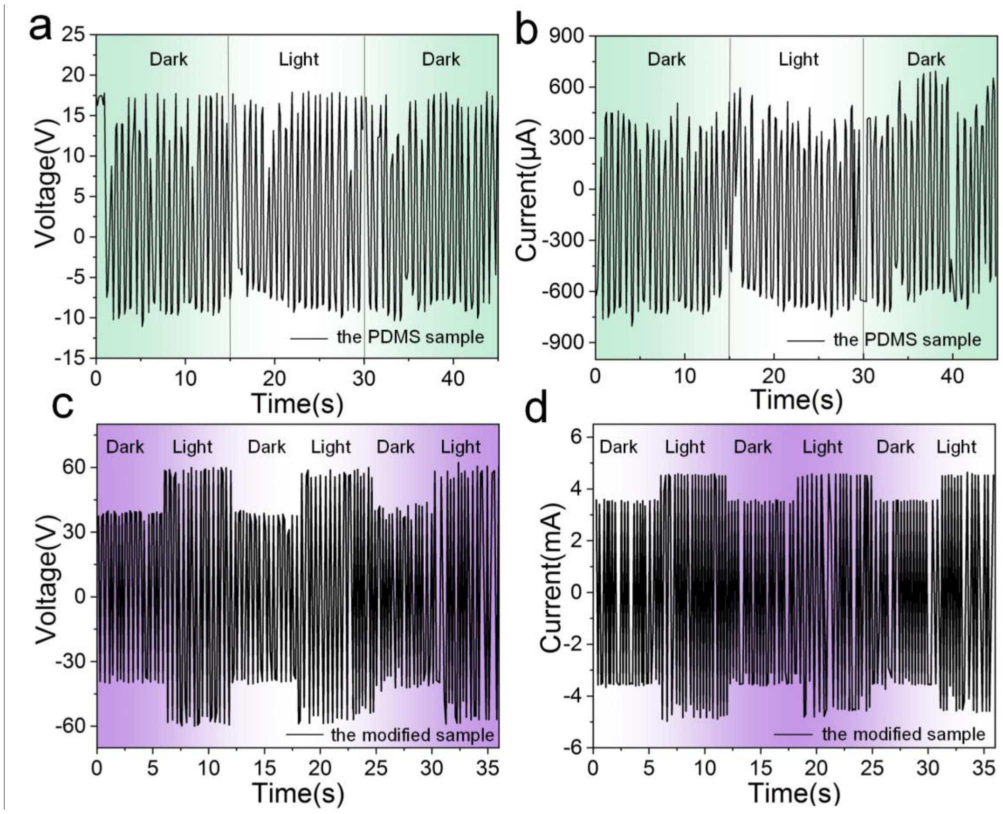

Fig.s1.3 the light effect experiments under light-darkness: (a) open circuit voltage and short circuit current (b) of PDMS without bionic cilia unmodified TiO_2/PANI, (c) open circuit voltage and short circuit current (d) of PDMS with bionic cilia modified TiO_2/PANI.

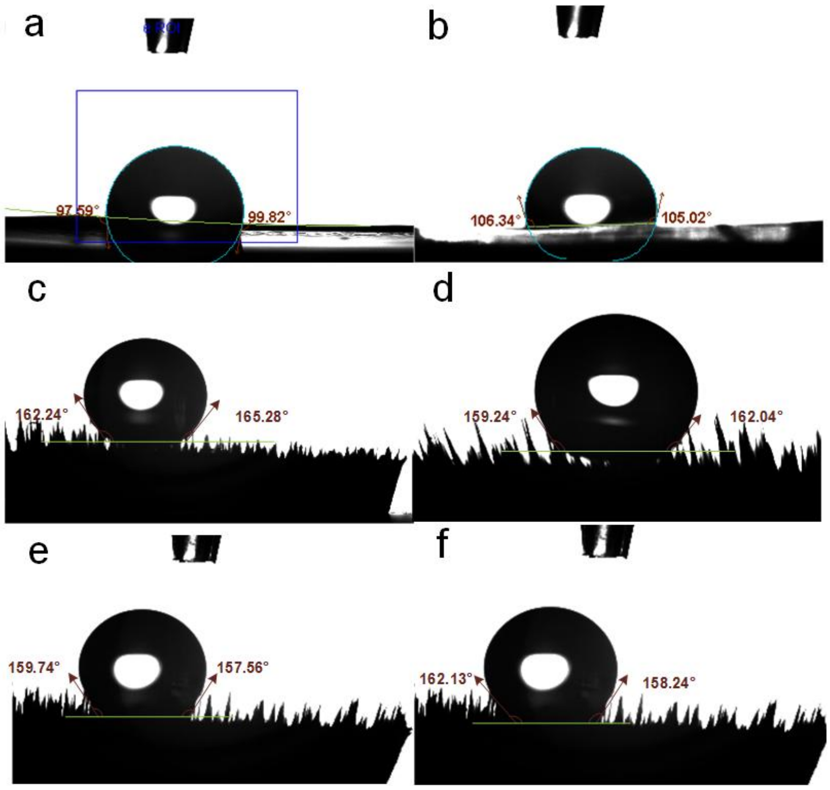

Fig. s1.4 Solid liquid contact angle of different samples: (a) the contact angle of pure PDMS, (b)

the contact angle of the PDMS doped with cobalt powder, (c) the contact angle of PDMS with bionic cilia unmodified TiO_2/PANI, (d) the contact angle of PDMS with bionic cilia modified TiO_2/PANI, (e) the contact angle of PDMS with bionic cilia modified TiO_2/PANI after illumination for 1 h, (f) the contact angle of PDMS with bionic cilia modified TiO_2/PANI after illumination for 3 h.

4. The stability of the device should be considered, as TiO_2 is always regarded to accelerate degradation of polymer (PDMS in your device).

Response：Thank you for your suggestion. TiO_2 does accelerate the degradation of PDMS. The photo-generated electrons in the valence band are excited to the conduction band, leaving a positively charged hole. The photo-generated holes then lead to the formation of free radicals. These free radicals have strong oxidizing properties and can break the chemical bonds of organic molecules. At the same time, the photo-generated electron-hole pairs generate water molecules and hydroxyl groups on the surface under ultraviolet radiation, and these activated molecules partially leave the siloxane bonds of the surrounding PDMS molecules. However, due to the excellent stabilities of the PDMS film in thermal, chemical and photocatalytic degradation, the degradation rate of PDMS by TiO_2 is not too fast, as reported in the literature (Advanced Materials 29.16(2017):1604637).

At the same time, when the critical mass of PDMS is about 30-40 wt%, it can form a thick enough polymer layer, which maintains a rough surface morphology, and obtains the largest water contact angle, inhibit photodegradation. This result is consistent with the critical mass of 30 wt% of PDMS’s reported by Qing et al. (Colloids and Surfaces A, 2015, 484: 471-477). The interaction between TiO_2 and PDMS is the hydrogen bonding between the hydroxyl groups adsorbed on the surface of TiO_2 and the oxygen atoms of the PDMS backbone. Therefore, through a wide range of intermolecular interactions, high surface coverage, excellent main chain flexibility, and optimal ratio, PDMS can inhibit the photo-degradation caused by TiO_2 particles. Additionally, many methods can inhibit or reduce photo-degradation. For example, the formulations of adding CeO_2 nanoparticles forming super-hydrophobic (Molecular Systems Design & Engineering, 2020, 5(4): 876-881).

Therefore, the problem of TiO_2 accelerated the degradation of the polymer (PDMS in the device) can be solved.

5. Why the changes of OV and SA are out of sync, wherein the biggest OV appears when frequency of fluctuation is 1 Hz and SA at 2 Hz.

Response: Based on previous studies, the open-circuit voltage and short-circuit current of solid-liquid contact triboelectric nano-generator are not necessarily linear. For example, Ahmed, et al. designed a fully enclosed duck-shaped TENG that can effectively collect wave energy from random and low-frequency water (Adv. Energy Mater. 6 (2016) 1601705). The open-circuit voltage in 1.95 Hz is lower than that in 2.50 Hz, but the short-circuit current does not change significantly under the two frequencies. From the perspective of traditional electricity, the output voltage is the largest at a certain frequency, and then, the output current must also be the largest at this moment. However, Pem-iTENG has a novel working mechanism in the complex and changeable test environment, which is somewhat different in the traditional cognition.

There are several reasons that may explain this phenomenon. From the perspective of the test method, the electrical performance of our Pem-iTENG is not measured at the same time but separately tested. From the perspective of working conditions, the frequency of flooding the photoelectric-electromechanical integrated composite membrane (Pem-iCM) is different in water fluctuation frequencies, and the number of photo-generated electron-hole pairs separated under illumination is also different, so the efficiency of light energy and mechanical energy conversion to electrical energy is not necessarily the same at different frequencies. Besides, it is difficult for the Pem-iTENG to fully exert its excellent photoelectric conversion characteristics when completely submerged in water, and it also has some challenges to avoid various changes introduced in the modification.

Therefore, the open-circuit voltage reaches the peak at the frequency of the water wave at 1 Hz, and the short-circuit current reaches the maximum at the water fluctuation frequency at 2 Hz.

Reviewer #2:

A bio-inspired liquid-solid photoelectric electromechanical integrated triboelectric nano-generator (Pem-iTENG) is proposed. It is claimed that the electron transfer between water and the modified samples is enhanced by the photocatalysis. A new idea for improving the output performance of the TENGs is presented in this work. However, the experiments in this manuscript is not systematically carried out, and the working mechanism of the Pem-iTENG is not fully convincing (R2-F1). The biggest problem in this work is lack of the controlled experiments to verify the hypothesis. The only evidence is the enhanced output after illuminated by light. As we know that the contact electrification between liquid and solid is very sensitive to the contact angle of the solid surfaces. The modification of TiO_2/PANI, and light irradiation may change the contact angle of the solid surfaces and lead to the increase of electron transfer. Therefore, it is suggested that the contact angle of the PDMS without cilia, TIO_2/PANI modified PDMS with and without light irradiation should be measured to exclude the effect of contact angle on the electron transfe (R2-F2). Also, it is demonstrated that the photon excitation effect of the insulator surfaces may lead to change of the electron transfer between two contact materials. In order to confirm that the increase of electron transfer is induced by photocatalysis instead of photon excitation effect, the light effect experiments (Fig. 3a, 3b) of the unmodified sample surfaces should be tested as a control group (R2-F3). I think this manuscript cannot be published in current version. Following are my additional questions and concerns:

Response R2-F1: Thank you for your comments. We have demonstrated that a significant enhancement in the charge density of Pem-iTENG modified TiO_2/PANI is illuminated by sunlight during the fluctuation simulation test (details see Fig. s2.9 and R2-Q4). Then, we have confirmed that the increase of electrons transfer is induced by photocatalysis instead of photon excitation effect (details see Fig. s2.2 and R2-F2). Finally, we have excluded the influence of the contact angle on the solid-liquid surfaces leading to the increase of electrons transfer (details see Fig. s2.1 and R2-F3). Therefore, through systematic controlled experiments, we demonstrate that our hypothesis for the photoelectric-mechanical integrated triboelectric nano-generator is correct, and the working mechanism of the Pem-iTENG is fully convincing.

Response R2-F2: In order to exclude the influence of light irradiation and surface modification of TiO_2/PANI on the solid-liquid contact angle, we supplement the test of the solid-liquid contact angle in the following six samples, as shown in Fig. s2.1.

Through magnetic self-assembly technology, the solid-liquid contact angle of the PDMS film with bionic cilia is 165.28°(see Fig.s2.1(c)), while bionic cilia e modified TiO_2/PANI is 162.04°(see Fig.s2.1(d)). It indicted that the PDMS films with bionic cilia modified or unmodified TiO_2/PANI have certain super-hydrophobic properties, and the modification of TiO_2/PANI has no significant effect on the solid-liquid contact angle.

Moreover, PDMS film modified TiO_2/PANI with a light irradiation results show that the solid-liquid contact angle after the light illumination of 1 h is 159.74°, and the solid-liquid contact angle after the light illumination of 3 h is 162.13°. Comparing the contact angles of bionic cilia modified or unmodified TiO_2/PANI, it shows that the light irradiation time has no significant effect on the solid-liquid contact angle.

Therefore, we can exclude the influence of the contact angle on the solid-liquid surfaces leading to the increase of electron transfer.

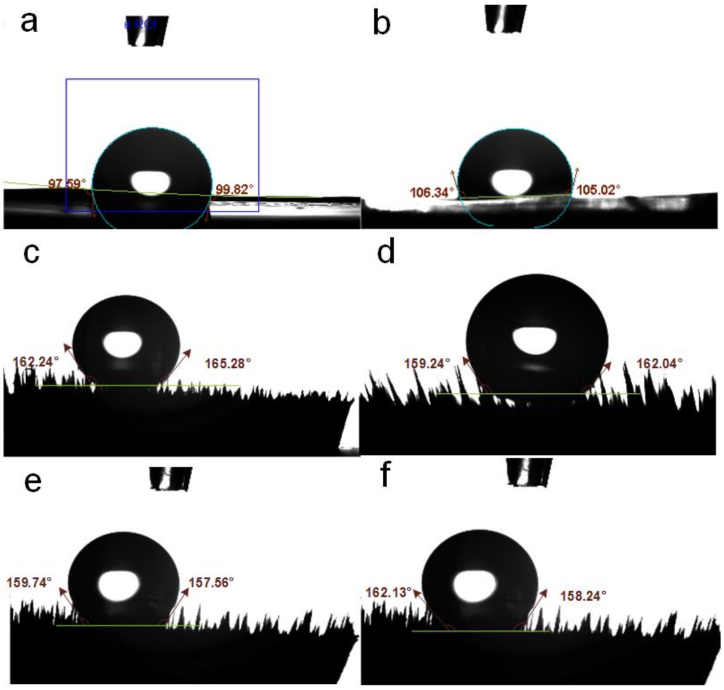

Fig. s2.1 Solid-liquid contact angle of different samples: (a) the contact angle of pure PDMS, (b) the contact angle of the PDMS doped with cobalt powder, (c) the contact angle of PDMS with bionic cilia unmodified TiO_2/PANI, (d) the contact angle of PDMS with bionic cilia modified TiO_2/PANI, (e) the contact angle of PDMS with bionic cilia modified TiO_2/PANI after illumination for 1 h, (f) the contact angle of PDMS with bionic cilia modified TiO_2/PANI after illumination for 3 h.

Response R2-F3: In order to confirm that the increase of electron transfer is induced by photocatalysis instead of photon excitation effect, we undergo the light effect experiments (open circuit voltage and short circuit current) during the process of light-dark-light state (Fig.s2.2). As a control group, light effect experiments of Pem-ITENG with PDMS film are carried out (Fig.s2.2a,b). The frequency of the reciprocating motion of the driving motor is set to 2 Hz. The open circuit voltage (about 15V) and short circuit current (700μA) of Pem-iTENG without bionic cilia unmodified TiO_2/PANI are not significantly changed in the light-dark-light state (see Fig.s2.2 a,b). Hence, it indicated that the increase of electron transfer is induced by photon excitation effect of the insulator surfaces can be eliminated.

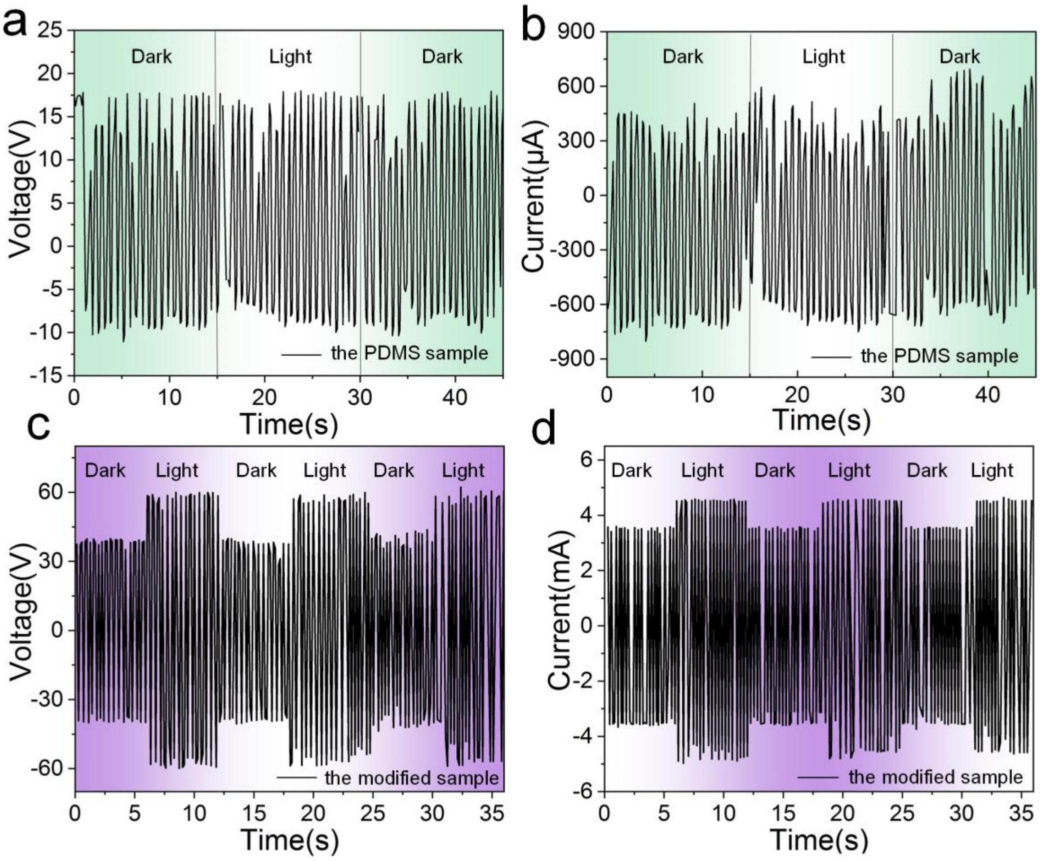

Fig.s2.2 The light effect experiments under light-darknes: (a) open circuit voltage and short circuit current (b) of PDMS without bionic cilia unmodified TiO_2/PANI, (c) open circuit voltage and short circuit current (d) of PDMS with bionic cilia modified TiO_2/PANI.

After the surface covered with bionic cilia modified TiO_2/PANI, the open circuit voltage and short circuit current of Pem-iTENG modified TiO_2/PANI are significantly improved in light compared with in dark. The open circuit voltage increases from 38.8 V in dark to 59.87 V in light, and the short circuit current increases from 2.4 mA in dark to 4.5 mA in light (see Fig.s2.2c,d). This indicated the enhancement of light

effect experiments of Pem-iTENG is attributed to the photocatalysis.

Therefore, compared with the improvement of the light effect experiments of Pem-iTENG with bionic cilia modified TiO_2/PANI in dark and light, we confirmed the increase of electron transfer is induced by photocatalysis after excluding the light excitation.

1. In the photocatalytic system (Fig. 1b), the authors claim that the holes tend to be retained in the VB of the TiO_2, while the electrons will transfer to cilia root. There are no evidences to support this explanation, because there is no field to separate the electron from the holes. Why the holes react with the water molecules and the impurities in liquid water, and the electrons don't?

Response: Thanks for your comments. Since our description of the separation of photogenerated electrons and holes in the earlier draft is relatively rough, we explain the separation of photogenerated electrons and holes in detail here.

When TiO_2 is modified by PANI, a type-II P-N heterojunction is formed at the interface of the TiO_2/PANI. Under the illumination of sunlight, TiO_2 and PANI primarily absorb UV and visible portions of the incident light, respectively. The photogenerated electrons and holes are separated by the built-in field (Advanced Energy Materials, 2020, 10(9): 1903713. ACS Applied Energy Materials, 2018, 1(5): 2087-2095. Small, 2016, 12(42): 5809-5816. ACS Applied Energy Materials, 2018, 1(5): 2087-2095.), and they move into TiO_2 (electrons) and PANI (holes), respectively (Fig.s2.3a,b).

When the Pem-iTENG is placed in a marine environment, the PANI covered at the surface of bionic cilia is exposed to moist water mist or organic matter, etc (ACS Nano, 2020, 14 (3): 3630-3639). The holes (h^+) at surface of the PANI will react with water molecules to form hydroxyl radical (Fig. s2.3(c)ii), which contributes to the oxidation and degradation of organic molecules (see Equation 2.1-2.4), while the excess photoelectrons will exist in TiO_2 due to the TiO_2 particles being wrapped between PANI and the bionic cilia (Fig. s2.3(c)i). Here, we believe that a few photoelectrons retained in PANI can react with oxygen to produce superoxide radicals, but the retained photoelectrons in PANI are very few, which can be ignored.

Therefore, through the built-in electric field of the P-N heterojunction, the effective separation of photogenerated electrons and holes can be realized.

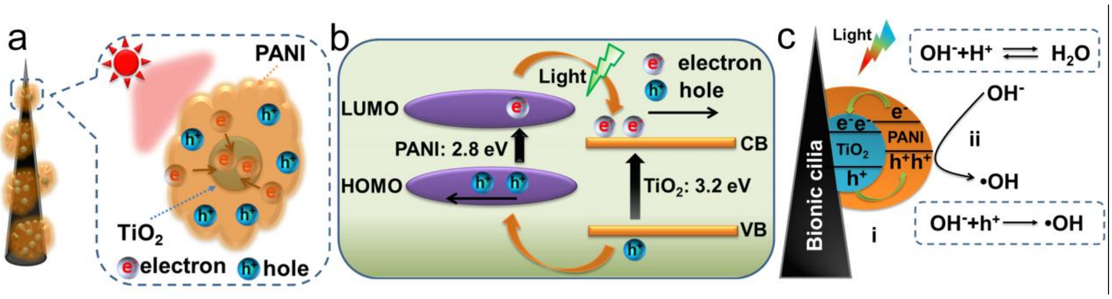

Fig. s2.3 Separation mechanism of photogenerated electrons and holes: a energy level conversion ofP-N heterojunction b schematic diagram of movement of photoelectrons and holes in the built-in field of P-N heterojunction. c charge transfer in the photocatalysis on single cilia surface.

$$\text{PANI/TiO}_2 + hv \rightarrow \text{PANI}^+ / \text{TiO}_2 + e^-_{CB} \tag{2.1}$$

$$e^-_{CB} + O_2 \rightarrow O_2^- \tag{2.2}$$

$$\text{PANI}^+/\text{TiO}_2 \rightarrow \text{PANI/TiO}_2 + h^+_{VB} \tag{2.3}$$

$$h^+_{VB} + (H_2O \Leftrightarrow H^+ + OH^-) \rightarrow H^+ + OH^- \tag{2.4}$$

2. I think the working principle of the Pem-iTENG is incorrect in Fig. 1d. Why the water is positively charged in the schematic? Here are two references for authors (Adv. Mater. 26 (2014) 4690; Nano Energy, 36 (2017), 250-259; Adv. Mater. Interfaces, 6 (2019), 1901547.), in which the principle of the liquid-solid TENG is described clearly and correctly.

Response: Thanks for your comments. The working principle of Pem-iTENG has been revised and explained as follows (Fig.1).

Specifically, a type II P-N heterojunction is formed at the interface of TiO_2 and PANI on the surface of bionic cilia. When the P-N heterojunction is illuminated by ultraciolet (UV) and visible portions of the incident sunlight, the effective separation of the photo-generated electrons (also called photoelectrons) and holes (Fig. 1b) can be realized by the built-in field of the P-N heterojunction (Advanced Energy Materials, 2020, 10(9): 1903713. ACS Applied Energy Materials, 2018, 1(5): 2087-2095. Small, 2016, 12(42): 5809-5816. ACS Applied Energy Materials, 2018, 1(5): 2087-2095.). Excess photoelectrons are retained in TiO_2 due to the TiO_2 particles being wrapped between PANI and bionic cilia. The photo-generated holes are transferred to PANI, then contact and react with moist seawater mist or organic impurities, etc. This causes the enhancement of the surface negative charge density of the PDMS film. Subsequently, through referring to the water wave motion generating the contact electrification (Nano Energy, 2019, 66: 104117. Advanced Energy Materials, 2017, 7(21): 1701243. Nano Energy, 2014, 9: 186-195.), we propose the working principle of Pem-iTENG under the synergy of the contact electrification and the photocatalysis

in a cycle of reciprocating tidal wave (see Fig. 1c).

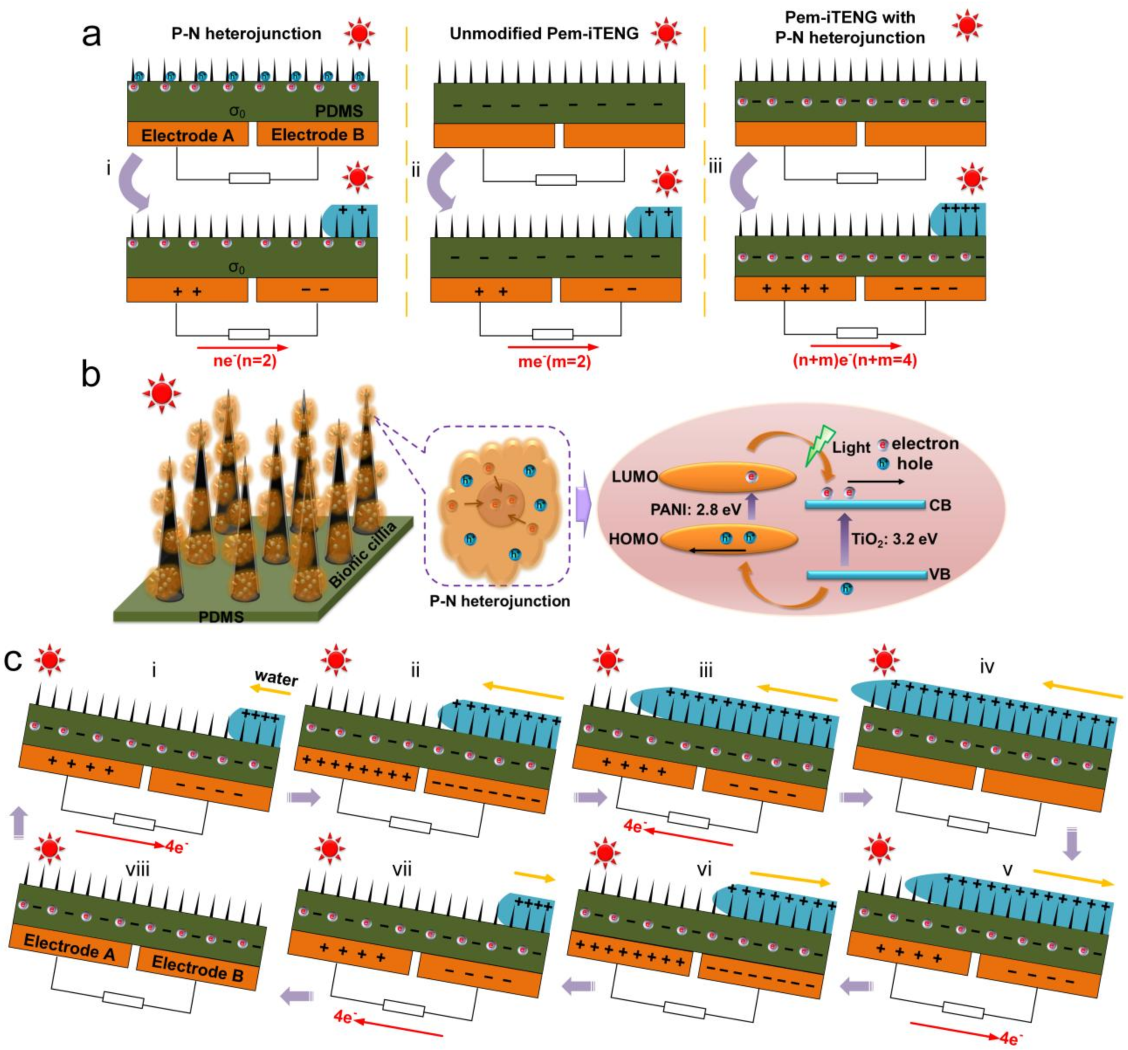

Fig. 1 the working principles of Pem-iTENG. (a) Pem-iTENG with P-N heterojunction (i), unmodified and modified Pem-iTENG during the two flow states of the tidal waves (ii and iii). (b) principles of photocatalysis of bionic cilia with P-N heterojunction. TiO_2 (bandgap 3.2 eV) can only absorb ultraviolet light, while PANI (bandgap 2.8 eV) can absorb both ultraviolet and visible light (c) the contact electrification of Pem-iTENG in a cycle of reciprocating tidal wave, and free electrons transfer direction between the two electrodes.

Once water waves immerse on the surface of the PDMS film with bionic cilia, the rising contact seawater forms an electric double layer with the PDMS film to screen the negative charges on the PDMS film partly. Due to the asymmetric distribution of charges, the unbalanced potential between the two electrodes drives free electrons to flow out from one electrode to the other, and forms the alternating current through the external circuit (see Fig. 1(c)i,iii,v,vii). When the tide wave rises or falls to the gap between the two electrodes, there is no electron transfer (see Fig. 1(c)ii,vi,). When the tidal wave rises to the top (see Fig. 1(c)iv), the potential difference between the two

electrodes decays to zero without electron transfer due to the symmetrical screening of the electric double layer. During the first rise of the water wave, the PDMS film keeps accumulating the triboelectric negative charges and photoelectrons without dissipating in a period time, and there is no electron transfer between the two electrodes (see Fig. 1(c)viii).

Based on the relevant information provided by the reviewers, we believe that the principle of the solid-liquid triboelectric nano-generator can be explained in two ways. One of the working model is based on the fact that liquid droplets constantly strike on the surface of the friction polymer film (Fig.s2.4), and the contact electrification phenomenon occurs (Nano Energy, 36 (2017), 250-259. Advanced Materials Interfaces, 2019, 6(24): 1901547.). Another solid-liquid contact model is that the water flow continuously washes the friction polymer film by the principle of electrostatic induction to collect electric charges (Fig.s2.5), and an alternating current is generated (Nano Energy (2014) 9, 186-195). In fact, the working principle of the power generation driven by liquid droplets or water waves is similar, and the output electrical performance is both realized by contact electrification. Our Pem-iTENG to realize the conversion of water wave energy to electric energy is more inclined to Yuanjie Su, et al. proposal of the collection charges by the electrostatic induction principle under the water flow (Fig.s2.5).

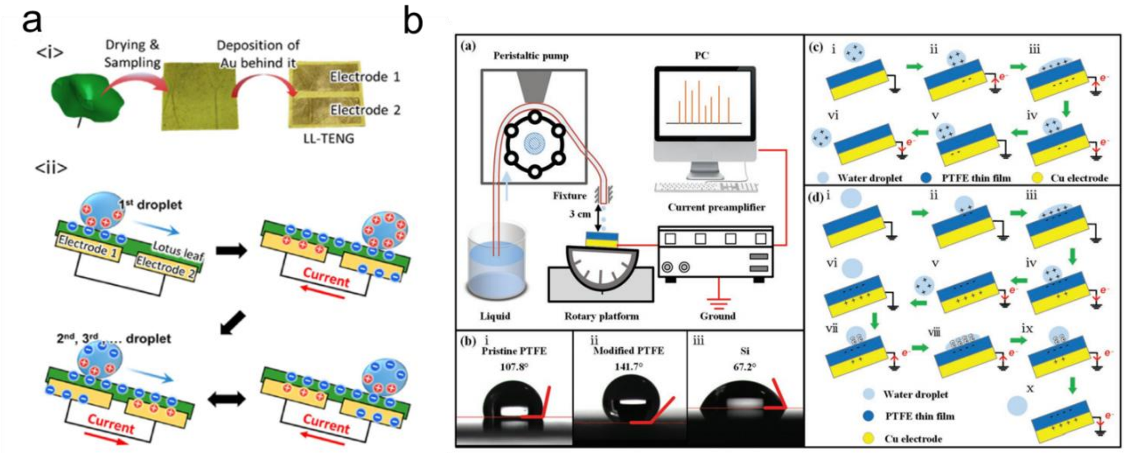

Fig. s2.4 schematic diagram of the natural lotus leaf-TENG proposed by Choi *et al* and Yang *et al*.

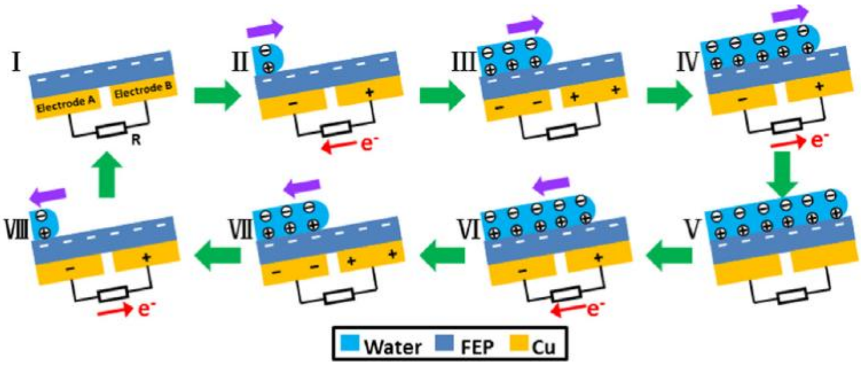

Fig. s2.5 Working principle of interfacial electrification enabled TENG by Yuanjie Su et al.

In addition, it is worth noting that seawater remains electrically neutral during the entire contact electrification. As known, the mechanism of triboelectrification is the formation of chemical bond at the contact position of two materials. Therefore, the formation of friction charge depends on the electrode difference of friction composite material itself. When two materials are in contact, the surface of the materials easy to

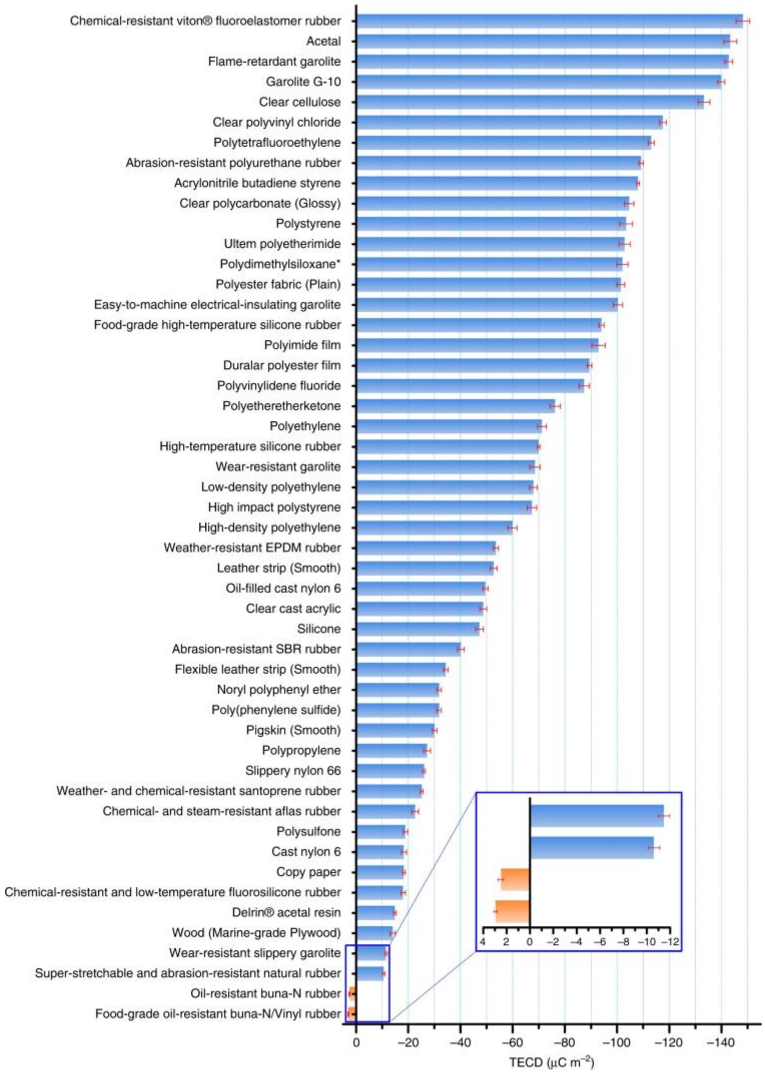

Fig. s2.6 The quantified triboelectric series (Nat. Commun. (2019) 10, 1427).

obtain electrons is negatively charged, while the surface of materials easy to lose electrons is positively charged (the electrostatic sequence table of common materials is shown in Fig. s2.6). In our research work, when the PDMS friction film contacts with seawater, due to the difference of the electron gain and loss ability between PDMS and seawater, the surface of PDMS is easy to obtain electrons and carries negative charges. These charges will not dissipate for a long time. Due to electrostatic induction, seawater carries positive charge. In fact, the electrical neutrality of seawater remains unchanged, but it shows a positive charge accumulation at the contact surface.

3. The Pem-iTENG is inspired by the lotus leaf, to obtain the advantages of hydrophobicity, self-cleaning and surface roughness. However, the effects of the hydrophilia and roughness of the TENG on the output performance are not discussed.

Response: Thanks for your comments. First of all, we focus on the photoelectric-mechanical integrated high-efficiency energy conversion technology in this work. In order to explain this problem, we analyze the characteristics of charge density on hydrophobic surfaces in Question 4. At the same time, it is necessary to say that the influence of hydrophobicity of solid-liquid triboelectric nano-generators on their performance has been reported as follows.

Lin *et al.* reported that in order to obtain the maximum electrical output, the previous water drop should be completely removed from the surface of the triboelectric nano-generators before the next drop falls on the triboelectric nano-generator, which requires super-hydrophobicity surface (Advanced Materials, 2014, 26(27):4690-4696). The current increases with the speed of water drop sliding. The super-hydrophobic property of triboelectric nano-generator also changes the surface water content. The hydrophilic nylon film and the untreated hydrophobic PTFE film with smooth surface were used to replace the original super-hydrophobic PTFE film, and the output current density decreased significantly (Fig. s2.7). This shows that the solid-liquid contact triboelectric nano-generator has the importance of super-hydrophobicity, and the hydrophobicity of the film is a key factor in the performance of the solid-liquid contact triboelectric nano-generator. In fact, we know that improving the surface roughness of friction film can improve the output

performance of solid-liquid contact triboelectric nano-generators, but the relationship between surface roughness and static contact angle is not simple linear. When the surface roughness exceeds a certain range, the surface roughness increases and the static contact angle of the materials decreases, which makes the material surface lose the super-hydrophobic property. This is contrary to the design and fabrication of super-hydrophobic triboelectric nano-generator with solid-liquid contact.

Zhou *et al.* proved that the super-hydrophobic surface can improve the stability of TENG (Nano Energy, 2019, 57: 903-910). Fig. s2.8a shows that with the increase of RH, the output voltage of triboelectric nano-generators decreases. However, when the relative humidity is as high as 80%, the output voltage of super-hydrophobic PDMS's TENG is much higher than that of ordinary PDMS. When TENG is wetted by water, the recovery time of its output performance (see Fig. 2.8b and 2.8c) shows that it takes 66.2 s for TENG of ordinary PDMS to recover its output performance from water wetting, while only 24.6s is needed for super-hydrophobic PDMS's TENG under the same conditions. In addition, due to the self-cleaning characteristics of super-hydrophobic surface, the dust on the TENG can be easily washed off to keep the surface clean, thus prolonging the service life of TENG.

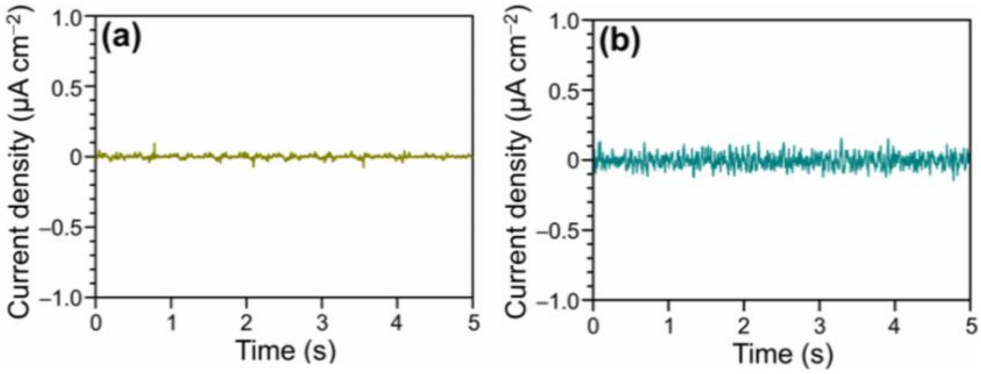

Fig. s2.7 Output current density of water-TENG composed of hydrophilic material nylonfilm (a) and hydrophobic PTFE film (b) with smooth surface

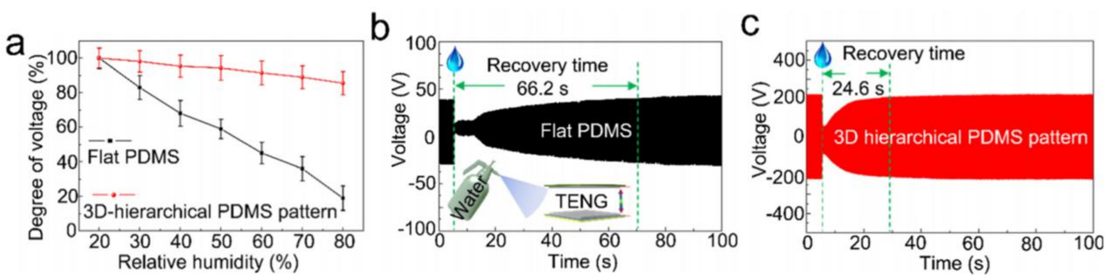

Fig.s2.8 (a) RH effects upon the output voltage of the TENGs with a flat PDMS interlayer and a 3D hierarchical PDMS interlayer. (b) recovery time of the output signal after the TENGs with a flat PDMS interlayer, (c) 3D hierarchical PDMS interlayer dampened by spraying of water twice.

Secondly, the effect of surface roughness difference on the output performance of solid-liquid contact triboelectric nano-generators has been proposed in this paper, but we define it as the cilia's aspect ratio of photoelectric electromechanical integrated composite membrane (PEM-iCM). Moreover, the surface roughness is basically unchanged in the same length of bionic cilia modified or unmodified TiO2/PANI. The effect of the surface roughness of bionic cilia formed by the TiO2/PANI is excluded during the seawater reciprocating scouring (see Fig.s2.1 and Fig.s2.9). The electrical properties of the composite friction film prepared by magnetic adsorption of three kinds of cobalt powder (300nm, 800nm, 1300nm) were tested in the text of the manuscript. The results show that the sample with the particle size of 800 nm has the best electrical properties, so this surface roughness of the sample is the most suitable for solid-liquid contact triboelectric nano-generator.

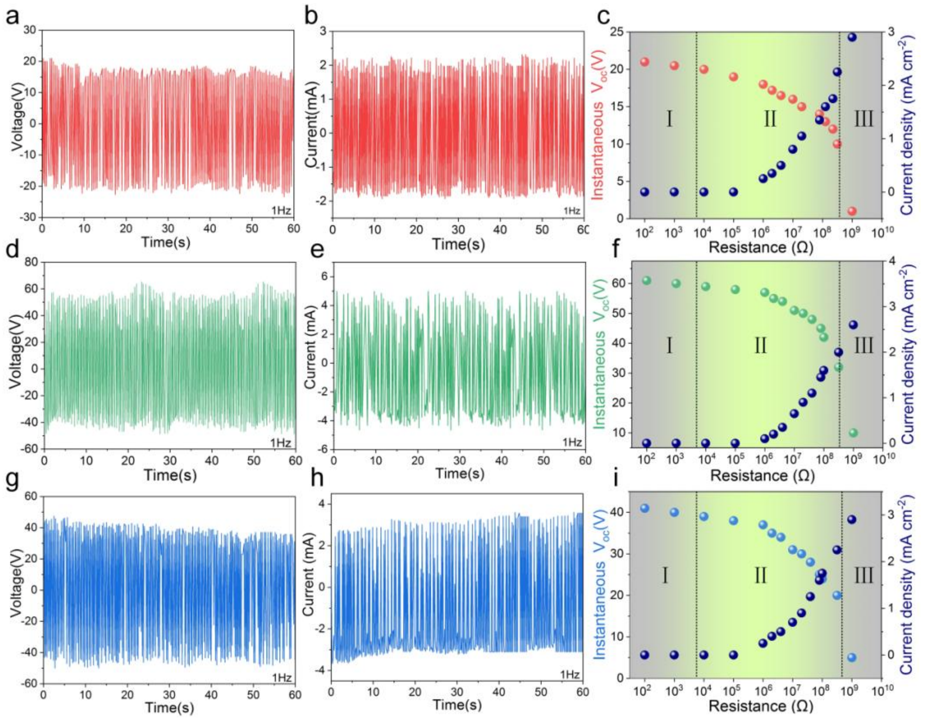

Fig. 4 a, d, g are the open circuit voltage at 2 Hz solid-liquid contact frequency of three kinds of high width ratio fiber wool samples; **b, e, h** are the short-circuit current of three kinds of high width ratio fiber samples with solid-liquid contact frequency of 2 Hz; **c, f, i** are the change diagram of output voltage and output current of solid-liquid contact Pem-iTENG with external resistance between 10^2 Ω and 10^9 Ω.

In order to improve the surface charge density, the method of changing the particle size of cobalt powder (300nm, 800nm, 1300 nm) was used to prepare PEM-iCM samples with various aspect ratio of cilia, so as to improve the surface contact electrification state. Fig. 4 shows the electrical properties of Pem-iTENG samples

with three aspect ratios at the water wave frequency of 2 Hz. The results show that the output voltage of triboelectric nano-generator can be increased by the microstructure of 300 nm, 800 nm and 1300 nm. The self-growing bionic cilia of 800 nm magnetic cobalt powder can improve the output voltage most obviously. The maximum open circuit voltage is 64.2 V (see Fig.4d) and the maximum short-circuit current is about 5.1 mA (see Fig.4e), which is much higher than the previous solid-liquid contact TENGs such as linear, cubic, pyramid and other microstructures.

4. It is suggested that the surface charge density of the samples in the experiments should be measured or calculated.

Response: The supplementary experiment of the charge density is performed during the fluctuation simulation test. And the charge density of Pem-iTENG with bionic cilia unmodified or modified TiO_2/PANI is tested before and after the illumination (see Fig. s2.9). The metal copper is the fixed electrodes (see Fig.s2.9a), and the output end of the electrode is connected to the test equipment through a rectifier bridge. The surface structure of single cilia indicted that the surface of bionic cilia is wrapped with a layer of modified TiO_2/PANI (see Fig.s2.9b).

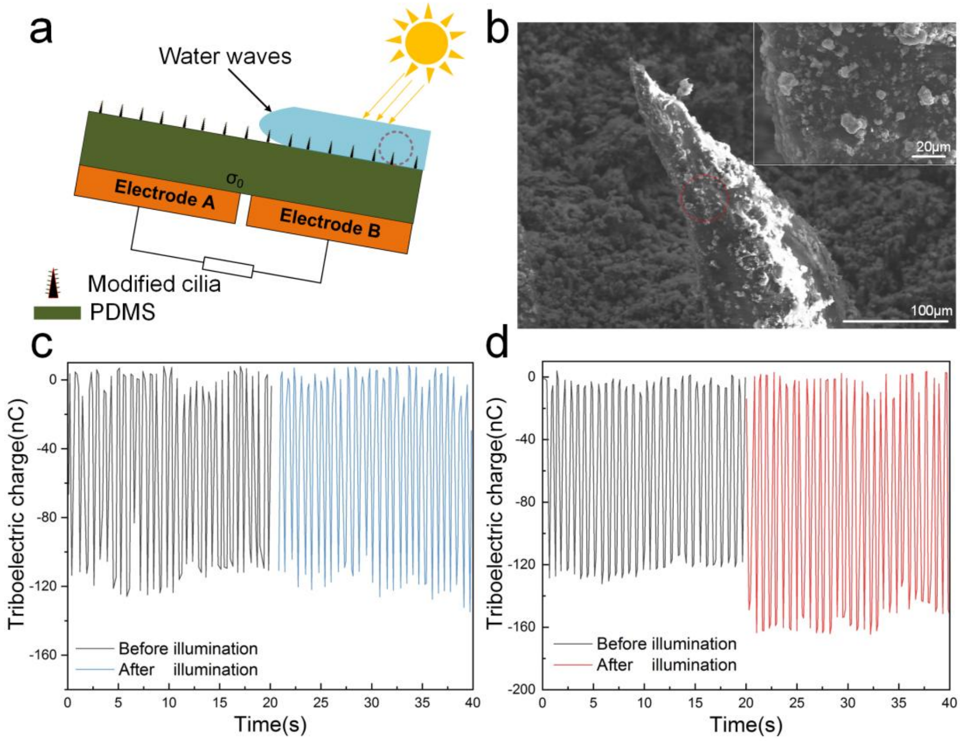

Fig.s2.9 Charge density diagram under different experimental conditions: (a) simple structure diagram of Pem-iTENG, (b) single bionic cilia modified TiO_2/PANI, (c) charge density

unmodified TiO_2/PANI before and after the illumination, (d) charge density modified TiO_2/PANI before and after the illumination.

The charge density of Pem-iTENG with bionic cilia unmodified TiO_2/PANI remains at 122.38 nC in dark and light (see Fig.s2.9c). This indicated that sunlight cannot enhance the charge density of Pem-iTENG after the illumination. The charge density of Pem-iTENG with bionic cilia modified TiO_2/PANI increases from 122.73 nC to 164.12 nC in the dark and light (see Fig. s2.9d), and it revealed that after modified with TiO_2/PANI, sunlight can enhance the charge density of Pem-iTENG. It is worth noting that compared with unmodified TiO_2/PANI in dark, the charge density of Pem-iTENG modified TiO_2/PANI in dark is very close. After eliminating the influence of sunlight and the surface hydrophobic properties (Fig.s2.1), it indicated that surface roughness has basically no effect on the charge density before and after modification.

Therefore, through the systematic experimental test results of charge density as well as excluding the increase of electron transfer is induced by photocatalysis instead of photon excitation effect (see Fig.s2.2), we demonstrate that the working mechanism of the proposed Pem-iTENG are correct, and our Pem-iTENG can achieve the photoelectric-mechanical integrated high-efficiency energy conversion.

5. The academic term for “friction electrification” is “triboelectrification” or “contact electrification”.

Response: Thanks for your comments. We have carefully checked the differences and made corrections, which is highlighted in red.

Reviewer #3:

The authors developed a bio-inspired solid-liquid interface contact-based photoelectric-electromechanical integrated triboelectric nano-generator. The energy conversion efficiency can reach 67.4%. The authors contributed this high efficiency to the efficient sunlight capture by PN heterojunction. The topic is interesting, but it seems that there are many experimental results/proposed mechanism which needs carefully being addressed before publication.

1. The diagrams of energy level alignment between PANI and TiO_2 in Figure 1b and c are contradictory. In figure 1b, the interface seems like a type Ⅰ heterojuction. But in the figure 1c, the energy alignment of the heterojunction is type Ⅱ. Additionally, I not sure that it is suitable to descript this interface by P-N junction? Polyanine is conducting polymer instead of semiconductor one. And in figure 1c, the bandgap values between PANI and TiO_2 are different. But in the picture the distances between CB and VB of two different materials are almost the same. It is contradictory.

Response: Thanks for your comments. We carefully checked the problem of the contradiction of the energy level arrangement between PANI and TiO_2 (see revised Fig. 1(a)), and the band gap size of PANI has been corrected in the manuscript.

For the problem of P-N junction, we know that the interface heterojunction can be classified into three types of type-I, type-II and type-III, while Z-type photoreaction system includes the photoreaction system i and the photoreaction system ii (Chemical Communications, 2001, 23 (1): 2416-2427). In the process of Z-type catalytic reaction, photogenerated electrons (also called photo electrons) and holes are generated after photoexcitation for the photoreaction system i and ii. Electrons in photoreaction system ii are oxidized by oxidation-reduction couples, while electrons in photoreaction system i are reduced by oxidation-reduction couples. In this way, more photoelectrons on the conduction band of photoreaction system i and holes on the valence band of photoreaction system ii can be reserved (see Fig. 3.1). Therefore, the symbol marks in the article made the reviewers misunderstand. Actually, the interface formed by PANI/ TiO_2 described in Figs. 1(b,c) (see revised Fig. 1(a)) in this paper is the same type, that is, the interface of P-N heterojunction is type-II heterojunction. In

order to make it clear, we have modified the symbols in the article.

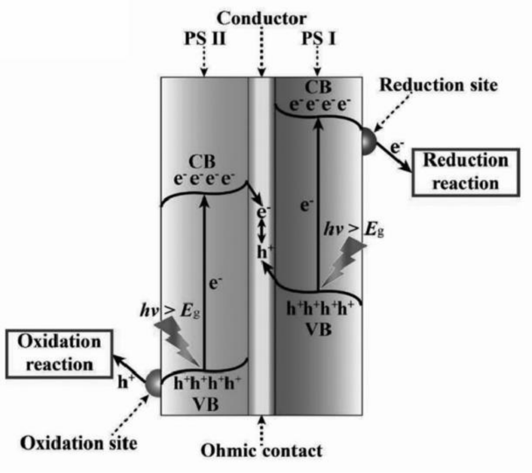

Fig. s3.1 PS-C-PS type Z photocatalytic reaction mechanism.

In view of this interface by P-N junction proposed by the reviewers, the heterojunction interface formed by semiconductor of TiO_2 and conductive polymerization of PANI on cilia surface in this paper can be called P-N heterojunction, but it is different from the traditional semiconductor heterojunction. **The detailed explanation about P-N heterojunction is as follows.**

Firstly, many N-type oxides with semiconductors characteristics have been proposed, such as titanium dioxide, tin oxide, zinc oxide and other semiconductor metal oxides. However, few metal oxides with good visible/ ultraviolet (UV) photosensitivity have been reported. There are few kinds of nanomaterials with photosensitivity. Even if some nanomaterials show photosensitivity, there is still much room for improvement in their photosensitivity. After modifying the surface of metal oxide with conductive polymerization, the photosensitivity of these nanomaterials can be improved, or the sensitive range to light wave can be widened, which make the photosensitivity enhance to some extent. For example, Mojtabavi et al. developed a flexible self-powered photodetectors (PDs) with multi-color detection capability (Applied Surface Science, 2019, 492: 189-198). Polyaniline/Titanium dioxide (PTP) and Titanium dioxide/Polyaniline (TPP) heterojunctions were deposited on a flexible PET substrate. Under UV and visible light irradiation, the I-V curves of PTP and TPP show different contrast ratios, rectification ratios, effective barriers height and reverse saturation currents, which indicates that the device can be used as an effective two-color PDs without an external power source. At the same time, Zhonglin Wang's team made a zinc oxide nano-sensor, whose surface was modified by sodium polystyrene sulfonate (PSS), and experiments showed that the absorption capacity of

the sensor for ultraviolet light was greatly enhanced, and the photosensitivity was increased by five orders of magnitude (Journal of the American Chemical Society 129.40 (2007): 12096-12097).

Secondly, conductive polymerization is widely used in low-cost photosensitive devices, mainly because their internal electrons exist in the form of ring π, so they have strong light absorption ability. Metal oxides are often modified by conductive polymerization with some excellent properties. Taking titanium dioxide (TiO_2) as an example, conductive polymerization can broaden the photosensitive wavelength range of TiO_2, improve the photosensitivity, and accelerate the separation rate of photogenerated electrons and photogenerated holes. Many conductive polymerizations can be used to modify semiconductor metal oxides, such as sodium polystyrene sulfonate (PSS), poly dimethyl diallyl ammonium chloride (PDADMAC or PDDA), polyaniline (PANI), poly pyrrole (PPY) and some dyes. For example, F. F. Amos et al. fabricated photosensitive detectors by adsorbing cadmium sulfide semiconductor nano-arrays on elastic PET substrates in aqueous solution. They created another membrane with composite structure, which was prepared by using LBL technology to adsorb titanium dioxide nanosheets, PEI and PDDA on conductive ITO substrate (Superlattices and Microstructures, 2010, 48 (2): 170-180). As we all know, ions in semiconductor metal oxides are stable and immobile, so the conduction between ions can be neglected. These ions are in contact with each other between the N-type semiconductor and the P-type conductive polymerization, and the interface contact area between them forms a depletion area composed of charged ions, which is the P-N junction, as shown in Fig.s3.2.

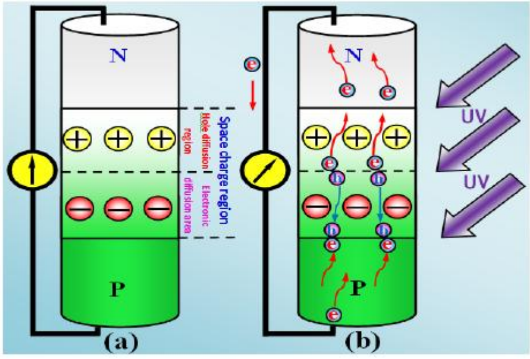

Figure s3.2 Schematic diagram of the working mechanism of P-N heterojunction.

Therefore, the heterojunction interface formed by semiconductor of TiO_2 and conductive polymerization of PANI on cilia surface in this paper can be called P-N heterojunction, but it is different from the traditional semiconductor heterojunction.

The traditional semiconductor heterojunction is a special kind of P-N junction, which is formed by depositing more than two layers of different semiconductor materials on the same pedestal in turn with different energy band gaps.

For the problem of the bandgap values between PANI and TiO_2 in Fig. 1c (the distances between conduction band (CB) and valence band (VB) of two different materials are almost the same), we have revised the distances between CB and VB in the full paper (see revised Fig. 1(a)).

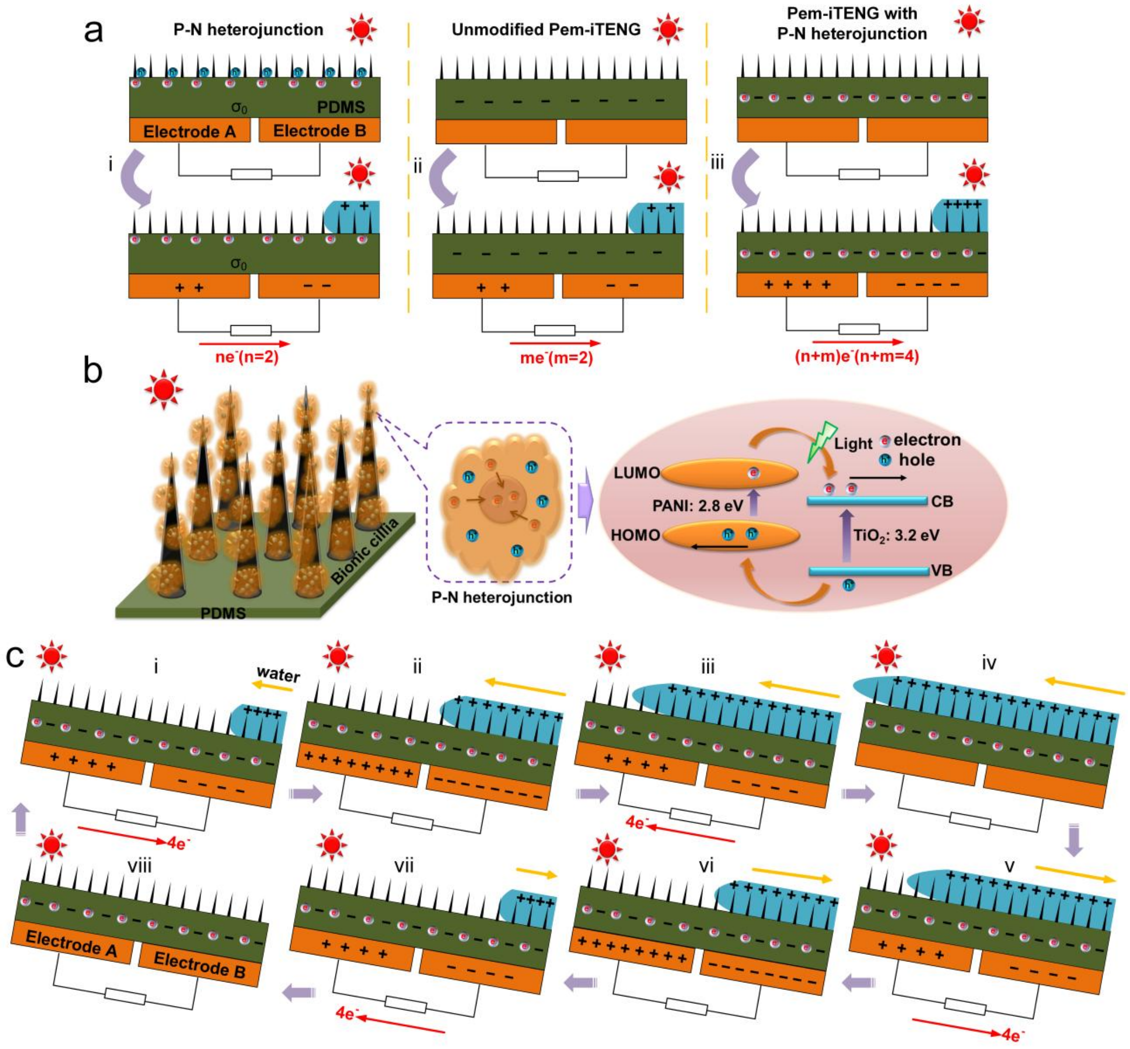

Fig. 1 the working principles of Pem-iTENG. (a) Pem-iTENG with P-N heterojunction (i), unmodified and modified Pem-iTENG during the two flow states of the tidal waves (ii and iii). (b) principles of photocatalysis of bionic cilia with P-N heterojunction. TiO_2 (bandgap 3.2 eV) can only absorb ultraviolet light, while PANI (bandgap 2.8 eV) can absorb both ultraviolet and visible light (c) the contact electrification of Pem-iTENG in a cycle of reciprocating tidal wave, and free electrons transfer direction between the two electrodes.

2. There are less evidences to show that the efficient charge separation takes place between PANI and TiO_2. Such as surface potential difference, photovoltaic effect. Assuming that the carrier separation can take place at the interface, is it high likely that the recombination happens during operation since the seawater can work as a conductor?

Response: Thanks for your comments. Since our description of the photogenerated electrons and holes in the earlier draft is relatively rough, we provide some evidences below to explain the cause of the efficient separation and recombination of charges in our Pem-iTENG.

When TiO_2 is modified by PANI, a type-II P-N heterojunction is formed at the interface of the TiO_2/PANI. Under the illumination of sunlight, TiO_2 and PANI primarily absorb UV and visible portions of the incident light, respectively. The photogenerated electrons and holes are separated by the built-in field (Advanced Energy Materials, 2020, 10(9): 1903713. ACS Applied Energy Materials, 2018, 1(5): 2087-2095. Small, 2016, 12(42): 5809-5816. ACS Applied Energy Materials, 2018, 1(5): 2087-2095.), and they moved into TiO_2 (electrons) and PANI (holes), respectively (Fig. s3.3a,s3.3b).

When the Pem-iTENG is placed in a marine environment, the PANI covered at the surface of the bionic cilia is exposed to moist water mist or organic matter, etc (ACS Nano, 2020, 14 (3): 3630-3639). The holes (h^+) at surface of the PANI will react with water molecules to form hydroxyl radical (Fig. s3.3c ii), which contributes to the oxidation and degradation of organic molecules (see Equation 3.1-3.4), while the excess photoelectrons will exist in TiO_2 due to the TiO_2 particles being wrapped between PANI and the bionic cilia (Fig. Fig. s3.3c i). Here, we believe that a few photoelectrons retained in PANI can react with oxygen to produce superoxide radicals, but the retained photoelectrons in PANI are very few, which can be ignored.

Therefore, through the built-in electric field of the P-N heterojunction, the effective separation of photogenerated electrons and holes can be realized.

Following the above process, since the photoelectrons have no hole to pair and the PDMS film is a tribopolymer that easily absorbs negative charges without dissipating in a period of time, the photoelectrons are retained in the contact interface of bionic cilia and TiO_2, which increase the surface charge density of the PDMS film. Hence, when the tidal water wave rises, the contact seawater as a conductor and the PDMS

film construct an electric double layer to screen the negative charges on the PDMS film partly (Nano Energy, 2014, 9: 186-195), which promotes the photoelectrons induced positive charges in seawater. This coupling the physical effect of contact electrification and electrostatic induction is proposed very early and is also the theoretical support of the current triboelectric nanogenerators (Nano Energy, 2014, 9: 186-195. Nature communications, 2017, 8(1): 1-7. Nature, 2020, 578(7795): 392-396).

Therefore, since seawater as a conductor can trigger the contact electrification, the charge induction between photoelectrons (excess) and positive charges (seawater) can occur in the electric double layer while the **r**ecombination of photo-generated electrons and holes cannot happen again.

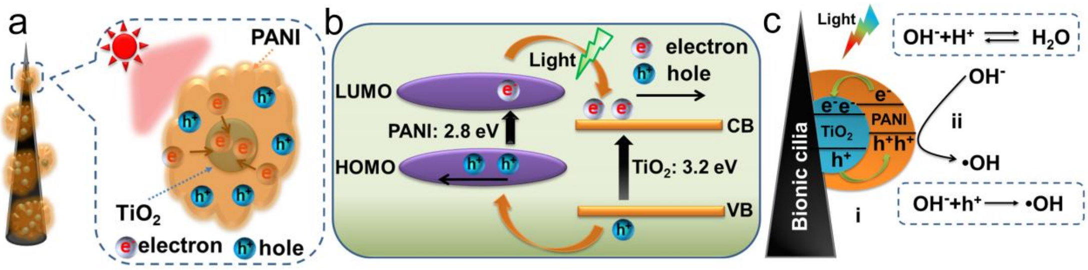

Fig. s3.3 Separation mechanism of photogenerated electrons and holes: a energy level conversion ofP-N heterojunction b schematic diagram of movement of photoelectrons and holes in the built-in field of P-N heterojunction. c charge transfer in the photocatalysis on single cilia surface.

$$\mathrm{PANI/TiO_2 + hv \rightarrow PANI^+ / TiO_2 + e^-_{CB}} \quad (3.1)$$

$$\mathrm{e^-_{CB} + O_2 \rightarrow O_2^-} \quad (3.2)$$

$$\mathrm{PANI^+/TiO_2 \rightarrow PANI/TiO_2 + h^+_{VB}} \quad (3.3)$$

$$\mathrm{h^+_{VB} + (H_2O \Leftrightarrow H^+ + OH^-) \rightarrow H^+ + OH^-} \quad (3.4)$$

3. The calculation of the “average power density” in method part is confusing. What dose the “average” mean? Since the V(t) and I(t) are the functions of the time, is it a time average or something else? Why did the authors use the peak voltage and peak current to conduct the calculation?

Response: Thanks for your comments. Because the output power of TENG has a certain relationship with the size of friction composite film. In this work, in order to describe the output power density of Pem-iTENG per unit area more properly, we calculate the output power per unit area of Pem-iTENG under different loads by using the ratio between the power density and the area of friction composites film, namely P_{avg}, unit mW/cm^2. In order to avoid ambiguity, the concept of "average power density" has been modified in the manuscript. "average" is removed in the paper, and

"power density" is used to represent the output power density per unit area of Pem-iTENG under different loads.

In order to study the output power density of Pem-iTENG, we tested the output voltage and current when connected to external load resistance. The traditional power spectral density spectrum is a probabilistic statistical method, which is a measure of the mean square value of random variables and is generally used for random vibration analysis. The continuous transient response can only be described by probability distribution function, that is, the probability corresponding to a certain level of response. The power spectral density of a signal exists only if the signal is a generalized stationary process. If the signal is not a stationary process, the autocorrelation function must be a function of two variables, so there is no power density spectral, but similar techniques can be used to estimate the time-varying density spectral. Therefore, in order to simplify the calculation process, we use the peak voltage and peak current to represent the output power density under different loads.

4. Following the aforementioned question, it is highly suspected that the energy efficiency calculation is proper used. The authors claims that the power conversion efficiency is as high as 67%. It is a very high value. Here, how do the author define the power conversion efficiency? It means the ratio of electrical power output dividing water mechanical energy as well as adding up the solar (light) illumination? I strongly suggest that the authors refer to the solar cell power conversion efficiency, it is widely accepted and applied both in industry and academy.

Response: Thanks for your comments. Compared with the photovoltaic cells, the energy conversion rate of triboelectric nano-generator is really high, but this does not mean that they have good or bad performance comparison through calculation results, because the energy conversion principles and calculation methods of the two devices are different. The energy conversion rate of solar cells can be calculated by J-V curve, but triboelectric nano-generator is mainly calculated by the unstable alternating current generated by contact electrification. In this paper, Pem-iTENG obtains mechanical energy from the solid-liquid friction process of tide wave scouring, while light only plays an auxiliary photocatalysis role in P-N heterojunction. Even if it can generate electricity after light illumination, its power generation is far less than the

magnitude of mechanical triboelectric energy, which can be basically ignored. Therefore, the calculation method of the power conversion efficiency of solar cells is not suitable for the energy conversion efficiency of Pem-iTENG proposed in this paper.

The energy conversion rate of TENG refers to the calculation method in the literature (Nature, 2020, 578(7795): 392-396), and the ratio of input energy to output energy is often used to evaluate. For example, Xu et al. developed a droplet power generation device, which uses a structure to collect energy from the impacted water droplets. In order to calculate the conversion efficiency from mechanical energy to electrical energy, the calculation method of the conversion efficiency is defined when the 1th droplet strike.

$$\eta_i = \frac{E_{out}}{E_{in}} = \frac{\int_{t_{on}}^{t_{off}} \frac{U^2}{R_L} dt}{mgh} \quad (3.5)$$

Whereby, m is the mass of the droplet which is 0.1g, g is the gravitational acceleration which is 9.8 m $s^{-2,}$ and h is the relative height of the released droplet and water which is 15.0cm. R_L is 10.0 MΩ.

Based on the above calculation method, the energy conversion rate (ECR) of Pem-iTENG can be expressed by the ratio of the output electric energy under different external load resistors to the total input energy (the sum of mechanical energy and solar energy), which can be expressed as follows:

$$\begin{aligned} ECR(\%) &= \frac{E_{out}}{E_{me}+E_{op}} \\ &= \frac{\int_{t_1}^{t_2} I^2 R dt}{\frac{1}{2}mv^2 + Pt \cdot \eta} \end{aligned} \quad (3.6)$$

Whereby, E_{out} is the output electrical energy, E_{me} is the input mechanical energy, E_{op} is the input optical energy, m is the mass of Pem-iCM, v is the speed of linear motor, P is the power of xenon lamp, and η is the utility rate of luminous energy.

It is worth noting that η in the formula refers to the utilization rate of light energy, that is, the electric energy consumed by solar energy/xenon lamp received by Pem-iCM with a value of 1.5% (J. Mater. Chem. 4, 2016, 10395). Light energy is the driving force of Pem-iCM in photocatalysis. The utilization rate of light energy generally refers to the ratio between the energy contained in organic matter produced

by crops through photosynthesis and the solar energy received by this land per unit land area. The theoretical calculation value can generally reach 6.0% ~ 8.0%, but only 0.5% ~ 1.0% in actual production, and up to 2% at most. Because Pem-iTENG is inspired by the photocatalytic principle of green plants, only part of the frequency of solar energy irradiated on Pem-iCM can be absorbed and utilized by Pem-iTENG. At the same time, solar energy irradiated on the surface is not completely absorbed, part of it is lost into the air due to reflection, and part of it is not absorbed because it passes through the friction composite film. Most of the solar energy absorbed by the friction composite film is converted into heat energy, and only a small part of the energy is utilized by photocatalysis. Therefore, the input light energy cannot be simply considered as the power consumption of xenon lamp. Here, we define a conversion parameter. That is, the utilization rate of light energy follows the concept of leaf in the photocatalysis, but it is not the same as the photoelectric conversion efficiency in the photovoltaic cells.

5. The authors claim that there are charge trsanfer/exciton dissociation at the interface between PANI and TiO_2. However, there is no experiment data either external quantum efficiency or transient optical measurement. Since it is the basic point of this manuscript, I would suggest that authors need to provide solid evidence to verify this point.

Response: At present, it is difficult to directly test charge trsanfer/exciton dissociation at the interface between PANI and TiO_2. Here, we explained this problem from the perspective of system experiments in response to this problem.

Firstly, the supplementary experiment of the charge density is performed during the fluctuation simulation test. And the charge density of Pem-iTENG with bionic cilia unmodified or modified TiO_2/PANI is tested before and after the illumination (see Fig. s3.5). The metal copper is the fixed electrodes (see Fig.s3.5a), and the output end of the electrode is connected to the test equipment through a rectifier bridge. The surface structure of single cilia indicted that the surface of bionic cilia is wrapped with a layer of modified TiO_2/PANI (see Fig.s3.5b).

The charge density of Pem-iTENG with bionic cilia unmodified TiO_2/PANI remains at 122.38 nC in dark and light (see Fig.s3.5c). This indicates that sunlight cannot enhance the charge density of Pem-iTENG after the illumination. The charge density of Pem-iTENG with bionic cilia modified TiO_2/PANI increases from 122.73

nC to 164.12 nC in the dark and light (see Fig. s3.5d), and it reveals that after modified with TiO_2/PANI, sunlight can enhance the charge density of Pem-iTENG. It is worth noting that compared with unmodified TiO_2/PANI in dark, the charge density of Pem-iTENG modified TiO_2/PANI in dark is very close. After eliminating the influence of sunlight, it indicates that factors of surface roughness, etc. have basically no effect on the charge density before and after modification with TiO_2/PANI. Therefore, we demonstrated that sunlight can excite a significant change in the charge density of our Pem-iTENG modified TiO_2/PANI during the fluctuation simulation test.

Secondly, we undergo the light effect experiments (open circuit voltage and short circuit current) during the process of light-dark-light state (Fig.s3.6). As a control group, light effect experiments of Pem-ITENG with PDMS film are carried out (Fig.s3.6a,b). The frequency of the reciprocating motion of the driving motor is set to 2 Hz. The open circuit voltage (about 15V) and short circuit current (700μA) of Pem-iTENG without bionic cilia unmodified TiO_2/PANI are not significantly changed in the light-dark-light state (see Fig.s3.6 a,b). Hence, it indicates that the increase of electron transfer is induced by photon excitation effect of the insulator surfaces can be eliminated.

After the surface covered with bionic cilia modified TiO_2/PANI, the open circuit voltage and the short circuit current of Pem-iTENG modified TiO_2/PANI are significantly improved in light compared with in dark. The open circuit voltage increases from 38.8 V in dark to 59.87 V in light, and the short circuit current increases from 2.4 mA in dark to 4.5 mA in light (see Fig.s3.6c,d). This indicates the enhancement of light effect experiments of Pem-iTENG is attributed to the photocatalysis. Therefore, compared with the improvement of the light effect experiments of Pem-iTENG with bionic cilia modified TiO_2/PANI in dark and light, we confirmed that the increase of electron transfer is induced by photocatalysis after excluding the light excitation.

Thirdly, through magnetic self-assembly technology, the solid-liquid contact angle of the PDMS film with bionic cilia is 165.28° (see Fig.s3.7c), while bionic cilia modified TiO_2/PANI is 162.04°(see Fig.s3.7d). It indicates that the PDMS films with bionic cilia modified or unmodified TiO_2/PANI have certain super-hydrophobic properties, and the modification of TiO_2/PANI has no significant effect on the solid-liquid contact angle. Moreover, PDMS film modified TiO_2/PANI with a light

irradiation result shows that the solid-liquid contact angle after the light illumination of 1h is 159.74 °, and the solid-liquid contact angle after the light illumination of 3h is 162.13 °. Comparing the contact angles of bionic cilia modified or unmodified TiO_2/PANI, it shows that the light irradiation time has no significant effect on the solid-liquid contact angle. Therefore, we can exclude the influence of the contact angle on the solid-liquid surfaces leading to the increase of electron transfer.

In summary, we have verified that a significant enhancement in the charge density of Pem-iTENG modified TiO_2/PANI is illuminated by sunlight during the fluctuation simulation test. Then, we have confirmed that the increase of electron transfer is induced by photocatalysis instead of photon excitation effect. Finally, the influence of the contact angle on the solid-liquid surfaces leading to the increase of electron transfer is excluded. Therefore, we can explain indirectly through systematic experiments that there are charge trsanfer/exciton dissociation at the interface between PANI and TiO_2. Moreover, we demonstrated that through systematic experiments, our hypothesis for the photoelectric-mechanical integrated triboelectric nano-generator is correct, and the working mechanism of the proposed Pem-iTENG is fully convincing.

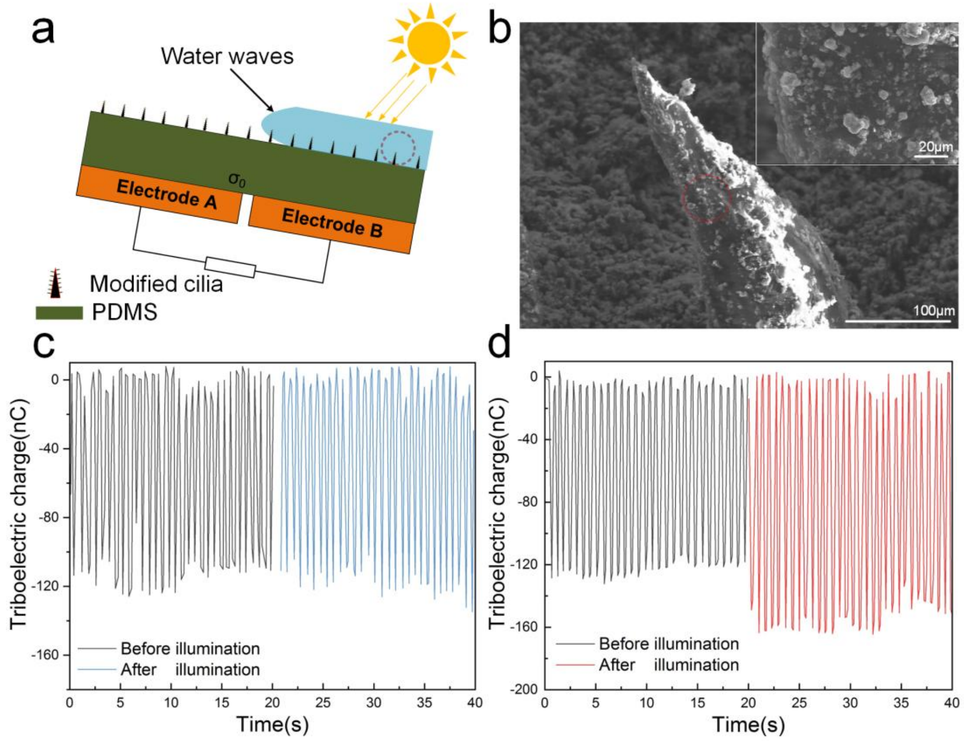

Fig.s3.5 Charge density diagram under different experimental conditions: (a) simple structure diagram of Pem-iTENG, (b) single bionic cilia modified TiO_2/PANI, (c) charge density unmodified TiO_2/PANI before and after the illumination, (d) charge density modified TiO_2/PANI before and after the illumination.

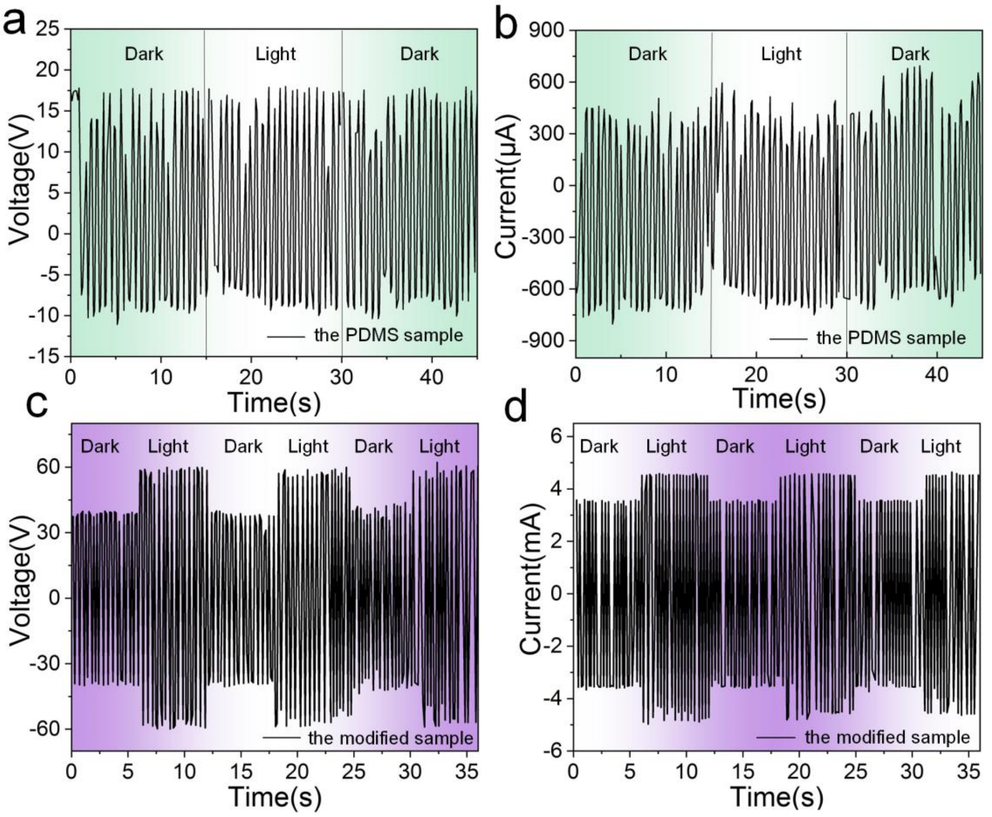

Fig.s3.6 the light effect experiments under light-darknes: (a) open circuit voltage and short circuit current (b) of PDMS without bionic cilia unmodified TiO_2/PANI, (c) open circuit voltage and short circuit current (d) of PDMS with bionic cilia modified TiO_2/PANI.

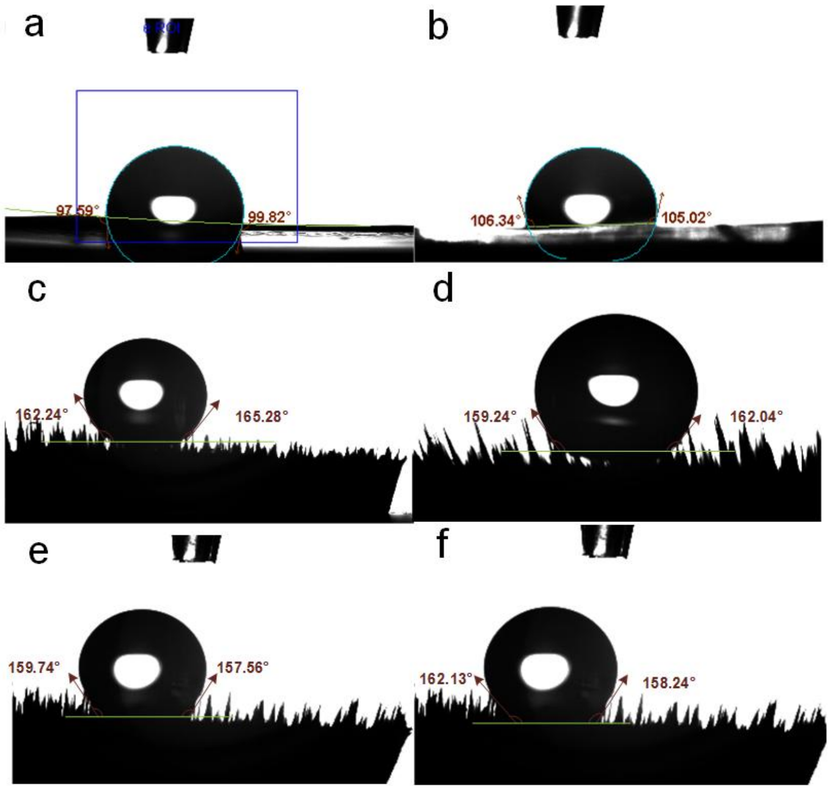

Fig. s3.7 Solid liquid contact angle of different samples: (a) the contact angle of pure PDMS, (b)

The contact angle of the PDMS doped with cobalt powder, (c) the contact angle of PDMS with bionic cilia unmodified TiO_2/PANI, (d) the contact angle of PDMS with bionic cilia modified TiO_2/PANI, (e) the contact angle of PDMS with bionic cilia modified TiO_2/PANI after illumination for 1 h, (f) the contact angle of PDMS with bionic cilia modified TiO_2/PANI after illumination for 3 h.

6. In the part of energy efficiency calculation, the data used to calculate output electrical energy are collected before rectification or after? Are there any difference between the calculated values by using the data collected before or after rectification? Please give the details. Additionally, what is the value of the "η"? And why use the "η" during input light energy calculation?

Response: Thanks to your comments. The data for calculating the output power is collected before rectification, and there are differences between the calculated results by using the data before rectification and after. According to the calculation method of the energy conversion rate of Pem-iTENG in Question 4, the energy conversion rate before and after rectification can be obtained. The calculated energy conversion rate before rectification is 67.42%, and the energy conversion rate after rectification is 62.76%. Considering the energy consumption of the rectifier and load, we used the data before rectification to calculate the energy conversion rate of the triboelectric nanogenerator. Although there is a certain deviation, the error is very small and can be ignored. The following table (Table s3.1) shows the calculation process of the energy conversion rate before and after the rectification of Pem-iTENG.

Table s3.1 Conversion rate calculation before and after rectification

Parameters / Samples	Output current (J_{sc}, μAcm^{-2})	Output energy (E_{out}, J)	Mechanical energy (E_{me}, J)	luminous energy (E_{op}, J)	Energy conversion rate(ECR)
**Pyramid-TENG**	24.5	1.038	40	0	3.27%
**PTFE-TENG**	106.1	13.564	40	0	33.91%
**3D-TENG**	160.1	21.532	40	0	53.83%
**Pem-iTENG-Before**	221.6	32.563	40.799	7.5	67.42%
**Pem-iTENG-After**	208.2	30.310	40.799	7.5	62.76%

Note: The power of the xenon lamp is 500w, the value of η is 1.5%, and the input mechanical energy of the other three TEMGs is 40 J by default.

η is the utilization rate of light energy, that is, the electric energy consumed by solar energy/xenon lamp received by Pem-iCM with a value of 1.5% (J. Mater. Chem. Cmem 4, 2016, 10395). Light energy is the driving force of photocatalysis for Pem-iCM. The utilization rate of light energy generally refers to the ratio between the

energy contained in organic matter produced by crops during photosynthesis and the solar energy received by this land per unit land area. The theoretical calculation value can generally reach 6.0% ~ 8.0%, but only 0.5% ~ 1.0% in actual production, and up to 2% at most. Because Pem-iTENG is inspired by the photocatalytic principle of green plants, only part of the frequency of solar energy irradiated on Pem-iCM can be absorbed and utilized by Pem-iTENG. At the same time, the solar energy irradiated on the surface of the friction composite film is not completely absorbed, part of it is lost into the air due to reflection, and part of it is not absorbed because it passes through the friction composite film. Most of solar energy absorbed by the friction composite film is converted into heat energy, and only a small part of the energy is utilized by the photocatalysis. Therefore, the input light energy cannot be simply considered as the power consumption of xenon lamp. Here, we define a conversion parameter. That is, the utilization rate of light energy, which is not the same concept as the photoelectric conversion efficiency in the photovoltaic cells, and has nothing to do with the conversion efficiency (η) from light energy to electric energy of solar cells (see Figure s3.8 for details).

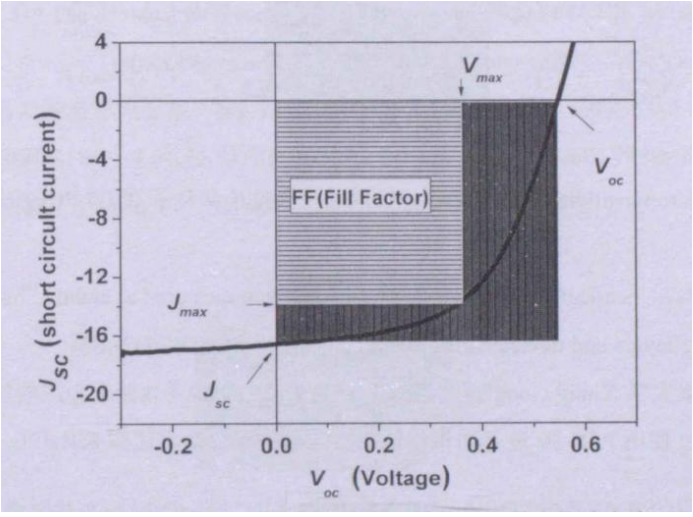

Fis. s3.8 J-V curve of ideal solar cell under illumination conditions. The performance parameters of solar cells mainly include open circuit voltage (V_{OC}), short circuit current (J_{SC}), fill factor (*FF*), power conversion efficiency (*PCE*) and external quantum efficiency (*EQE*). When the voltage is open-circuit, the measured voltage drop between both ends is the open-circuit voltage of solar cell. The conversion efficiency of the solar cell is calculated by the following formula of $\eta=P_{max}/P_{in}=V_{oc}\times I_{sc}\times FF/P_{in}$. Whereby, P_{in} is the power of the incident light. In order to obtain high conversion efficiency, the battery must have high open circuit voltage, large short circuit current, high fill factor and low dark current.

Reviewer #1 (Remarks to the Author):

The authors have addressed most of our comments. We still have the following comments:

1. In Fig. S1.2, the captain mentioned the charge density, however, the charge quantity was provided and thus charge density should be calculated. Besides, according to the account of charges measured, there is a 30% increase by the utility of illumination. Please explain why there is more than 60% increase in performance.
2. Fig 5 is a little bit confusing. Fundamentally, the input frequency of liquid will not influence the peak voltage of generator. But as shown in all the plots in Fig. 5, it appears that the peak voltage does depend on the frequency. Moreover, why the 1 Hz is associated with the largest output. Please further explain it.
The manuscript can be published if these comments can be addressed.

Reviewer #2 (Remarks to the Author):

All my questions have been addressed and the manuscript has been carefully revised. The working principle of the Pem-iTENG has been corrected. Also, the light effect experiments of Pem-iTENg with PDMS are carried out as a control group. I think the supplementary data in the revised manuscript reinforce the conclusions in this work. Since the mechanism of the Pem-iTENG is based on the electron transfer at the liquid-solid interface, I recommend two references to the authors, which support the electron transfer in the liquid-solid contact electrification. (Nature Communications, 2020, 11, 399.; Nano Energy, 2020, 76, 105070)

Reviewer #3 (Remarks to the Author):

The authors have mostly addressed my comments. However, regarding to the power conversion efficiency, I still have some different opinions from the authors. If it is not addressed properly, I believe that it is likely misleading the following reader. The authors claim that the power conversion efficiency is as high as 67%. It is a very high value. For highly applied photovoltaic device, the power conversion efficiency is only 26% (after around 70 years development, and the power generation capability yield approximately 2% of our daily electricity utilization.)
I still strongly suggest that the authors refer to the solar cell power conversion efficiency, it is widely accepted and applied both in industry and academy. TENG device power conversion efficiency can also defined by IV curve.

Response to Reviewers' Comments (NCOMMS-20-13459B):

Reviewer #1

The authors have addressed most of our comments. We still have the following comments:

1. In Fig. S1.2, the captain mentioned the charge density, however, the charge quantity was provided and thus charge density should be calculated. Besides, according to the account of charges measured, there is a 30% increase by the utility of illumination. Please explain why there is more than 60% increase in performance.

2. Fig 5 is a little bit confusing. Fundamentally, the input frequency of liquid will not influence the peak voltage of generator. But as shown in all the plots in Fig. 5, it appears that the peak voltage does depend on the frequency. Moreover, why the 1 Hz is associated with the largest output.

Please further explain it. The manuscript can be published if these comments can be addressed.

1. In Fig. S1.2, the captain mentioned the charge density, however, the charge quantity was provided and thus charge density should be calculated. Besides, according to the account of charges measured, there is a 30% increase by the utility of illumination. Please explain why there is more than 60% increase in performance.

Response: Thanks so much for your good suggestions. **Firstly,** the charge density of Pem-iTENG has been calculated with the provided charge quantity according to the reviewers' requirement (see Table S2).

Secondly, the reasons of the different performance indicators of Pem-iTENG are explained as follows.

The **30%** increase in electrical properties of Pem-iTENG mainly refers to the improved **performance indicator** of our Pem-iTENG **in our work** (see Table S2). **Specifically,** the charge density of Pem-iTENG modified TiO_2/PANI in dark and in light increased from 14.267 nC cm^{-2} to 18.296 nC cm^{-2}, which increased by 28.23%. The charge density of Pem-iTENG unmodified TiO_2/PANI in dark and modified TiO_2/PANI in light increased from 13.395 nC cm^{-2} to 18.296 nC cm^{-2}, which

increased by 36.58%.

However, the **60%** increase in electrical performance mainly refers to **comparison** results of **previous reports** and **our work** on power conversion efficiency by using the same calculation method (*Nature, 2020, 578(7795): 392-396*). **Specifically,** the maximum energy conversion efficiency of Pem-iTENG, PTFE-TENG, 3D-TENG, and Pyramid TENG is 67.4%, 53.8%, 33.9%, and 11.4%, respectively. The power conversion efficiency of Pem-iTENG is about 6 times higher than these of typical TENGs.

Therefore, the numerical values of 30% and 60% represent different performance indicators in different objects, which are not related.

Table S2 The charge density of different types of Pem-iTENGs before and after light illumination

Materials	Illumination state [Dark/light]	Current [mA]	Q_{sc} [nC]	Charge Density [nC cm^{-2}]
Unmodified TiO_2/PANI	D	3.42	120.56	13.395
Unmodified TiO_2/PANI	L	3.49	121.11	13.457
Modified TiO_2/PANI	D	3.53	128.41	14.267
Modified TiO_2/PANI	L	4.52	164.67	18.296

2. Fig 5 is a little bit confusing. Fundamentally, the input frequency of liquid will not influence the peak voltage of generator. But as shown in all the plots in Fig. 5, it appears that the peak voltage does depend on the frequency. Moreover, why the 1 Hz is associated with the largest output. Please further explain it.

Response: Thanks so much for your good suggestions. The main reasons can be explained as follows.

The working process of solid-liquid contact-based TENGs by the water scouring is that a continuous contact and separation cycle occurs between the water wave and solid-polymers, resulting in the generation of the contact electrification in solid-liquid contact-based TENGs (Angewandte Chemie, 2013, 52(48): 12545-12549. Nano Energy, 2020, 77: 105093.). Hence, the dynamic interaction factors between the liquid-water-wave and the solid-polymers, such as the hydrophobicity and surface structure of solid-polymers, the polarity of liquids, water wave motion, etc. can affect the electrical properties of solid-liquid contact-based TENGs (ACS nano, 2014, 8(6): 6031-6037. ACS nano, 2015, 9(7): 7671-7677. Nano Energy, 2020: 104748.

Advanced Functional Materials, 2018, 28(35): 1803117. ACS nano, 2018, 12(3): 2893-2899.).

In order to investigate the effect of the reciprocating tidal waves on Pem-iTENG, a water wave simulation device was designed in the experiment. Since the contact frequency of tidal waves in the ocean is not fixed, the frequency of water waves is changed to explore the influence of unstable tidal wave on the electrical performance of Pem-iTENG. Here, it is generally believed that the open circuit voltage largely depends on the charge density in the contact electrification (Nano energy, 2018, 44:388-398). In an ideal situation, we default that the dynamic interaction factors are unchanged, and the charge density remains stable in each cycle. **Therefore**, in this case, we believe that the contact frequency of water wave will not affect the peak voltage of TENGs, and the peak voltage is relatively stable.

In Fig.5a-c, the dynamic interaction factors remain unchanged, and the contact frequency of the water wave is changed to simulate the impact of tidal wave motion on the power generation performance of Pem-iTENG. A large number of experimental evidences revealed that the charge density generated by solid-liquid contact area is the main factor affecting the contact electrification of Pem-iTENG at low frequency water wave (ACS nano，2015，9(7)：7671-7677)**. In this case**, as the contact frequency of water waves increases **(<1 Hz)**, the contact area in contact electrification increases. When the water wave is at 1 Hz, the water wave just covers the entire contact area. The open circuit voltage reached the maximum value at 1 Hz. However, when the contact frequency of water wave continues to increase **(>1 Hz),** the charge density with opposite polarities on Pem-iCM and solution surface is easily neutralized due to the parts of solution retained on Pem-iCM at high water wave frequency, resulting in fluctuation of the peak voltage (Nano Energy, 2018, 44: 388-398).

Therefore, Pem-iTENG at 1 Hz is associated with the largest the peak voltage output in the experiment.

Reviewer #2:

All my questions have been addressed and the manuscript has been carefully revised. The working principle of the Pem-iTENG has been corrected. Also, the light effect experiments of Pem-iTENg with PDMS are carried out as a control group. I think the supplementary data in the revised manuscript reinforce the conclusions in this work. Since the mechanism of the Pem-iTENG is based on the electron transfer at the liquid-solid interface, I recommend two references to the authors, which support the electron transfer in the liquid-solid contact electrification. (Nature Communications, 2020, 11, 399.; Nano Energy, 2020, 76, 105070).

Response: Thanks so much for your good suggestions. We have supplemented the references (**see Ref. 32, 33**), which support the electron transfer in the liquid-solid contact electrification in the paper.

Reviewer #3:

The authors have mostly addressed my comments. However, regarding to the power conversion efficiency (PCE), I still have some different opinions from the authors. If it is not addressed properly, I believe that it is likely misleading the following reader. The authors claim that the power conversion efficiency is as high as 67%. It is a very high value. For highly applied photovoltaic device, the power conversion efficiency is only 26% (after around 70 years development, and the power generation capability yield approximately 2% of our daily electricity utilization.)

I still strongly suggest that the authors refer to the solar cell power conversion efficiency, it is widely accepted and applied both in industry and academy. TENG device power conversion efficiency can also define by IV curve.

Response: Thanks so much for your good proposal. According to the reviewer's suggestion, we adopted the evaluation method of power conversion efficiency in solar cells and recalculated the power conversion efficiency of Pem-iTENG. By referring to the power conversion efficiency of solar cells (*Angewandte Chemie, 2011, 123(24): 5633-5637, Nature communications, 2013, 4(1): 1-10.*), the calculation method of the power conversion efficiency of Pem-iTENG is provided, as shown in equation (2) and (3).

$$FF = \frac{V_{max} \times J_{max}}{V_{oc} \times J_{sc}} \times 100\% \tag{2}$$

Whereby, *FF* stands for fill factor, which is a physical quantity that measures the external load capacity of TENGs. The capacity of the external load of the device can be compared according to the value of *FF*. The relationship between the power conversion efficiency (*PCE*) and the fill factor (*FF*) is as follows.

$$PCE(\%) = \frac{P_{\max} \times 100\%}{P_{in}} = \frac{V_{oc} \times J_{sc} \times FF}{P_{in}} \times 100\% \tag{3}$$

Whereby, P_{max} is the maximum output power of the device under an external load. Open circuit voltage (V_{oc}) refers to the potential between the two electrodes of the device with the open external circuit. Short circuit current (J_{sc}) refers to the current value through the device when the external circuit is short-circuited. P_{in} is the input power of the device.

Here, the current density-voltage (*J-V*) curve of Pem-iTENG under various external

load of $10^2\ \Omega$ to $10^9\ \Omega$ was developed in a solar light intensity and tide wave (Fig.6b). Therefore, according to the evaluation method in solar cells, our Pem-TENG produces the power conversion efficiency of 16.72%, the open circuit voltage (V_{oc}) of 124.2 V, and the short circuit current density (J_{sc}) of 221.6 μA cm^{-2}, and the fill factor (FF) of 62.6%.

By extracting the *PCE* parameters in the references (details see Table S3), we calculated the *PCE* values of Pem-iTENG and typical TENGs (see Fig. S8). Results show that the power conversion efficiency of Pem-iTENG, PTFE-TENG, 3D-TENG, and Pyramid TENG is 16.72%, 12.59%, 7.33%, and 10.69%, respectively. The energy conversion efficiency of Pem-iTENG is about 2.28 times higher than these of typical TENGs, which highly demonstrates that our Pem-iTENG exhibits efficient energy conversion efficiency.

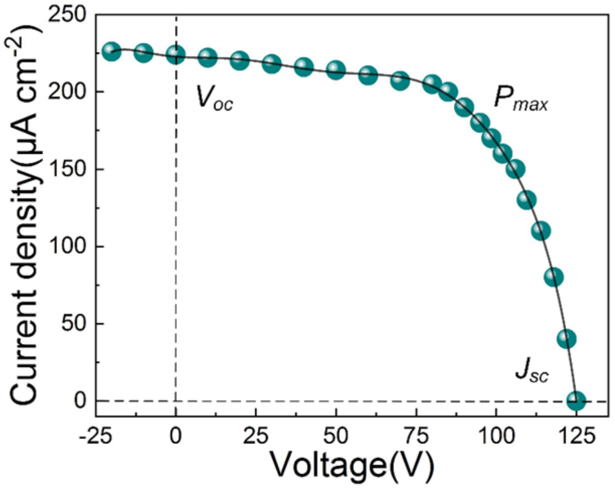

Fig. 6b current density-voltage (*J-V*) curve of Pem-iTENG in a solar light intensity and tide wave

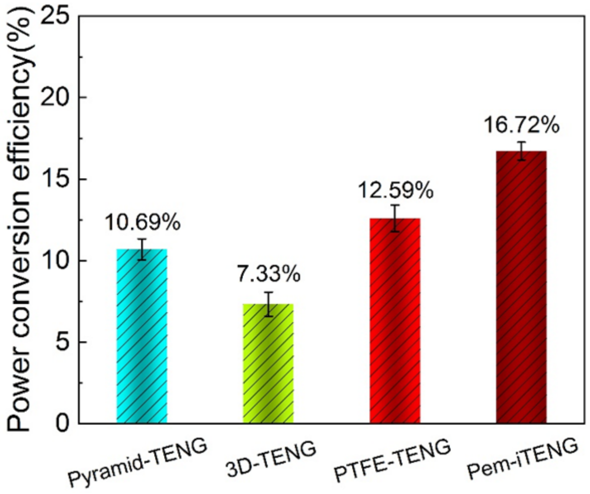

Fig.S8 Comparative analysis of PCE between Pem-iTENG and traditional TENGs with outstanding electrical performance

Table S3 Power conversion efficiency parameters of Pem-iTENG and typical TENGs.

TENGs	V_{oc} [V]	J_{sc} [μA cm^{-2}]	P_{max} [mW cm^{-2}]	FF [%]	P_{in} [mW cm^{-2}]	PCE [%]
**Pyramid TENG**	52	0.245	0.005	39.24	0.0467	10.69
**3D-TENG**	221	16.1	2.2	61.18	30	7.33
**PTFE-TENG**	13.96	106.18	0.962	64.90	7.64	12.59
**Pem-iTENG**	124.2	221.6	17.23	62.60	103.012	16.72

***Note:**

(1) Since the specific input parameters of the total input power (P_{in}) are missed in the report, the output power value of 60 LED (electrical parameters used in the paper) is adopted as the total input power value of Pyramid TENG. In this way, the calculated result of Pyramid TENG (10.69%) is slightly higher than the real PCE value.

(2) The total input power (P_{in}) of Pem-iTENG can be calculated using the equation of $P_{in} = P_m / S + P_{light}$. Whereby, P_m is the output power of the linear motor that simulates water waves (3.5 W), S is the area of the water wave baffle (50 cm^2). P_{light} is the illumination intensity of solid-polymer surface when the xenon lamp simulates solar illumination (33.012 mW cm^{-2}). Hence, the total input power of P_{in} can be calculated as 103.012 mW cm^{-2}.

Response to Reviewers' Comments (NCOMMS-20-13459A):

Reviewer #1:

The general scope of the paper is well justified and of interest to a sufficiently broad audience. The main finding of this study appears to comprehensively collect solar energy and tidal energy by introducing a cilia-like structure as well as a p-n heterojunction. There are several issues that I believe need to be addressed before the publishing of this manuscript.

1. Motivation. The advantages of this work lie in the combination of the photoelectric and triboelectric effects. However, the photoelectric effect relies on the sunlight, thus why not harness the solar energy directly since it has much higher energy density. Thus, the motivation of this work should be elaborated.

Response: Thank you for your comments. Firstly, although solar energy has a higher energy density, it has to be said that the use of solar energy is uncertain, especially under some cloudy and rainy conditions, and solar energy cannot achieve continuous energy collection. Relatively speaking, tidal energy is relatively stable and can be used stably under cloudy and rainy conditions.

Secondly, this work is to propose a photoelectric and electromechanical integrated energy system to improve energy conversion efficiency, and it is oriented to the comprehensive collection of solar energy and tide energy. Although solar energy has a relatively high energy density (especially the development in photovoltaic cells), there is a certain limit to the improvement of its photoelectric conversion rate. Compared with single solar power generation, we believe that the comprehensive utilization of nature energies is a massive potential from the perspective of energy utilization efficiency.

Finally, from the perspective of improving the energy conversion efficiency of the triboelectric nanogenerators, our Pem-iTENG is a new creation. This method is achieved by the superposition of the photocatalysis and the contact electrification to increase the surface charge density. Specifically, the charge density in the PDMS film with bionic cilia modified TiO_2/ PANI is greatly enhanced by the accumulation of photoelectrons in the photocatalysis and the triboelectric negative charges in the contact electrification. This provides a new idea for the further design of solid-liquid

based TENGs in contact electrification.

2. As shown in Fig.2, the surface of the device is superhydrophobic due to the design of cilia, which is used to increase the contact area and more friction as claimed by the authors. In fact, according to the Cassie and Wenzel theory, the superhydrophobic property relies on more air pocket, thus the real contact area between water and solid might be reduced rather than increased. Thus, is the superhydrophobic property is really the optimized design?

Response: Thanks for your comments. From the wetting theory, we know that, the water droplets contact the surface in a wetting mode as if nailed to the surface in the Wenzel state (see Fig s1.1a), while the water droplets contact the surface in a non-wetting style in the Cassie state, and the water droplets are easily rolled away from the surface due to the small adhesion force at this time (see Fig s1.1b).

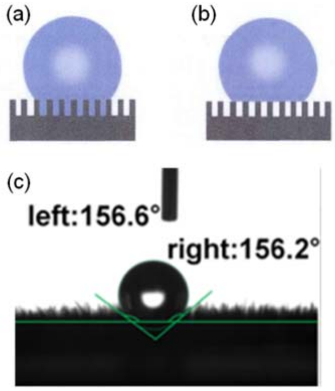

Fig s1.1 wetting theory models: (a) Wenzel state, (b) Cassie state, (c) solid-liquid contact angle of experimental sample.

Since our design is aimed at the rolling friction of the reciprocating motion of tidal water, the main advantages focus on these two aspects. Firstly, the horizontal or inclined surface motion of tidal water is different from the frontal impact of liquid droplets. This horizontal reciprocating motion does increase the air pocket contact in the surface-plane contact compared to the flat-plane contact. Secondly, considering the adhesion force and contact angle hysteresis of the droplets, seawater on the bionic cilia surface can have minimize residue droplets after reciprocating scouring.

Therefore, in order to express our design of superhydrophobic properties in this article more clearly, we have modified the description in the article.

3. Mechanisms. As illustrated in Fig.1d, initially at the state 1 the PDMS surface possess plenty of negative charges, the metal electrodes should carry induced charges and there is no output. The output only occurs when the water comes in which dynamically changes the electrical double layer. If the output is indeed from the contact electrification according to what the authors pointed out, the output is actually very low. However, the authors showed that the output without illumination is still about 7 times higher than other devices. Thus, I would tend to think the mechanism might be quite similar to the recent report on the electricity generation based on a droplet (Wanghuai Xu, et al, Nature), in which the droplet gates the charge transfer. I strongly suggest the authors to measure the charge density before and after illumination, as well as the charges generation during the wave simulate test.

Response: Thanks for your comments. The principle of the solid-liquid contact nano-generator is roughly explained in the following two ways. A working model is based on the fact that liquid droplets constantly strike on the surface of the friction polymer film, and the contact electrification phenomenon occurs (Nano Energy, (2017) 36, 250-259). Another solid-liquid contact model is that the water flow continuously washes the friction polymer film by the principle of electrostatic induction to collect electric charges, and an alternating current is generated (Nano Energy (2014) 9, 186-195). In fact, the working principle of the power generation driven by liquid droplets or water waves is similar, and the output electrical performance is realized by the contact electrification.

From the theoretical point of view, Xu et al in Nature's work is more inclined to the former. Because of the contact electrification between the droplets and the friction polymer film, the alternating current is induced between the electrodes. But our work is the latter, the principle of electrostatic induction collects triboelectric charges by water flow and produces an alternating current between two electrodes. However, it must be said that Xu et al in Nature's work and ours work have their own innovations.

Xu et al proposed a new type of power generation by droplet impact like a triode. This nano-generator consists of a three-layer structure of aluminum (Al) electrode, polytetrafluoroethylene (PTFE) film and indium tin oxide (ITO) electrode, and this structure is very similar to the field effect transistor structure (FET triode). It is composed of three electrodes, a gate, a source, and a drain. The basic principle is to

control the formation of the channel between the source and the drain by adjusting the gate voltage on the gate, and further control the flow of charges between the two electrodes.

Our work mainly proposes a photoelectric and electromechanical integrated energy system. That is to say, the working principle is attributed to the superposition of the photocatalysis and the contact electrification to increase the surface charge density (see Fig.1). Specifically, the charge density in the PDMS film with bionic cilia modified TiO_2/ PANI is greatly enhanced by the accumulation of photoelectrons in the photocatalysis and the triboelectric negative charges in the contact electrification. Subsequently, the asymmetric distribution of induced charges between the two electrodes establishes an unbalanced potential, which drives the transfer of electrons, leading to the generation of the alternating current. The experimental results show that under the tidal waves and sunlight, our Pem-iTENG shows a maximum open-circuit voltage of 124.2 V, and a maximum short-circuit current density of 221.6 μA cm^{-2}, which improves nearly 10 times. More importantly, our Pem-iTENG has higher energy conversion efficiency of 67.4%, which increases nearly 6 times. Therefore, our Pem-iTENG has certain advantages in energy conversion efficiency and comprehensive utilization of different natural energy sources.

In addition, we also carefully read and quoted recent literature on droplet power generation (Wanghuai Xu, et al, Nature) obtaining a lot of inspiration. The author realized a large industry through a small drop of liquid. We believe that this type of droplet power generation will lead the revolution in the solid-liquid contact power generation. We hope that our proposed photoelectric-electromechanical integrated triboelectric nano-generator can also provide a certain reference in this field, and can trigger more inspiration for solid-liquid contact power generation.

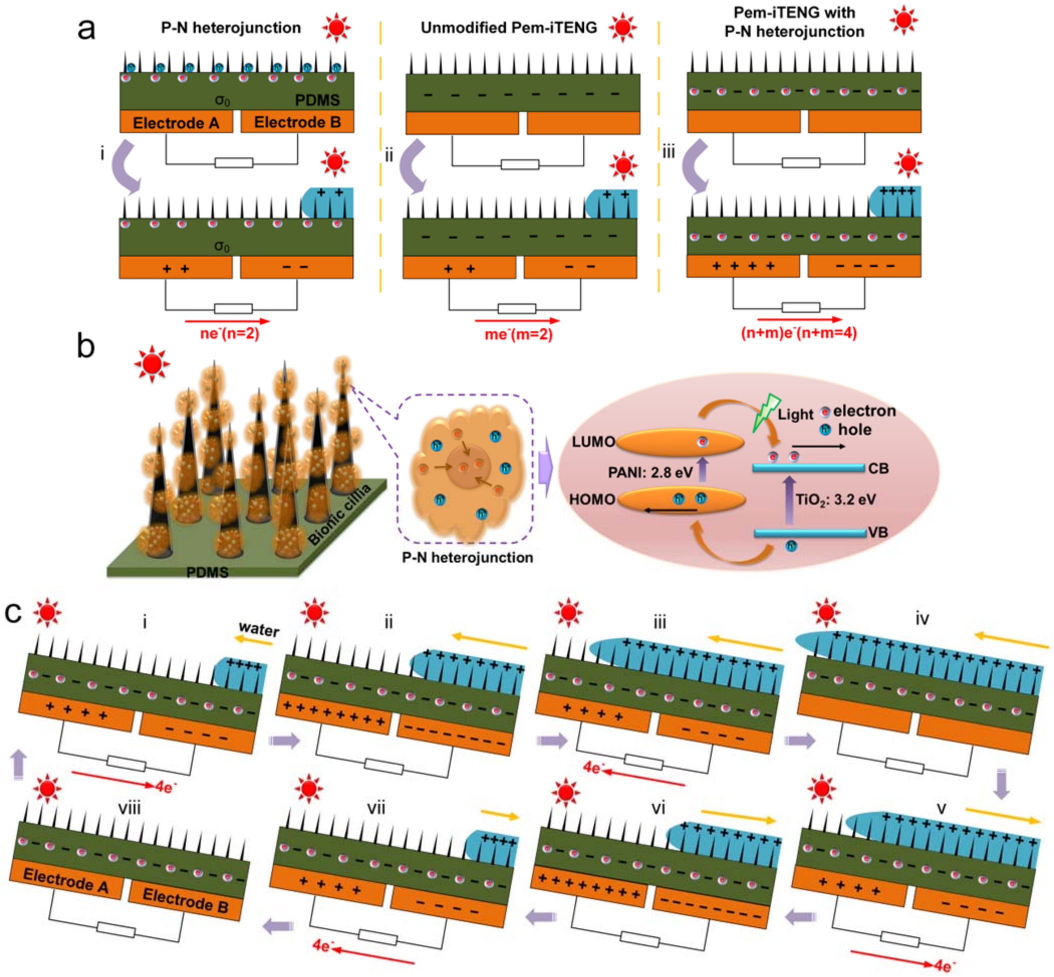

Fig. 1 the working principles of Pem-iTENG. (a) Pem-iTENG with P-N heterojunction (i), unmodified and modified Pem-iTENG during the two flow states of the tidal waves (ii and iii). (b) principles of photocatalysis of bionic cilia with P-N heterojunction. TiO_2 (bandgap 3.2 eV) can only absorb ultraviolet light, while PANI (bandgap 2.8 eV) can absorb both ultraviolet and visible light (c) the contact electrification of Pem-iTENG in a cycle of reciprocating tidal wave, and free electrons transfer direction between the two electrodes.

Finally, we supplemented the experiment as requested by the reviewers.

Firstly, the supplementary experiment of the charge density is performed during the fluctuation simulation test. And the charge density of Pem-iTENG with bionic cilia unmodified or modified TiO_2/PANI is tested before and after the illumination (see Fig. s1.2). The metal copper is the fixed electrodes (see Fig.s1.2a), and the output end of the electrode is connected to the test equipment through a rectifier bridge. The surface structure of single cilia indicted that the surface of bionic cilia is wrapped with a layer of modified TiO_2/PANI (see Fig.s1.2b).

The charge density of Pem-iTENG with bionic cilia unmodified TiO_2/PANI

remains at 122.38 nC in the dark and light (see Fig.s1.2c). This indicates that sunlight cannot enhance the charge density of Pem-iTENG after the illumination. The charge density of Pem-iTENG with bionic cilia modified TiO_2/PANI increases from 122.73 nC to 164.12 nC in the dark and light (see Fig. s1.2d), and it reveals that after modified with TiO_2/PANI, sunlight can enhance the charge density of Pem-iTENG. It is worth noting that compared with unmodified TiO_2/PANI in dark, the charge density of Pem-iTENG modified TiO_2/PANI in the dark is very close. After eliminating the influence of sunlight, it indicates that factors of surface roughness, etc. have basically no effect on the charge density before and after modification. Therefore, we demonstrate that sunlight can excite a significant change in the charge density of our Pem-iTENG modified TiO_2/PANI during the fluctuation simulation test.

Secondly, we undergo the light effect experiments (open circuit voltage and short circuit current) during the process of light-dark-light state (Fig.s1.3). As a control group, light effect experiments of Pem-ITENG with PDMS film are carried out (Fig.s1.3a,b). The frequency of the reciprocating motion of the driving motor is set to 2 Hz. The open circuit voltage (about 15V) and short circuit current (700μA) of Pem-iTENG without bionic cilia unmodified TiO_2/PANI are not significantly changed in the light-dark-light state (see Fig.s1.3 a,b). Hence, it indicates that the increase of electron transfer is induced by photon excitation effect of the insulator surfaces can be eliminated.

After the surface covered with bionic cilia modified TiO_2/PANI, the open circuit voltage and short circuit current of Pem-iTENG modified TiO_2/PANI are significantly improved in light compared with in dark. The open circuit voltage increases from 38.8 V in dark to 59.87 V in light, and the short circuit current increases from 2.4 mA in dark to 4.5 mA in light (see Fig.s1.3c,d). This indicates the enhancement of light effect experiments of Pem-iTENG is attributed to the photocatalysis. Therefore, compared with the improvement of the light effect experiments of Pem-iTENG with bionic cilia modified TiO_2/PANI in dark and light, we confirmed that the increase of electron transfer is induced by photocatalysis after excluding the light excitation.

Thirdly, through magnetic self-assembly technology, the solid-liquid contact angle of the PDMS film with bionic cilia is 165.28° (see Fig.s1.4 c), while bionic cilia modified TiO_2/PANI is 162.04° (see Fig.s1.4d). It indicates that the PDMS films with bionic cilia modified or unmodified TiO_2/PANI have certain super-hydrophobic properties, and the modification of TiO_2/PANI has no significant effect on the

solid-liquid contact angle. Moreover, PDMS film modified TiO_2/PANI with a light irradiation result shows that the solid-liquid contact angle after the light illumination of 1h is 159.74°, and the solid-liquid contact angle after the light illumination of 3h is 162.13°. Comparing the contact angles of bionic cilia modified or unmodified TiO_2/PANI, it shows that the light irradiation time has no significant effect on the solid-liquid contact angle. Therefore, we can exclude the influence of the contact angle on the solid-liquid surfaces leading to the increase of electron transfer.

In summary, we have verified that a significant enhancement in the charge density of Pem-iTENG modified TiO_2/PANI is illuminated by sunlight during the fluctuation simulation test. Then, we have confirmed that the increase of electron transfer is induced by photocatalysis instead of photon excitation effect. Finally, the influence of the contact angle on the solid-liquid surfaces leading to the increase of electron transfer is excluded. Therefore, we demonstrate that through systematic experiments, our hypothesis for the photoelectric-mechanical integrated triboelectric nano-generator is correct, and the working mechanism of the proposed Pem-iTENG is fully convincing.

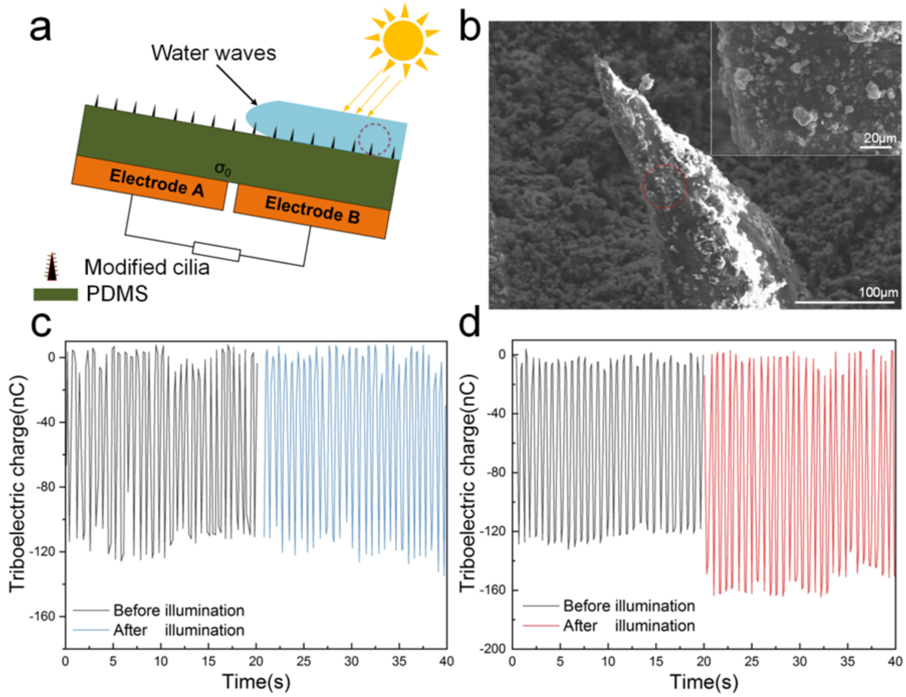

Fig.s1.2 Charge density diagram under different experimental conditions: (a) simple structure diagram of Pem-iTENG, (b) single bionic cilia modified TiO_2/PANI, (c) charge density unmodified TiO_2/PANI before and after the illumination, (d) charge density modified TiO_2/PANI before and after the illumination.

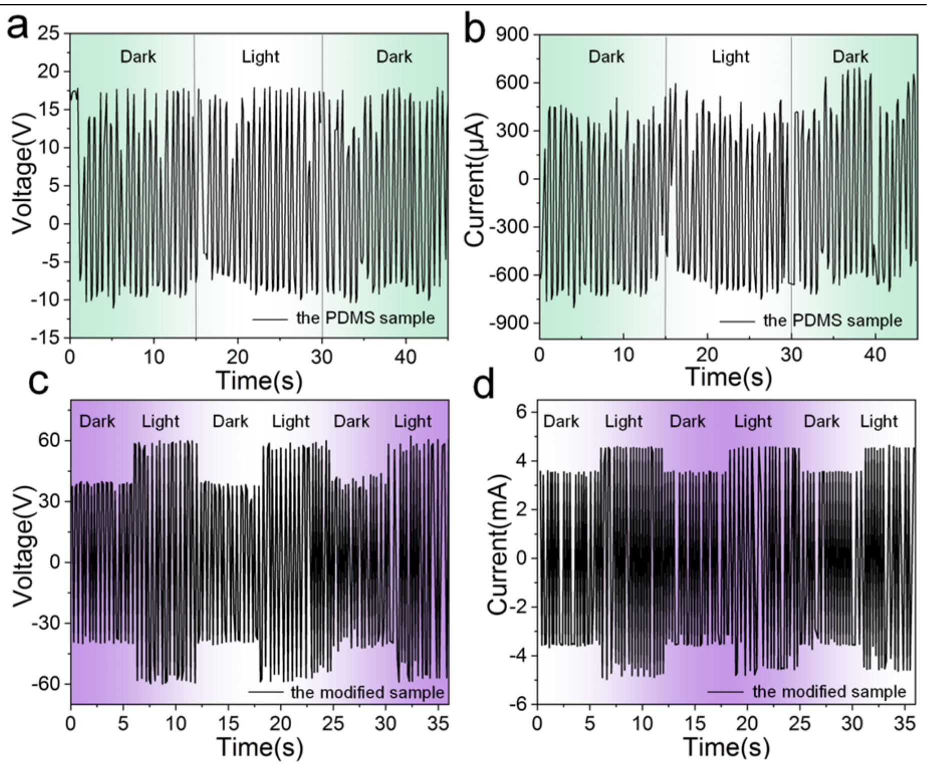

Fig.s1.3 the light effect experiments under light-darkness: (a) open circuit voltage and short circuit current (b) of PDMS without bionic cilia unmodified TiO_2/PANI, (c) open circuit voltage and short circuit current (d) of PDMS with bionic cilia modified TiO_2/PANI.

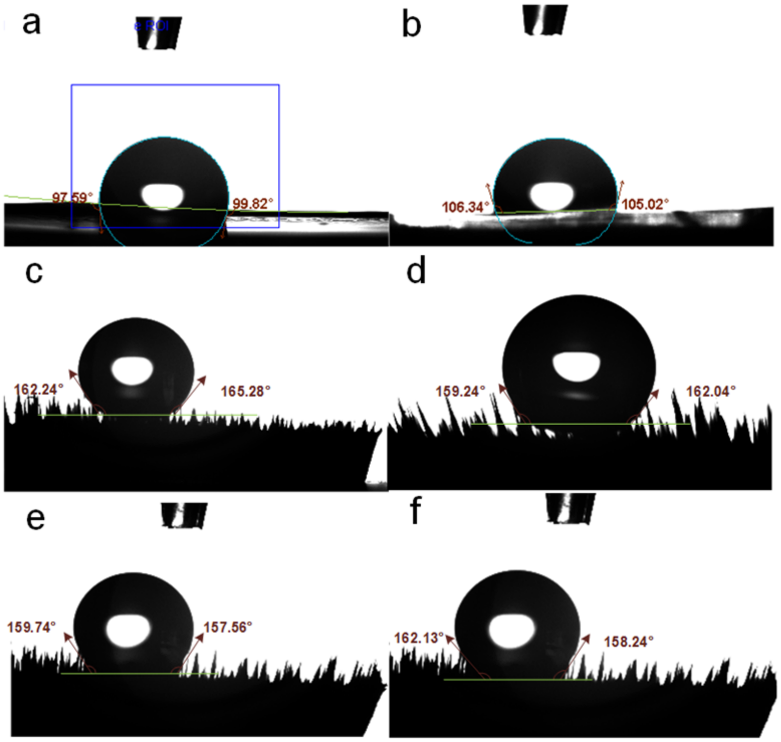

Fig. s1.4 Solid liquid contact angle of different samples: (a) the contact angle of pure PDMS, (b)

the contact angle of the PDMS doped with cobalt powder, (c) the contact angle of PDMS with bionic cilia unmodified TiO_2/PANI, (d) the contact angle of PDMS with bionic cilia modified TiO_2/PANI, (e) the contact angle of PDMS with bionic cilia modified TiO_2/PANI after illumination for 1 h, (f) the contact angle of PDMS with bionic cilia modified TiO_2/PANI after illumination for 3 h.

4. The stability of the device should be considered, as TiO_2 is always regarded to accelerate degradation of polymer (PDMS in your device).

Response: Thank you for your suggestion. TiO_2 does accelerate the degradation of PDMS. The photo-generated electrons in the valence band are excited to the conduction band, leaving a positively charged hole. The photo-generated holes then lead to the formation of free radicals. These free radicals have strong oxidizing properties and can break the chemical bonds of organic molecules. At the same time, the photo-generated electron-hole pairs generate water molecules and hydroxyl groups on the surface under ultraviolet radiation, and these activated molecules partially leave the siloxane bonds of the surrounding PDMS molecules. However, due to the excellent stabilities of the PDMS film in thermal, chemical and photocatalytic degradation, the degradation rate of PDMS by TiO_2 is not too fast, as reported in the literature (Advanced Materials 29.16(2017):1604637).

At the same time, when the critical mass of PDMS is about 30-40 wt%, it can form a thick enough polymer layer, which maintains a rough surface morphology, and obtains the largest water contact angle, inhibit photodegradation. This result is consistent with the critical mass of 30 wt% of PDMS's reported by Qing et al. (Colloids and Surfaces A, 2015, 484: 471-477). The interaction between TiO_2 and PDMS is the hydrogen bonding between the hydroxyl groups adsorbed on the surface of TiO_2 and the oxygen atoms of the PDMS backbone. Therefore, through a wide range of intermolecular interactions, high surface coverage, excellent main chain flexibility, and optimal ratio, PDMS can inhibit the photo-degradation caused by TiO_2 particles. Additionally, many methods can inhibit or reduce photo-degradation. For example, the formulations of adding CeO_2 nanoparticles forming super-hydrophobic (Molecular Systems Design & Engineering, 2020, 5(4): 876-881).

Therefore, the problem of TiO_2 accelerated the degradation of the polymer (PDMS in the device) can be solved.

5. Why the changes of OV and SA are out of sync, wherein the biggest OV appears when frequency of fluctuation is 1 Hz and SA at 2 Hz.

Response: Based on previous studies, the open-circuit voltage and short-circuit current of solid-liquid contact triboelectric nano-generator are not necessarily linear. For example, Ahmed, et al. designed a fully enclosed duck-shaped TENG that can effectively collect wave energy from random and low-frequency water (Adv. Energy Mater. 6 (2016) 1601705). The open-circuit voltage in 1.95 Hz is lower than that in 2.50 Hz, but the short-circuit current does not change significantly under the two frequencies. From the perspective of traditional electricity, the output voltage is the largest at a certain frequency, and then, the output current must also be the largest at this moment. However, Pem-iTENG has a novel working mechanism in the complex and changeable test environment, which is somewhat different in the traditional cognition.

There are several reasons that may explain this phenomenon. From the perspective of the test method, the electrical performance of our Pem-iTENG is not measured at the same time but separately tested. From the perspective of working conditions, the frequency of flooding the photoelectric-electromechanical integrated composite membrane (Pem-iCM) is different in water fluctuation frequencies, and the number of photo-generated electron-hole pairs separated under illumination is also different, so the efficiency of light energy and mechanical energy conversion to electrical energy is not necessarily the same at different frequencies. Besides, it is difficult for the Pem-iTENG to fully exert its excellent photoelectric conversion characteristics when completely submerged in water, and it also has some challenges to avoid various changes introduced in the modification.

Therefore, the open-circuit voltage reaches the peak at the frequency of the water wave at 1 Hz, and the short-circuit current reaches the maximum at the water fluctuation frequency at 2 Hz.

Reviewer #2:

A bio-inspired liquid-solid photoelectric electromechanical integrated triboelectric nano-generator (Pem-iTENG) is proposed. It is claimed that the electron transfer between water and the modified samples is enhanced by the photocatalysis. A new idea for improving the output performance of the TENGs is presented in this work. However, the experiments in this manuscript is not systematically carried out, and the working mechanism of the Pem-iTENG is not fully convincing (R2-F1). The biggest problem in this work is lack of the controlled experiments to verify the hypothesis. The only evidence is the enhanced output after illuminated by light. As we know that the contact electrification between liquid and solid is very sensitive to the contact angle of the solid surfaces. The modification of TiO_2/PANI, and light irradiation may change the contact angle of the solid surfaces and lead to the increase of electron transfer. Therefore, it is suggested that the contact angle of the PDMS without cilia, TIO_2/PANI modified PDMS with and without light irradiation should be measured to exclude the effect of contact angle on the electron transfe (R2-F2). Also, it is demonstrated that the photon excitation effect of the insulator surfaces may lead to change of the electron transfer between two contact materials. In order to confirm that the increase of electron transfer is induced by photocatalysis instead of photon excitation effect, the light effect experiments (Fig. 3a, 3b) of the unmodified sample surfaces should be tested as a control group (R2-F3). I think this manuscript cannot be published in current version. Following are my additional questions and concerns:

Response R2-F1: Thank you for your comments. We have demonstrated that a significant enhancement in the charge density of Pem-iTENG modified TiO_2/PANI is illuminated by sunlight during the fluctuation simulation test (details see Fig. s2.9 and R2-Q4). Then, we have confirmed that the increase of electrons transfer is induced by photocatalysis instead of photon excitation effect (details see Fig. s2.2 and R2-F2). Finally, we have excluded the influence of the contact angle on the solid-liquid surfaces leading to the increase of electrons transfer (details see Fig. s2.1 and R2-F3). Therefore, through systematic controlled experiments, we demonstrate that our hypothesis for the photoelectric-mechanical integrated triboelectric nano-generator is correct, and the working mechanism of the Pem-iTENG is fully convincing.

Response R2-F2: In order to exclude the influence of light irradiation and surface modification of TiO_2/PANI on the solid-liquid contact angle, we supplement the test of the solid-liquid contact angle in the following six samples, as shown in Fig. s2.1.

Through magnetic self-assembly technology, the solid-liquid contact angle of the PDMS film with bionic cilia is 165.28°(see Fig.s2.1(c)), while bionic cilia e modified TiO_2/PANI is 162.04°(see Fig.s2.1(d)). It indicted that the PDMS films with bionic cilia modified or unmodified TiO_2/PANI have certain super-hydrophobic properties, and the modification of TiO_2/PANI has no significant effect on the solid-liquid contact angle.

Moreover, PDMS film modified TiO_2/PANI with a light irradiation results show that the solid-liquid contact angle after the light illumination of 1 h is 159.74°, and the solid-liquid contact angle after the light illumination of 3 h is 162.13°. Comparing the contact angles of bionic cilia modified or unmodified TiO_2/PANI, it shows that the light irradiation time has no significant effect on the solid-liquid contact angle.

Therefore, we can exclude the influence of the contact angle on the solid-liquid surfaces leading to the increase of electron transfer.

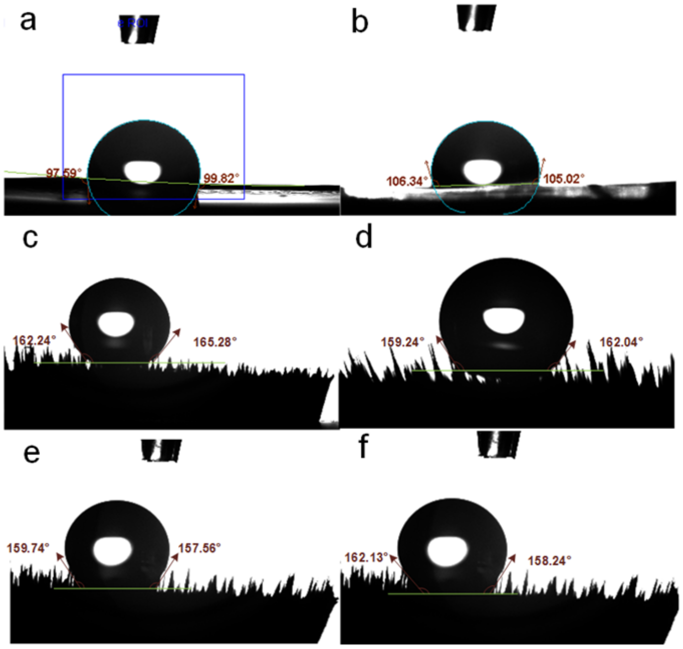

Fig. s2.1 Solid-liquid contact angle of different samples: (a) the contact angle of pure PDMS, (b) the contact angle of the PDMS doped with cobalt powder, (c) the contact angle of PDMS with bionic cilia unmodified TiO_2/PANI, (d) the contact angle of PDMS with bionic cilia modified TiO_2/PANI, (e) the contact angle of PDMS with bionic cilia modified TiO_2/PANI after illumination for 1 h, (f) the contact angle of PDMS with bionic cilia modified TiO_2/PANI after illumination for 3 h.

Response R2-F3: In order to confirm that the increase of electron transfer is induced by photocatalysis instead of photon excitation effect, we undergo the light effect experiments (open circuit voltage and short circuit current) during the process of light-dark-light state (Fig.s2.2). As a control group, light effect experiments of Pem-ITENG with PDMS film are carried out (Fig.s2.2a,b). The frequency of the reciprocating motion of the driving motor is set to 2 Hz. The open circuit voltage (about 15V) and short circuit current (700μA) of Pem-iTENG without bionic cilia unmodified TiO_2/PANI are not significantly changed in the light-dark-light state (see Fig.s2.2 a,b). Hence, it indicated that the increase of electron transfer is induced by photon excitation effect of the insulator surfaces can be eliminated.

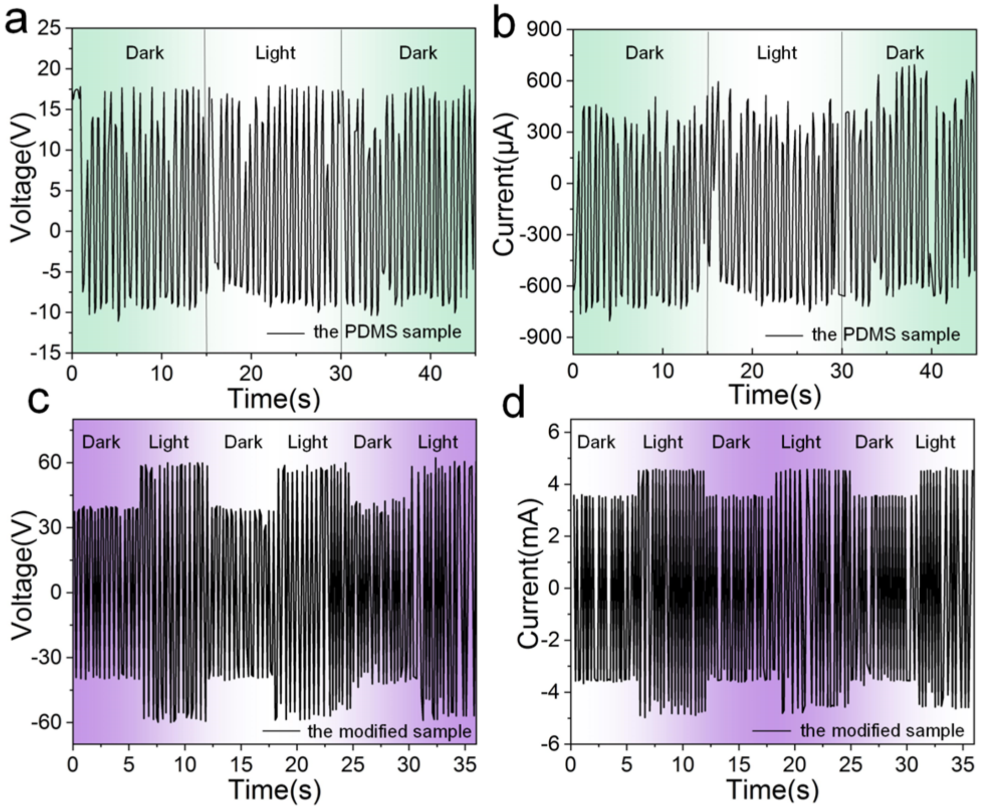

Fig.s2.2 The light effect experiments under light-darknes: (a) open circuit voltage and short circuit current (b) of PDMS without bionic cilia unmodified TiO_2/PANI, (c) open circuit voltage and short circuit current (d) of PDMS with bionic cilia modified TiO_2/PANI.

After the surface covered with bionic cilia modified TiO_2/PANI, the open circuit voltage and short circuit current of Pem-iTENG modified TiO_2/PANI are significantly improved in light compared with in dark. The open circuit voltage increases from 38.8 V in dark to 59.87 V in light, and the short circuit current increases from 2.4 mA in dark to 4.5 mA in light (see Fig.s2.2c,d). This indicated the enhancement of light

effect experiments of Pem-iTENG is attributed to the photocatalysis.

Therefore, compared with the improvement of the light effect experiments of Pem-iTENG with bionic cilia modified TiO_2/PANI in dark and light, we confirmed the increase of electron transfer is induced by photocatalysis after excluding the light excitation.

1. In the photocatalytic system (Fig. 1b), the authors claim that the holes tend to be retained in the VB of the TiO_2, while the electrons will transfer to cilia root. There are no evidences to support this explanation, because there is no field to separate the electron from the holes. Why the holes react with the water molecules and the impurities in liquid water, and the electrons don't?

Response: Thanks for your comments. Since our description of the separation of photogenerated electrons and holes in the earlier draft is relatively rough, we explain the separation of photogenerated electrons and holes in detail here.

When TiO_2 is modified by PANI, a type-II P-N heterojunction is formed at the interface of the TiO_2/PANI. Under the illumination of sunlight, TiO_2 and PANI primarily absorb UV and visible portions of the incident light, respectively. The photogenerated electrons and holes are separated by the built-in field (Advanced Energy Materials, 2020, 10(9): 1903713. ACS Applied Energy Materials, 2018, 1(5): 2087-2095. Small, 2016, 12(42): 5809-5816. ACS Applied Energy Materials, 2018, 1(5): 2087-2095.), and they move into TiO_2 (electrons) and PANI (holes), respectively (Fig.s2.3a,b).

When the Pem-iTENG is placed in a marine environment, the PANI covered at the surface of bionic cilia is exposed to moist water mist or organic matter, etc (ACS Nano, 2020, 14 (3): 3630-3639). The holes (h^+) at surface of the PANI will react with water molecules to form hydroxyl radical (Fig. s2.3(c)ii), which contributes to the oxidation and degradation of organic molecules (see Equation 2.1-2.4), while the excess photoelectrons will exist in TiO_2 due to the TiO_2 particles being wrapped between PANI and the bionic cilia (Fig. s2.3(c)i). Here, we believe that a few photoelectrons retained in PANI can react with oxygen to produce superoxide radicals, but the retained photoelectrons in PANI are very few, which can be ignored.

Therefore, through the built-in electric field of the P-N heterojunction, the effective separation of photogenerated electrons and holes can be realized.

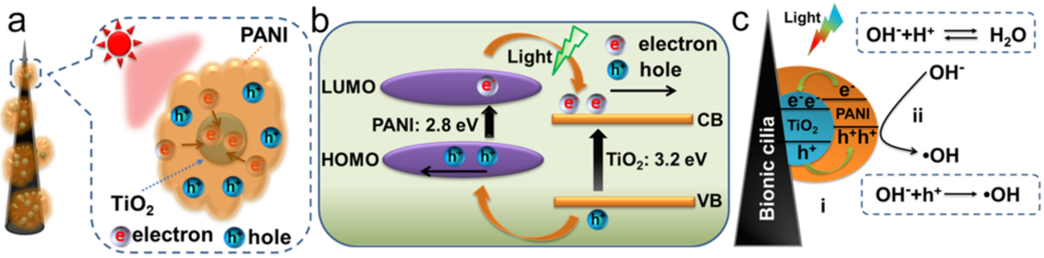

Fig. s2.3 Separation mechanism of photogenerated electrons and holes: a energy level conversion ofP-N heterojunction b schematic diagram of movement of photoelectrons and holes in the built-in field of P-N heterojunction. c charge transfer in the photocatalysis on single cilia surface.

$$\mathrm{PANI/TiO_2 + hv \rightarrow PANI^+ / TiO_2 + e^-_{CB}} \quad (2.1)$$

$$\mathrm{e^-_{CB} + O_2 \rightarrow O_2^-} \quad (2.2)$$

$$\mathrm{PANI^+/TiO_2 \rightarrow PANI/TiO_2 + h^+_{VB}} \quad (2.3)$$

$$\mathrm{h^+_{VB} + (H_2O \Leftrightarrow H^+ + OH^-) \rightarrow H^+ + OH^-} \quad (2.4)$$

2. I think the working principle of the Pem-iTENG is incorrect in Fig. 1d. Why the water is positively charged in the schematic? Here are two references for authors (Adv. Mater. 26 (2014) 4690; Nano Energy, 36 (2017), 250-259; Adv. Mater. Interfaces, 6 (2019), 1901547.), in which the principle of the liquid-solid TENG is described clearly and correctly.

Response: Thanks for your comments. The working principle of Pem-iTENG has been revised and explained as follows (Fig.1).

Specifically, a type II P-N heterojunction is formed at the interface of TiO_2 and PANI on the surface of bionic cilia. When the P-N heterojunction is illuminated by ultraciolet (UV) and visible portions of the incident sunlight, the effective separation of the photo-generated electrons (also called photoelectrons) and holes (Fig. 1b) can be realized by the built-in field of the P-N heterojunction (Advanced Energy Materials, 2020, 10(9): 1903713. ACS Applied Energy Materials, 2018, 1(5): 2087-2095. Small, 2016, 12(42): 5809-5816. ACS Applied Energy Materials, 2018, 1(5): 2087-2095.). Excess photoelectrons are retained in TiO_2 due to the TiO_2 particles being wrapped between PANI and bionic cilia. The photo-generated holes are transferred to PANI, then contact and react with moist seawater mist or organic impurities, etc. This causes the enhancement of the surface negative charge density of the PDMS film. Subsequently, through referring to the water wave motion generating the contact electrification (Nano Energy, 2019, 66: 104117. Advanced Energy Materials, 2017, 7(21): 1701243. Nano Energy, 2014, 9: 186-195.), we propose the working principle of Pem-iTENG under the synergy of the contact electrification and the photocatalysis

in a cycle of reciprocating tidal wave (see Fig. 1c).

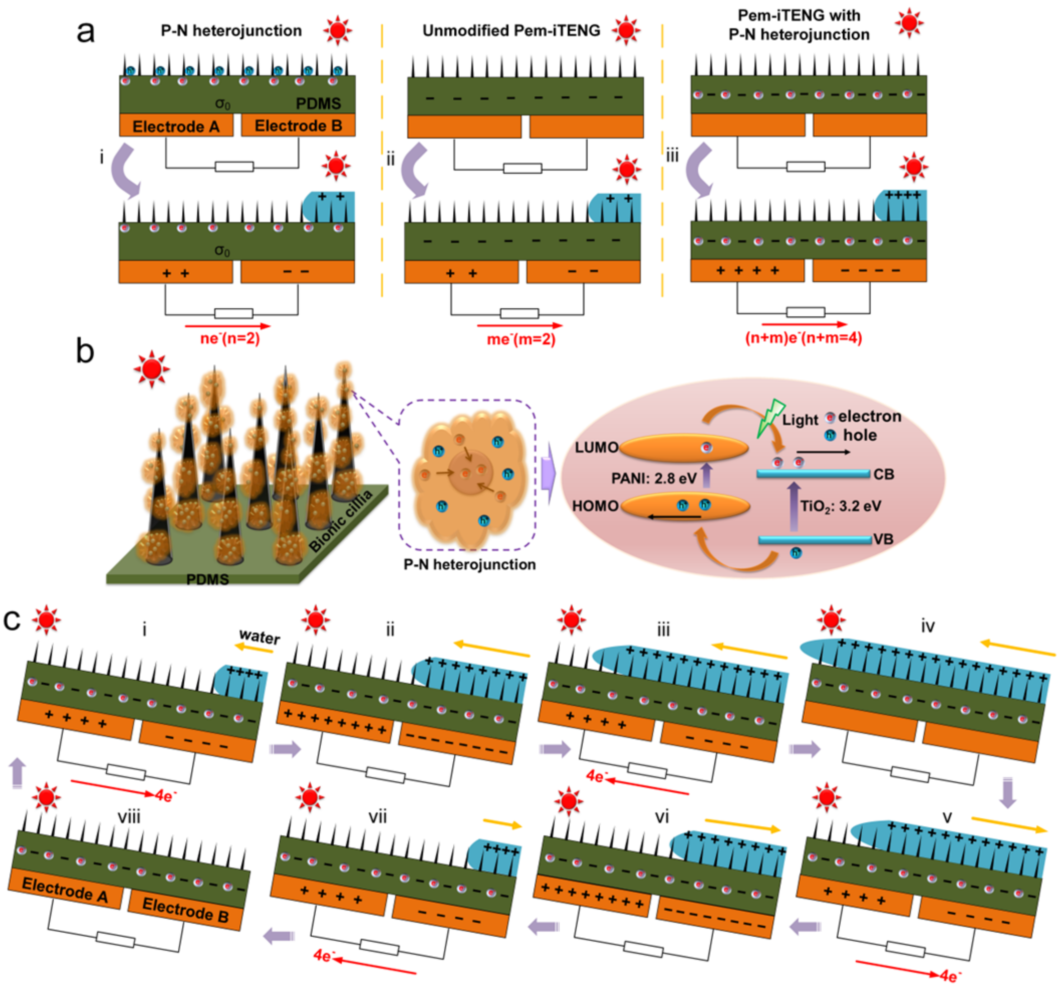

Fig. 1 the working principles of Pem-iTENG. (a) Pem-iTENG with P-N heterojunction (i), unmodified and modified Pem-iTENG during the two flow states of the tidal waves (ii and iii). (b) principles of photocatalysis of bionic cilia with P-N heterojunction. TiO_2 (bandgap 3.2 eV) can only absorb ultraviolet light, while PANI (bandgap 2.8 eV) can absorb both ultraviolet and visible light (c) the contact electrification of Pem-iTENG in a cycle of reciprocating tidal wave, and free electrons transfer direction between the two electrodes.

Once water waves immerse on the surface of the PDMS film with bionic cilia, the rising contact seawater forms an electric double layer with the PDMS film to screen the negative charges on the PDMS film partly. Due to the asymmetric distribution of charges, the unbalanced potential between the two electrodes drives free electrons to flow out from one electrode to the other, and forms the alternating current through the external circuit (see Fig. 1(c)i,iii,v,vii). When the tide wave rises or falls to the gap between the two electrodes, there is no electron transfer (see Fig. 1(c)ii,vi,). When the tidal wave rises to the top (see Fig. 1(c)iv), the potential difference between the two

electrodes decays to zero without electron transfer due to the symmetrical screening of the electric double layer. During the first rise of the water wave, the PDMS film keeps accumulating the triboelectric negative charges and photoelectrons without dissipating in a period time, and there is no electron transfer between the two electrodes (see Fig. 1(c)viii).

Based on the relevant information provided by the reviewers, we believe that the principle of the solid-liquid triboelectric nano-generator can be explained in two ways. One of the working model is based on the fact that liquid droplets constantly strike on the surface of the friction polymer film (Fig.s2.4), and the contact electrification phenomenon occurs (Nano Energy, 36 (2017), 250-259. Advanced Materials Interfaces, 2019, 6(24): 1901547.). Another solid-liquid contact model is that the water flow continuously washes the friction polymer film by the principle of electrostatic induction to collect electric charges (Fig.s2.5), and an alternating current is generated (Nano Energy (2014) 9, 186-195). In fact, the working principle of the power generation driven by liquid droplets or water waves is similar, and the output electrical performance is both realized by contact electrification. Our Pem-iTENG to realize the conversion of water wave energy to electric energy is more inclined to Yuanjie Su, et al. proposal of the collection charges by the electrostatic induction principle under the water flow (Fig.s2.5).

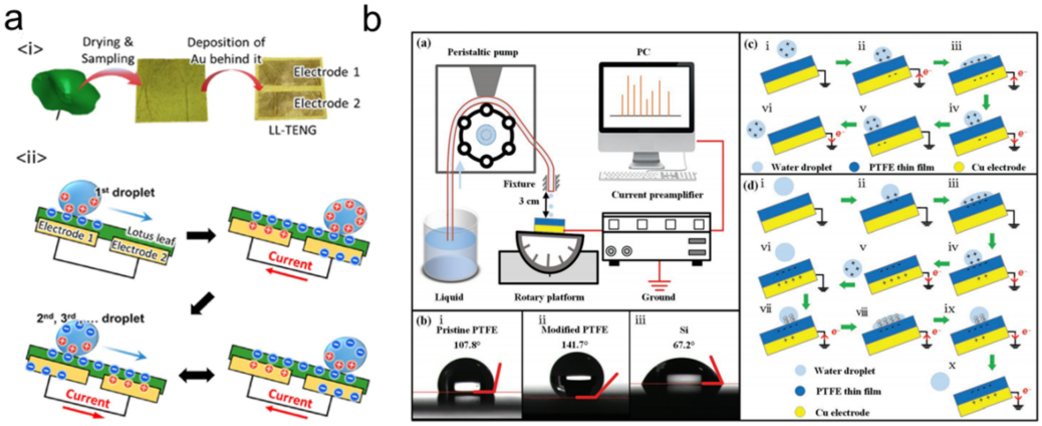

Fig. s2.4 schematic diagram of the natural lotus leaf-TENG proposed by Choi *et al* and Yang *et al*.

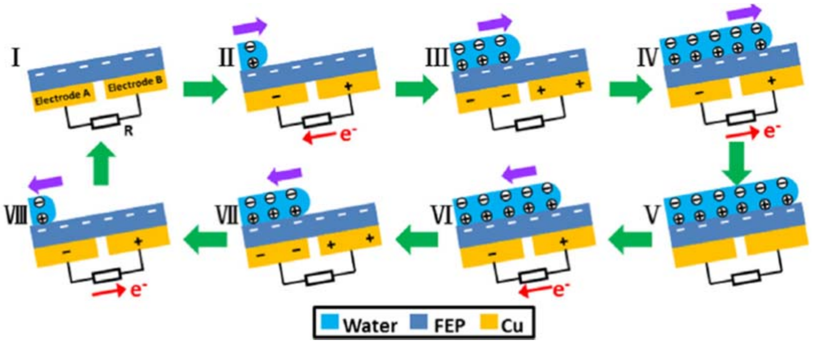

Fig. s2.5 Working principle of interfacial electrification enabled TENG by Yuanjie Su et al.

In addition, it is worth noting that seawater remains electrically neutral during the entire contact electrification. As known, the mechanism of triboelectrification is the formation of chemical bond at the contact position of two materials. Therefore, the formation of friction charge depends on the electrode difference of friction composite material itself. When two materials are in contact, the surface of the materials easy to

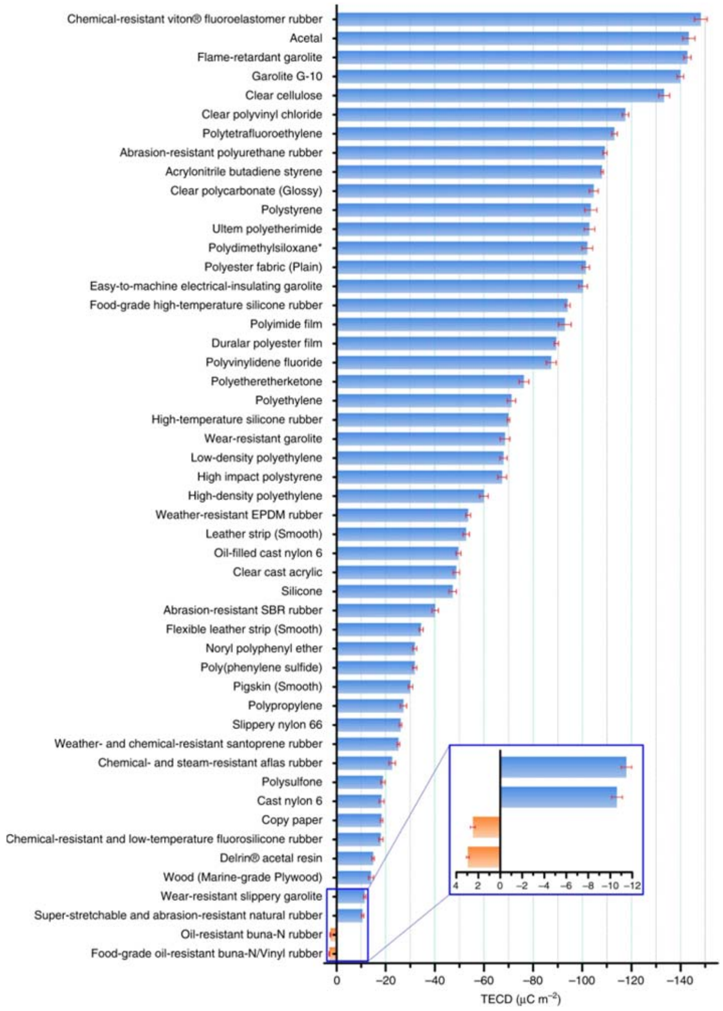

Fig. s2.6 The quantified triboelectric series (Nat. Commun. (2019) 10, 1427).

obtain electrons is negatively charged, while the surface of materials easy to lose electrons is positively charged (the electrostatic sequence table of common materials is shown in Fig. s2.6). In our research work, when the PDMS friction film contacts with seawater, due to the difference of the electron gain and loss ability between PDMS and seawater, the surface of PDMS is easy to obtain electrons and carries negative charges. These charges will not dissipate for a long time. Due to electrostatic induction, seawater carries positive charge. In fact, the electrical neutrality of seawater remains unchanged, but it shows a positive charge accumulation at the contact surface.

3. The Pem-iTENG is inspired by the lotus leaf, to obtain the advantages of hydrophobicity, self-cleaning and surface roughness. However, the effects of the hydrophilia and roughness of the TENG on the output performance are not discussed.

Response: Thanks for your comments. First of all, we focus on the photoelectric-mechanical integrated high-efficiency energy conversion technology in this work. In order to explain this problem, we analyze the characteristics of charge density on hydrophobic surfaces in Question 4. At the same time, it is necessary to say that the influence of hydrophobicity of solid-liquid triboelectric nano-generators on their performance has been reported as follows.

Lin *et al.* reported that in order to obtain the maximum electrical output, the previous water drop should be completely removed from the surface of the triboelectric nano-generators before the next drop falls on the triboelectric nano-generator, which requires super-hydrophobicity surface (Advanced Materials, 2014, 26(27):4690-4696). The current increases with the speed of water drop sliding. The super-hydrophobic property of triboelectric nano-generator also changes the surface water content. The hydrophilic nylon film and the untreated hydrophobic PTFE film with smooth surface were used to replace the original super-hydrophobic PTFE film, and the output current density decreased significantly (Fig. s2.7). This shows that the solid-liquid contact triboelectric nano-generator has the importance of super-hydrophobicity, and the hydrophobicity of the film is a key factor in the performance of the solid-liquid contact triboelectric nano-generator. In fact, we know that improving the surface roughness of friction film can improve the output

performance of solid-liquid contact triboelectric nano-generators, but the relationship between surface roughness and static contact angle is not simple linear. When the surface roughness exceeds a certain range, the surface roughness increases and the static contact angle of the materials decreases, which makes the material surface lose the super-hydrophobic property. This is contrary to the design and fabrication of super-hydrophobic triboelectric nano-generator with solid-liquid contact.

Zhou *et al.* proved that the super-hydrophobic surface can improve the stability of TENG (Nano Energy, 2019, 57: 903-910). Fig. s2.8a shows that with the increase of RH, the output voltage of triboelectric nano-generators decreases. However, when the relative humidity is as high as 80%, the output voltage of super-hydrophobic PDMS's TENG is much higher than that of ordinary PDMS. When TENG is wetted by water, the recovery time of its output performance (see Fig. 2.8b and 2.8c) shows that it takes 66.2 s for TENG of ordinary PDMS to recover its output performance from water wetting, while only 24.6s is needed for super-hydrophobic PDMS's TENG under the same conditions. In addition, due to the self-cleaning characteristics of super-hydrophobic surface, the dust on the TENG can be easily washed off to keep the surface clean, thus prolonging the service life of TENG.

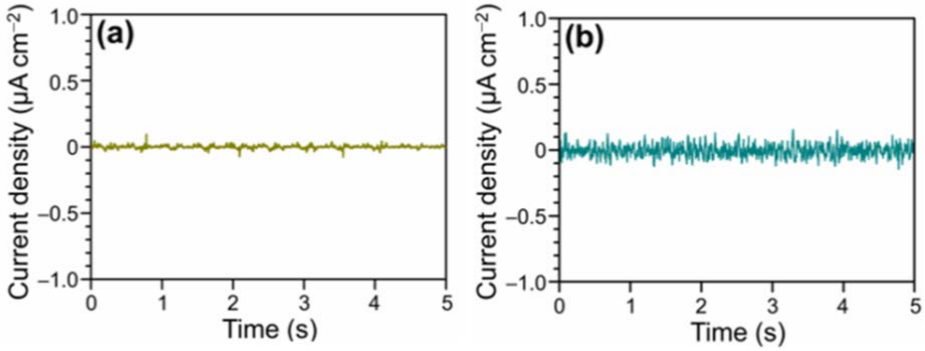

Fig. s2.7 Output current density of water-TENG composed of hydrophilic material nylonfilm (a) and hydrophobic PTFE film (b) with smooth surface

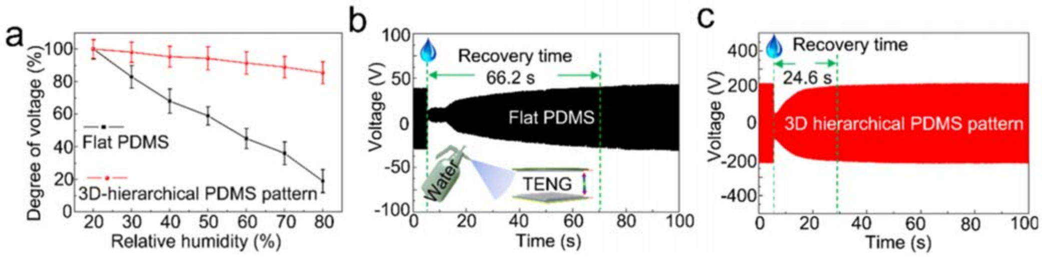

Fig.s2.8 (a) RH effects upon the output voltage of the TENGs with a flat PDMS interlayer and a 3D hierarchical PDMS interlayer. (b) recovery time of the output signal after the TENGs with a flat PDMS interlayer, (c) 3D hierarchical PDMS interlayer dampened by spraying of water twice.

Secondly, the effect of surface roughness difference on the output performance of solid-liquid contact triboelectric nano-generators has been proposed in this paper, but we define it as the cilia's aspect ratio of photoelectric electromechanical integrated composite membrane (PEM-iCM). Moreover, the surface roughness is basically unchanged in the same length of bionic cilia modified or unmodified TiO2/PANI. The effect of the surface roughness of bionic cilia formed by the TiO2/PANI is excluded during the seawater reciprocating scouring (see Fig.s2.1 and Fig.s2.9). The electrical properties of the composite friction film prepared by magnetic adsorption of three kinds of cobalt powder (300nm, 800nm, 1300nm) were tested in the text of the manuscript. The results show that the sample with the particle size of 800 nm has the best electrical properties, so this surface roughness of the sample is the most suitable for solid-liquid contact triboelectric nano-generator.

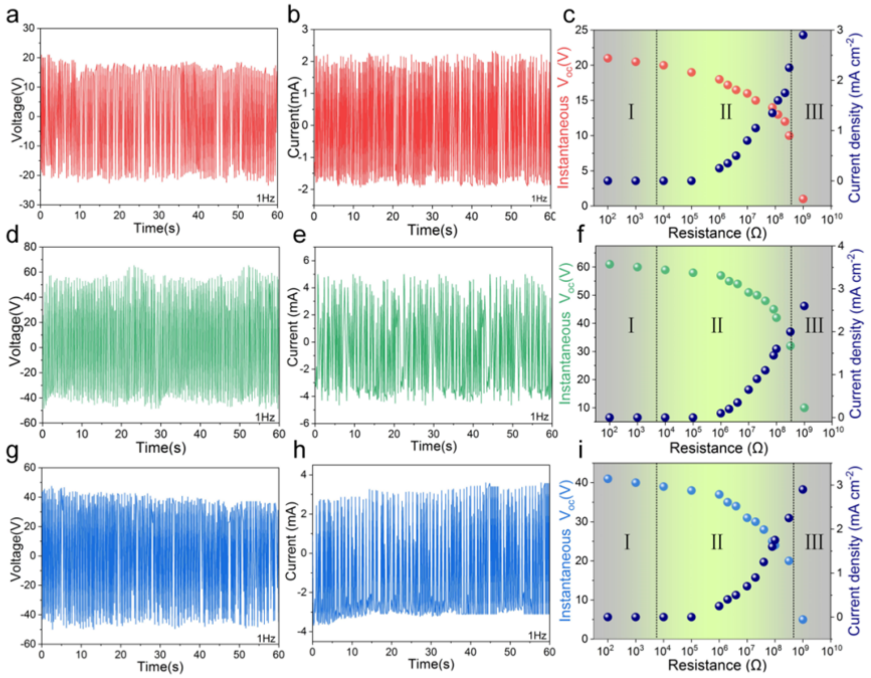

Fig. 4 a, d, g are the open circuit voltage at 2 Hz solid-liquid contact frequency of three kinds of high width ratio fiber wool samples; **b, e, h** are the short-circuit current of three kinds of high width ratio fiber samples with solid-liquid contact frequency of 2 Hz; **c, f, i** are the change diagram of output voltage and output current of solid-liquid contact Pem-iTENG with external resistance between 10^2 Ω and 10^9 Ω.

In order to improve the surface charge density, the method of changing the particle size of cobalt powder (300nm, 800nm, 1300 nm) was used to prepare PEM-iCM samples with various aspect ratio of cilia, so as to improve the surface contact electrification state. Fig. 4 shows the electrical properties of Pem-iTENG samples

with three aspect ratios at the water wave frequency of 2 Hz. The results show that the output voltage of triboelectric nano-generator can be increased by the microstructure of 300 nm, 800 nm and 1300 nm. The self-growing bionic cilia of 800 nm magnetic cobalt powder can improve the output voltage most obviously. The maximum open circuit voltage is 64.2 V (see Fig.4d) and the maximum short-circuit current is about 5.1 mA (see Fig.4e), which is much higher than the previous solid-liquid contact TENGs such as linear, cubic, pyramid and other microstructures.

4. It is suggested that the surface charge density of the samples in the experiments should be measured or calculated.

Response: The supplementary experiment of the charge density is performed during the fluctuation simulation test. And the charge density of Pem-iTENG with bionic cilia unmodified or modified TiO_2/PANI is tested before and after the illumination (see Fig. s2.9). The metal copper is the fixed electrodes (see Fig.s2.9a), and the output end of the electrode is connected to the test equipment through a rectifier bridge. The surface structure of single cilia indicted that the surface of bionic cilia is wrapped with a layer of modified TiO_2/PANI (see Fig.s2.9b).

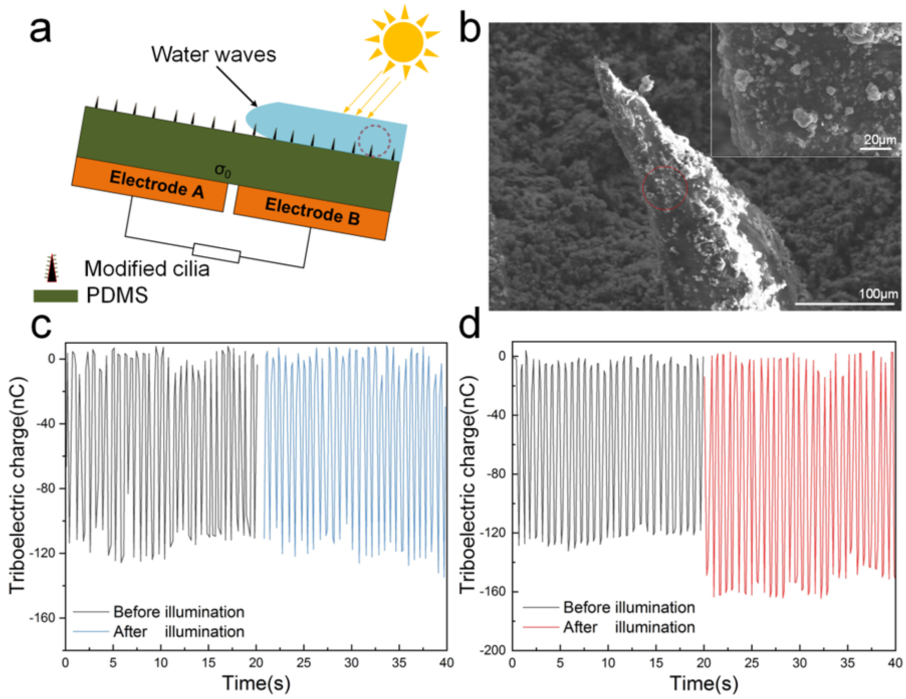

Fig.s2.9 Charge density diagram under different experimental conditions: (a) simple structure diagram of Pem-iTENG, (b) single bionic cilia modified TiO_2/PANI, (c) charge density

unmodified TiO_2/PANI before and after the illumination, (d) charge density modified TiO_2/PANI before and after the illumination.

The charge density of Pem-iTENG with bionic cilia unmodified TiO_2/PANI remains at 122.38 nC in dark and light (see Fig.s2.9c). This indicated that sunlight cannot enhance the charge density of Pem-iTENG after the illumination. The charge density of Pem-iTENG with bionic cilia modified TiO_2/PANI increases from 122.73 nC to 164.12 nC in the dark and light (see Fig. s2.9d), and it revealed that after modified with TiO_2/PANI, sunlight can enhance the charge density of Pem-iTENG. It is worth noting that compared with unmodified TiO_2/PANI in dark, the charge density of Pem-iTENG modified TiO_2/PANI in dark is very close. After eliminating the influence of sunlight and the surface hydrophobic properties (Fig.s2.1), it indicated that surface roughness has basically no effect on the charge density before and after modification.

Therefore, through the systematic experimental test results of charge density as well as excluding the increase of electron transfer is induced by photocatalysis instead of photon excitation effect (see Fig.s2.2), we demonstrate that the working mechanism of the proposed Pem-iTENG are correct, and our Pem-iTENG can achieve the photoelectric-mechanical integrated high-efficiency energy conversion.

5. The academic term for “friction electrification” is “triboelectrification” or “contact electrification”.

Response: Thanks for your comments. We have carefully checked the differences and made corrections, which is highlighted in red.

Reviewer #3:

The authors developed a bio-inspired solid-liquid interface contact-based photoelectric-electromechanical integrated triboelectric nano-generator. The energy conversion efficiency can reach 67.4%. The authors contributed this high efficiency to the efficient sunlight capture by PN heterojunction. The topic is interesting, but it seems that there are many experimental results/proposed mechanism which needs carefully being addressed before publication.

1. The diagrams of energy level alignment between PANI and TiO_2 in Figure 1b and c are contradictory. In figure 1b, the interface seems like a type Ⅰ heterojuction. But in the figure 1c, the energy alignment of the heterojunction is type Ⅱ. Additionally, I not sure that it is suitable to descript this interface by P-N junction? Polyanine is conducting polymer instead of semiconductor one. And in figure 1c, the bandgap values between PANI and TiO_2 are different. But in the picture the distances between CB and VB of two different materials are almost the same. It is contradictory.

Response: Thanks for your comments. We carefully checked the problem of the contradiction of the energy level arrangement between PANI and TiO_2 (see revised Fig. 1(a)), and the band gap size of PANI has been corrected in the manuscript.

For the problem of P-N junction, we know that the interface heterojunction can be classified into three types of type-I, type-II and type-III, while Z-type photoreaction system includes the photoreaction system i and the photoreaction system ii (Chemical Communications, 2001, 23 (1): 2416-2427). In the process of Z-type catalytic reaction, photogenerated electrons (also called photo electrons) and holes are generated after photoexcitation for the photoreaction system i and ii. Electrons in photoreaction system ii are oxidized by oxidation-reduction couples, while electrons in photoreaction system i are reduced by oxidation-reduction couples. In this way, more photoelectrons on the conduction band of photoreaction system i and holes on the valence band of photoreaction system ii can be reserved (see Fig. 3.1). Therefore, the symbol marks in the article made the reviewers misunderstand. Actually, the interface formed by PANI/ TiO_2 described in Figs. 1(b,c) (see revised Fig. 1(a)) in this paper is the same type, that is, the interface of P-N heterojunction is type-II heterojunction. In

order to make it clear, we have modified the symbols in the article.

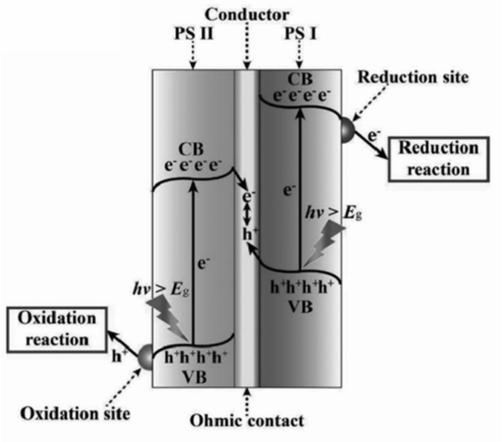

Fig. s3.1 PS-C-PS type Z photocatalytic reaction mechanism.

In view of this interface by P-N junction proposed by the reviewers, the heterojunction interface formed by semiconductor of TiO_2 and conductive polymerization of PANI on cilia surface in this paper can be called P-N heterojunction, but it is different from the traditional semiconductor heterojunction. **The detailed explanation about P-N heterojunction is as follows.**

Firstly, many N-type oxides with semiconductors characteristics have been proposed, such as titanium dioxide, tin oxide, zinc oxide and other semiconductor metal oxides. However, few metal oxides with good visible/ ultraviolet (UV) photosensitivity have been reported. There are few kinds of nanomaterials with photosensitivity. Even if some nanomaterials show photosensitivity, there is still much room for improvement in their photosensitivity. After modifying the surface of metal oxide with conductive polymerization, the photosensitivity of these nanomaterials can be improved, or the sensitive range to light wave can be widened, which make the photosensitivity enhance to some extent. For example, Mojtabavi et al. developed a flexible self-powered photodetectors (PDs) with multi-color detection capability (Applied Surface Science, 2019, 492: 189-198). Polyaniline/Titanium dioxide (PTP) and Titanium dioxide/Polyaniline (TPP) heterojunctions were deposited on a flexible PET substrate. Under UV and visible light irradiation, the I-V curves of PTP and TPP show different contrast ratios, rectification ratios, effective barriers height and reverse saturation currents, which indicates that the device can be used as an effective two-color PDs without an external power source. At the same time, Zhonglin Wang's team made a zinc oxide nano-sensor, whose surface was modified by sodium polystyrene sulfonate (PSS), and experiments showed that the absorption capacity of

the sensor for ultraviolet light was greatly enhanced, and the photosensitivity was increased by five orders of magnitude (Journal of the American Chemical Society 129.40 (2007): 12096-12097).

Secondly, conductive polymerization is widely used in low-cost photosensitive devices, mainly because their internal electrons exist in the form of ring π, so they have strong light absorption ability. Metal oxides are often modified by conductive polymerization with some excellent properties. Taking titanium dioxide (TiO_2) as an example, conductive polymerization can broaden the photosensitive wavelength range of TiO_2, improve the photosensitivity, and accelerate the separation rate of photogenerated electrons and photogenerated holes. Many conductive polymerizations can be used to modify semiconductor metal oxides, such as sodium polystyrene sulfonate (PSS), poly dimethyl diallyl ammonium chloride (PDADMAC or PDDA), polyaniline (PANI), poly pyrrole (PPY) and some dyes. For example, F. F. Amos et al. fabricated photosensitive detectors by adsorbing cadmium sulfide semiconductor nano-arrays on elastic PET substrates in aqueous solution. They created another membrane with composite structure, which was prepared by using LBL technology to adsorb titanium dioxide nanosheets, PEI and PDDA on conductive ITO substrate (Superlattices and Microstructures, 2010, 48 (2): 170-180). As we all know, ions in semiconductor metal oxides are stable and immobile, so the conduction between ions can be neglected. These ions are in contact with each other between the N-type semiconductor and the P-type conductive polymerization, and the interface contact area between them forms a depletion area composed of charged ions, which is the P-N junction, as shown in Fig.s3.2.

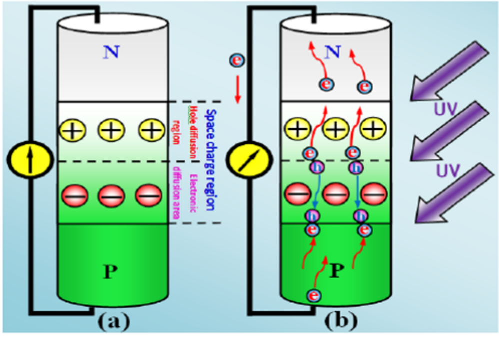

Figure s3.2 Schematic diagram of the working mechanism of P-N heterojunction.

Therefore, the heterojunction interface formed by semiconductor of TiO_2 and conductive polymerization of PANI on cilia surface in this paper can be called P-N heterojunction, but it is different from the traditional semiconductor heterojunction.

The traditional semiconductor heterojunction is a special kind of P-N junction, which is formed by depositing more than two layers of different semiconductor materials on the same pedestal in turn with different energy band gaps.

For the problem of the bandgap values between PANI and TiO_2 in Fig. 1c (the distances between conduction band (CB) and valence band (VB) of two different materials are almost the same), we have revised the distances between CB and VB in the full paper (see revised Fig. 1(a)).

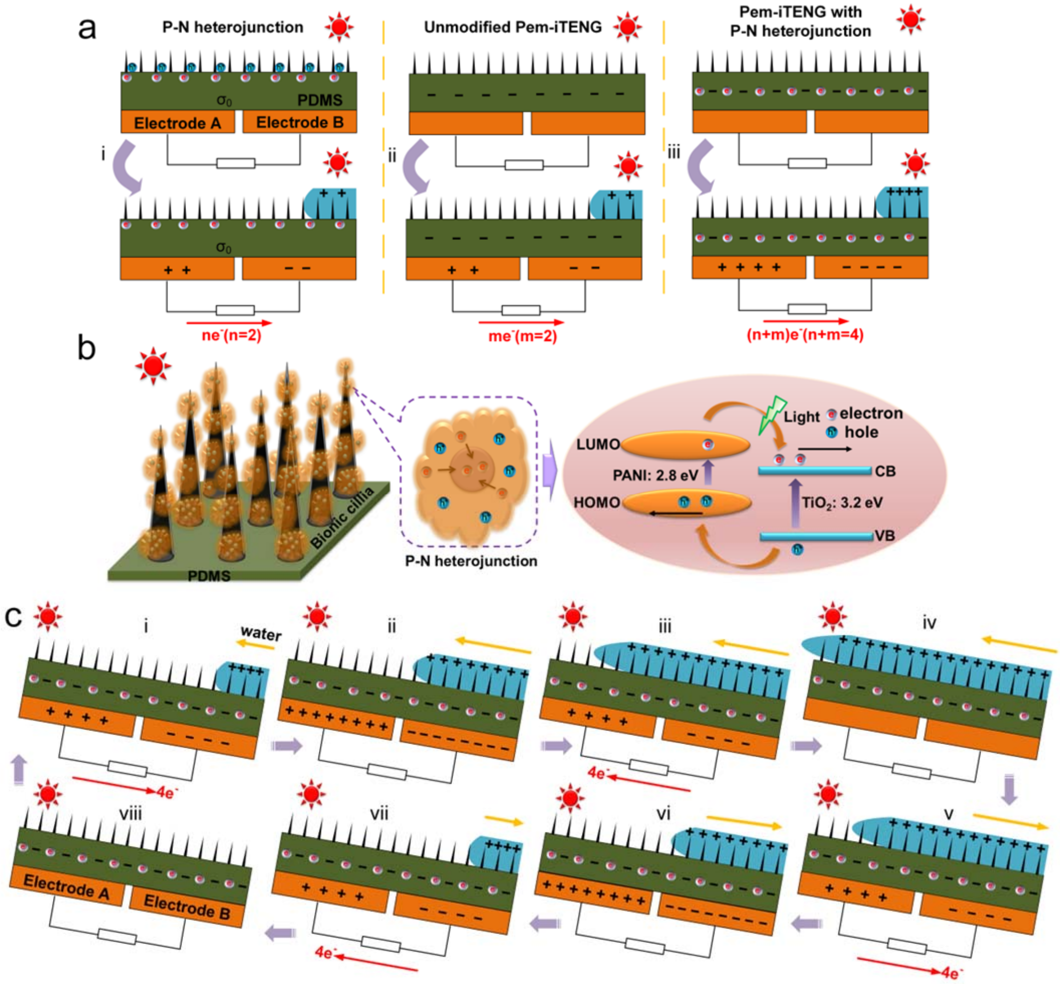

Fig. 1 the working principles of Pem-iTENG. (a) Pem-iTENG with P-N heterojunction (i), unmodified and modified Pem-iTENG during the two flow states of the tidal waves (ii and iii). (b) principles of photocatalysis of bionic cilia with P-N heterojunction. TiO_2 (bandgap 3.2 eV) can only absorb ultraviolet light, while PANI (bandgap 2.8 eV) can absorb both ultraviolet and visible light (c) the contact electrification of Pem-iTENG in a cycle of reciprocating tidal wave, and free electrons transfer direction between the two electrodes.

2. There are less evidences to show that the efficient charge separation takes place between PANI and TiO_2. Such as surface potential difference, photovoltaic effect. Assuming that the carrier separation can take place at the interface, is it high likely that the recombination happens during operation since the seawater can work as a conductor?

Response: Thanks for your comments. Since our description of the photogenerated electrons and holes in the earlier draft is relatively rough, we provide some evidences below to explain the cause of the efficient separation and recombination of charges in our Pem-iTENG.

When TiO_2 is modified by PANI, a type-II P-N heterojunction is formed at the interface of the TiO_2/PANI. Under the illumination of sunlight, TiO_2 and PANI primarily absorb UV and visible portions of the incident light, respectively. The photogenerated electrons and holes are separated by the built-in field (Advanced Energy Materials, 2020, 10(9): 1903713. ACS Applied Energy Materials, 2018, 1(5): 2087-2095. Small, 2016, 12(42): 5809-5816. ACS Applied Energy Materials, 2018, 1(5): 2087-2095.), and they moved into TiO_2 (electrons) and PANI (holes), respectively (Fig. s3.3a,s3.3b).

When the Pem-iTENG is placed in a marine environment, the PANI covered at the surface of the bionic cilia is exposed to moist water mist or organic matter, etc (ACS Nano, 2020, 14 (3): 3630-3639). The holes (h^+) at surface of the PANI will react with water molecules to form hydroxyl radical (Fig. s3.3c ii), which contributes to the oxidation and degradation of organic molecules (see Equation 3.1-3.4), while the excess photoelectrons will exist in TiO_2 due to the TiO_2 particles being wrapped between PANI and the bionic cilia (Fig. Fig. s3.3c i). Here, we believe that a few photoelectrons retained in PANI can react with oxygen to produce superoxide radicals, but the retained photoelectrons in PANI are very few, which can be ignored.

Therefore, through the built-in electric field of the P-N heterojunction, the effective separation of photogenerated electrons and holes can be realized.

Following the above process, since the photoelectrons have no hole to pair and the PDMS film is a tribopolymer that easily absorbs negative charges without dissipating in a period of time, the photoelectrons are retained in the contact interface of bionic cilia and TiO_2, which increase the surface charge density of the PDMS film. Hence, when the tidal water wave rises, the contact seawater as a conductor and the PDMS

film construct an electric double layer to screen the negative charges on the PDMS film partly (Nano Energy, 2014, 9: 186-195), which promotes the photoelectrons induced positive charges in seawater. This coupling the physical effect of contact electrification and electrostatic induction is proposed very early and is also the theoretical support of the current triboelectric nanogenerators (Nano Energy, 2014, 9: 186-195. Nature communications, 2017, 8(1): 1-7. Nature, 2020, 578(7795): 392-396).

Therefore, since seawater as a conductor can trigger the contact electrification, the charge induction between photoelectrons (excess) and positive charges (seawater) can occur in the electric double layer while the **r**ecombination of photo-generated electrons and holes cannot happen again.

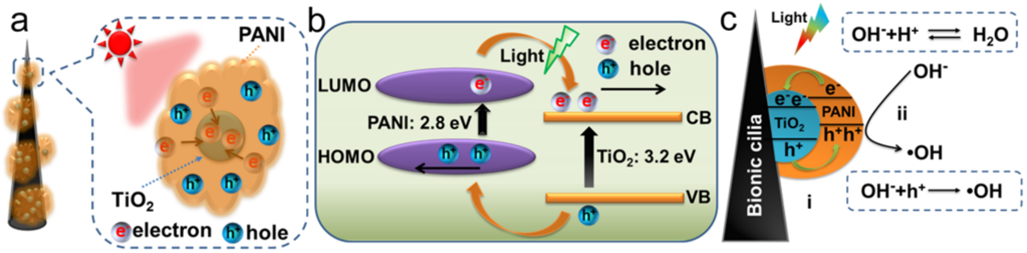

Fig. s3.3 Separation mechanism of photogenerated electrons and holes: a energy level conversion ofP-N heterojunction b schematic diagram of movement of photoelectrons and holes in the built-in field of P-N heterojunction. c charge transfer in the photocatalysis on single cilia surface.

$$\mathrm{PANI/TiO_2 + hv \rightarrow PANI^+ / TiO_2 + e^-_{CB}} \quad (3.1)$$

$$\mathrm{e^-_{CB} + O_2 \rightarrow O_2^-} \quad (3.2)$$

$$\mathrm{PANI^+/TiO_2 \rightarrow PANI/TiO_2 + h^+_{VB}} \quad (3.3)$$

$$\mathrm{h^+_{VB} + (H_2O \Leftrightarrow H^+ + OH^-) \rightarrow H^+ + OH^-} \quad (3.4)$$

3. The calculation of the “average power density” in method part is confusing. What dose the “average” mean? Since the V(t) and I(t) are the functions of the time, is it a time average or something else? Why did the authors use the peak voltage and peak current to conduct the calculation?

Response: Thanks for your comments. Because the output power of TENG has a certain relationship with the size of friction composite film. In this work, in order to describe the output power density of Pem-iTENG per unit area more properly, we calculate the output power per unit area of Pem-iTENG under different loads by using the ratio between the power density and the area of friction composites film, namely P_{avg}, unit mW/cm^2. In order to avoid ambiguity, the concept of "average power density" has been modified in the manuscript. "average" is removed in the paper, and

"power density" is used to represent the output power density per unit area of Pem-iTENG under different loads.

In order to study the output power density of Pem-iTENG, we tested the output voltage and current when connected to external load resistance. The traditional power spectral density spectrum is a probabilistic statistical method, which is a measure of the mean square value of random variables and is generally used for random vibration analysis. The continuous transient response can only be described by probability distribution function, that is, the probability corresponding to a certain level of response. The power spectral density of a signal exists only if the signal is a generalized stationary process. If the signal is not a stationary process, the autocorrelation function must be a function of two variables, so there is no power density spectral, but similar techniques can be used to estimate the time-varying density spectral. Therefore, in order to simplify the calculation process, we use the peak voltage and peak current to represent the output power density under different loads.

4. Following the aforementioned question, it is highly suspected that the energy efficiency calculation is proper used. The authors claims that the power conversion efficiency is as high as 67%. It is a very high value. Here, how do the author define the power conversion efficiency? It means the ratio of electrical power output dividing water mechanical energy as well as adding up the solar (light) illumination? I strongly suggest that the authors refer to the solar cell power conversion efficiency, it is widely accepted and applied both in industry and academy.

Response: Thanks for your comments. Compared with the photovoltaic cells, the energy conversion rate of triboelectric nano-generator is really high, but this does not mean that they have good or bad performance comparison through calculation results, because the energy conversion principles and calculation methods of the two devices are different. The energy conversion rate of solar cells can be calculated by J-V curve, but triboelectric nano-generator is mainly calculated by the unstable alternating current generated by contact electrification. In this paper, Pem-iTENG obtains mechanical energy from the solid-liquid friction process of tide wave scouring, while light only plays an auxiliary photocatalysis role in P-N heterojunction. Even if it can generate electricity after light illumination, its power generation is far less than the

magnitude of mechanical triboelectric energy, which can be basically ignored. Therefore, the calculation method of the power conversion efficiency of solar cells is not suitable for the energy conversion efficiency of Pem-iTENG proposed in this paper.

The energy conversion rate of TENG refers to the calculation method in the literature (Nature, 2020, 578(7795): 392-396), and the ratio of input energy to output energy is often used to evaluate. For example, Xu et al. developed a droplet power generation device, which uses a structure to collect energy from the impacted water droplets. In order to calculate the conversion efficiency from mechanical energy to electrical energy, the calculation method of the conversion efficiency is defined when the 1th droplet strike.

$$\eta_i = \frac{E_{out}}{E_{in}} = \frac{\int_{t_{on}}^{t_{off}} \frac{U^2}{R_L} dt}{mgh} \quad (3.5)$$

Whereby, m is the mass of the droplet which is 0.1g, g is the gravitational acceleration which is 9.8 m $s^{-2,}$ and h is the relative height of the released droplet and water which is 15.0cm. R_L is 10.0 MΩ.

Based on the above calculation method, the energy conversion rate (ECR) of Pem-iTENG can be expressed by the ratio of the output electric energy under different external load resistors to the total input energy (the sum of mechanical energy and solar energy), which can be expressed as follows:

$$\begin{aligned} ECR(\%) &= \frac{E_{out}}{E_{me}+E_{op}} \\ &= \frac{\int_{t_1}^{t_2} I^2 R dt}{\frac{1}{2}mv^2 + Pt \cdot \eta} \end{aligned} \quad (3.6)$$

Whereby, E_{out} is the output electrical energy, E_{me} is the input mechanical energy, E_{op} is the input optical energy, m is the mass of Pem-iCM, v is the speed of linear motor, P is the power of xenon lamp, and η is the utility rate of luminous energy.

It is worth noting that η in the formula refers to the utilization rate of light energy, that is, the electric energy consumed by solar energy/xenon lamp received by Pem-iCM with a value of 1.5% (J. Mater. Chem. 4, 2016, 10395). Light energy is the driving force of Pem-iCM in photocatalysis. The utilization rate of light energy generally refers to the ratio between the energy contained in organic matter produced

by crops through photosynthesis and the solar energy received by this land per unit land area. The theoretical calculation value can generally reach 6.0% ~ 8.0%, but only 0.5% ~ 1.0% in actual production, and up to 2% at most. Because Pem-iTENG is inspired by the photocatalytic principle of green plants, only part of the frequency of solar energy irradiated on Pem-iCM can be absorbed and utilized by Pem-iTENG. At the same time, solar energy irradiated on the surface is not completely absorbed, part of it is lost into the air due to reflection, and part of it is not absorbed because it passes through the friction composite film. Most of the solar energy absorbed by the friction composite film is converted into heat energy, and only a small part of the energy is utilized by photocatalysis. Therefore, the input light energy cannot be simply considered as the power consumption of xenon lamp. Here, we define a conversion parameter. That is, the utilization rate of light energy follows the concept of leaf in the photocatalysis, but it is not the same as the photoelectric conversion efficiency in the photovoltaic cells.

5. The authors claim that there are charge trsanfer/exciton dissociation at the interface between PANI and TiO_2. However, there is no experiment data either external quantum efficiency or transient optical measurement. Since it is the basic point of this manuscript, I would suggest that authors need to provide solid evidence to verify this point.

Response: At present, it is difficult to directly test charge trsanfer/exciton dissociation at the interface between PANI and TiO_2. Here, we explained this problem from the perspective of system experiments in response to this problem.

Firstly, the supplementary experiment of the charge density is performed during the fluctuation simulation test. And the charge density of Pem-iTENG with bionic cilia unmodified or modified TiO_2/PANI is tested before and after the illumination (see Fig. s3.5). The metal copper is the fixed electrodes (see Fig.s3.5a), and the output end of the electrode is connected to the test equipment through a rectifier bridge. The surface structure of single cilia indicted that the surface of bionic cilia is wrapped with a layer of modified TiO_2/PANI (see Fig.s3.5b).

The charge density of Pem-iTENG with bionic cilia unmodified TiO_2/PANI remains at 122.38 nC in dark and light (see Fig.s3.5c). This indicates that sunlight cannot enhance the charge density of Pem-iTENG after the illumination. The charge density of Pem-iTENG with bionic cilia modified TiO_2/PANI increases from 122.73

nC to 164.12 nC in the dark and light (see Fig. s3.5d), and it reveals that after modified with TiO_2/PANI, sunlight can enhance the charge density of Pem-iTENG. It is worth noting that compared with unmodified TiO_2/PANI in dark, the charge density of Pem-iTENG modified TiO_2/PANI in dark is very close. After eliminating the influence of sunlight, it indicates that factors of surface roughness, etc. have basically no effect on the charge density before and after modification with TiO_2/PANI. Therefore, we demonstrated that sunlight can excite a significant change in the charge density of our Pem-iTENG modified TiO_2/PANI during the fluctuation simulation test.

Secondly, we undergo the light effect experiments (open circuit voltage and short circuit current) during the process of light-dark-light state (Fig.s3.6). As a control group, light effect experiments of Pem-ITENG with PDMS film are carried out (Fig.s3.6a,b). The frequency of the reciprocating motion of the driving motor is set to 2 Hz. The open circuit voltage (about 15V) and short circuit current (700μA) of Pem-iTENG without bionic cilia unmodified TiO_2/PANI are not significantly changed in the light-dark-light state (see Fig.s3.6 a,b). Hence, it indicates that the increase of electron transfer is induced by photon excitation effect of the insulator surfaces can be eliminated.

After the surface covered with bionic cilia modified TiO_2/PANI, the open circuit voltage and the short circuit current of Pem-iTENG modified TiO_2/PANI are significantly improved in light compared with in dark. The open circuit voltage increases from 38.8 V in dark to 59.87 V in light, and the short circuit current increases from 2.4 mA in dark to 4.5 mA in light (see Fig.s3.6c,d). This indicates the enhancement of light effect experiments of Pem-iTENG is attributed to the photocatalysis. Therefore, compared with the improvement of the light effect experiments of Pem-iTENG with bionic cilia modified TiO_2/PANI in dark and light, we confirmed that the increase of electron transfer is induced by photocatalysis after excluding the light excitation.

Thirdly, through magnetic self-assembly technology, the solid-liquid contact angle of the PDMS film with bionic cilia is 165.28° (see Fig.s3.7c), while bionic cilia modified TiO_2/PANI is 162.04° (see Fig.s3.7d). It indicates that the PDMS films with bionic cilia modified or unmodified TiO_2/PANI have certain super-hydrophobic properties, and the modification of TiO_2/PANI has no significant effect on the solid-liquid contact angle. Moreover, PDMS film modified TiO_2/PANI with a light

irradiation result shows that the solid-liquid contact angle after the light illumination of 1h is 159.74°, and the solid-liquid contact angle after the light illumination of 3h is 162.13°. Comparing the contact angles of bionic cilia modified or unmodified TiO_2/PANI, it shows that the light irradiation time has no significant effect on the solid-liquid contact angle. Therefore, we can exclude the influence of the contact angle on the solid-liquid surfaces leading to the increase of electron transfer.

In summary, we have verified that a significant enhancement in the charge density of Pem-iTENG modified TiO_2/PANI is illuminated by sunlight during the fluctuation simulation test. Then, we have confirmed that the increase of electron transfer is induced by photocatalysis instead of photon excitation effect. Finally, the influence of the contact angle on the solid-liquid surfaces leading to the increase of electron transfer is excluded. Therefore, we can explain indirectly through systematic experiments that there are charge trsanfer/exciton dissociation at the interface between PANI and TiO_2. Moreover, we demonstrated that through systematic experiments, our hypothesis for the photoelectric-mechanical integrated triboelectric nano-generator is correct, and the working mechanism of the proposed Pem-iTENG is fully convincing.

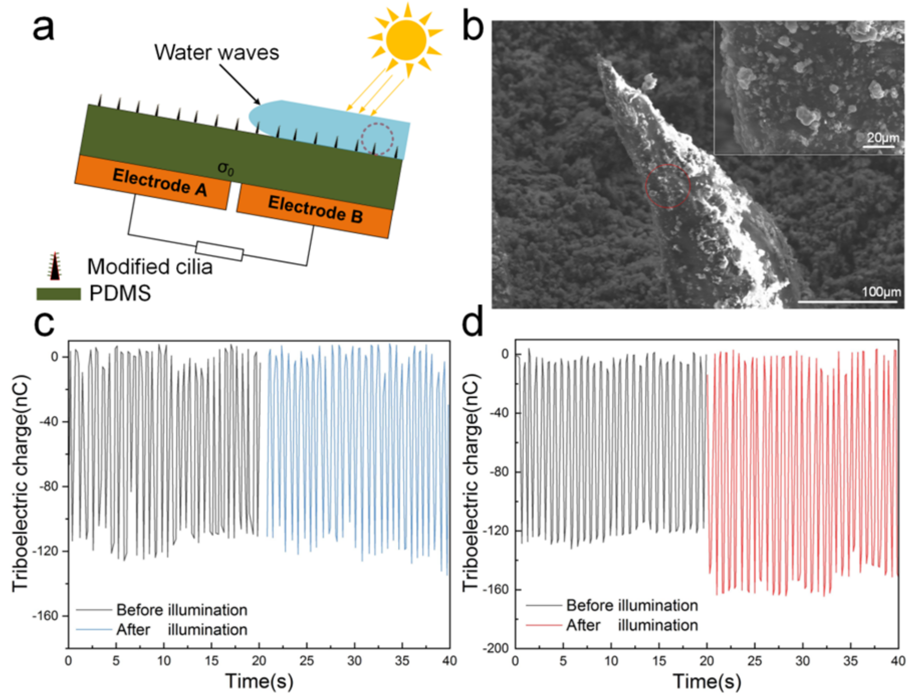

Fig.s3.5 Charge density diagram under different experimental conditions: (a) simple structure diagram of Pem-iTENG, (b) single bionic cilia modified TiO_2/PANI, (c) charge density unmodified TiO_2/PANI before and after the illumination, (d) charge density modified TiO_2/PANI before and after the illumination.

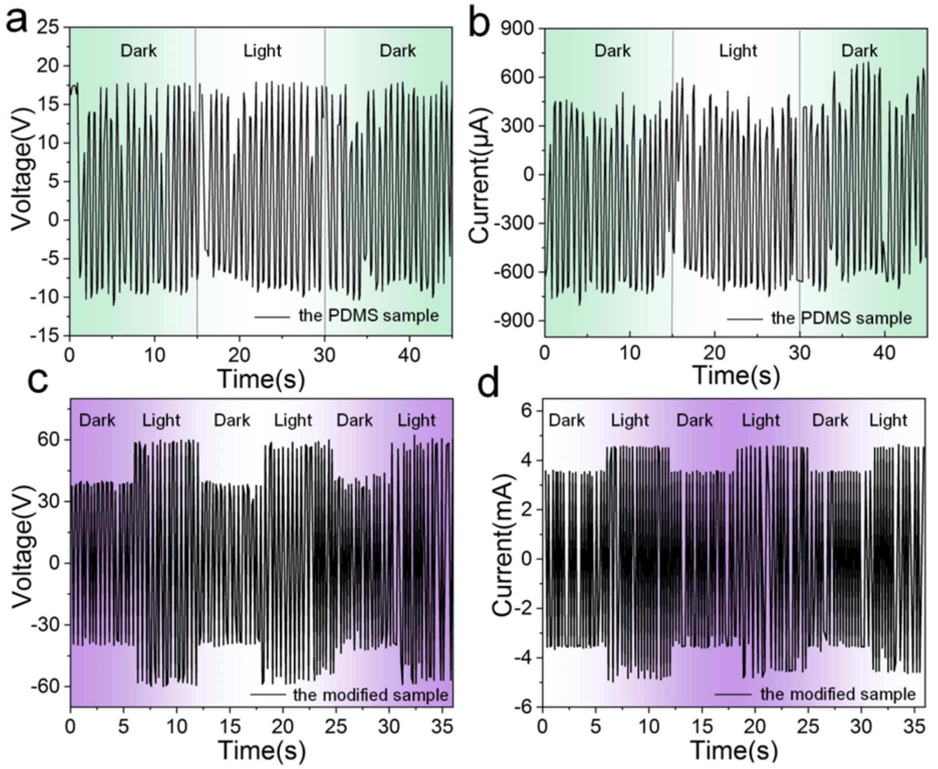

Fig.s3.6 the light effect experiments under light-darknes: (a) open circuit voltage and short circuit current (b) of PDMS without bionic cilia unmodified TiO_2/PANI, (c) open circuit voltage and short circuit current (d) of PDMS with bionic cilia modified TiO_2/PANI.

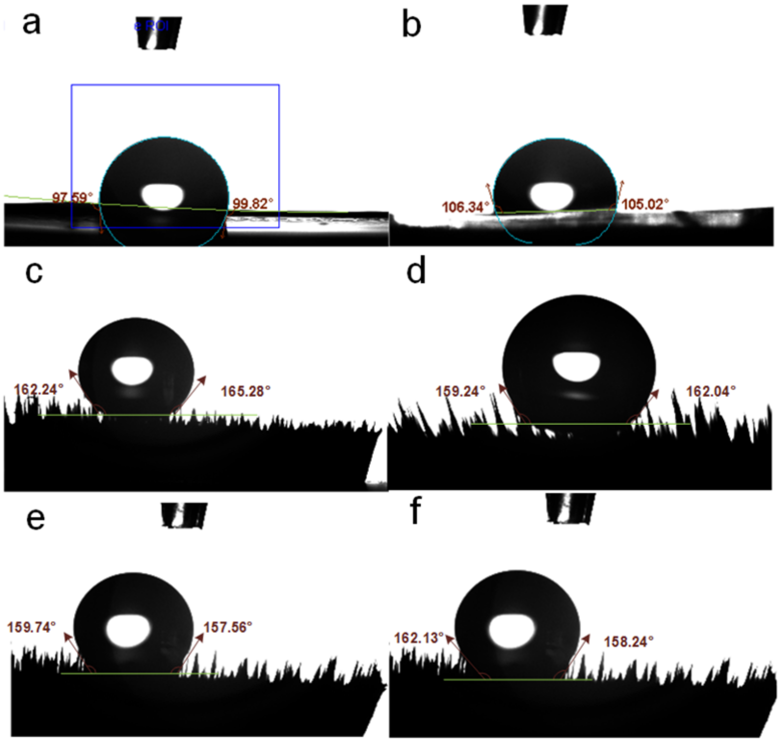

Fig. s3.7 Solid liquid contact angle of different samples: (a) the contact angle of pure PDMS, (b)

The contact angle of the PDMS doped with cobalt powder, (c) the contact angle of PDMS with bionic cilia unmodified TiO_2/PANI, (d) the contact angle of PDMS with bionic cilia modified TiO_2/PANI, (e) the contact angle of PDMS with bionic cilia modified TiO_2/PANI after illumination for 1 h, (f) the contact angle of PDMS with bionic cilia modified TiO_2/PANI after illumination for 3 h.

6. In the part of energy efficiency calculation, the data used to calculate output electrical energy are collected before rectification or after? Are there any difference between the calculated values by using the data collected before or after rectification? Please give the details. Additionally, what is the value of the "η"? And why use the "η" during input light energy calculation?

Response: Thanks to your comments. The data for calculating the output power is collected before rectification, and there are differences between the calculated results by using the data before rectification and after. According to the calculation method of the energy conversion rate of Pem-iTENG in Question 4, the energy conversion rate before and after rectification can be obtained. The calculated energy conversion rate before rectification is 67.42%, and the energy conversion rate after rectification is 62.76%. Considering the energy consumption of the rectifier and load, we used the data before rectification to calculate the energy conversion rate of the triboelectric nanogenerator. Although there is a certain deviation, the error is very small and can be ignored. The following table (Table s3.1) shows the calculation process of the energy conversion rate before and after the rectification of Pem-iTENG.

Table s3.1 Conversion rate calculation before and after rectification

Parameters / Samples	Output current (J_{sc}, μAcm^{-2})	Output energy (E_{out}, mJ)	Mechanical energy (E_{me}, mJ)	luminous energy (E_{op}, mJ)	Energy conversion rate(ECR)
**Pyramid-TENG**	24.5	1.038	40	0	3.27%
**PTFE-TENG**	106.1	13.564	40	0	33.91%
**3D-TENG**	160.1	21.532	40	0	53.83%
**Pem-iTENG-Before**	221.6	32.563	40.799	7.5	67.42%
**Pem-iTENG-After**	208.2	30.310	40.799	7.5	62.76%

Note: The power of the xenon lamp is 300w, the value of η is 1.5%, and the input mechanical energy of the other three TEMGs is 40 J by default.

η is the utilization rate of light energy, that is, the electric energy consumed by solar energy/xenon lamp received by Pem-iCM with a value of 1.5% (J. Mater. Chem. Cmem 4, 2016, 10395). Light energy is the driving force of photocatalysis for Pem-iCM. The utilization rate of light energy generally refers to the ratio between the

energy contained in organic matter produced by crops during photosynthesis and the solar energy received by this land per unit land area. The theoretical calculation value can generally reach 6.0% ~ 8.0%, but only 0.5% ~ 1.0% in actual production, and up to 2% at most. Because Pem-iTENG is inspired by the photocatalytic principle of green plants, only part of the frequency of solar energy irradiated on Pem-iCM can be absorbed and utilized by Pem-iTENG. At the same time, the solar energy irradiated on the surface of the friction composite film is not completely absorbed, part of it is lost into the air due to reflection, and part of it is not absorbed because it passes through the friction composite film. Most of solar energy absorbed by the friction composite film is converted into heat energy, and only a small part of the energy is utilized by the photocatalysis. Therefore, the input light energy cannot be simply considered as the power consumption of xenon lamp. Here, we define a conversion parameter. That is, the utilization rate of light energy, which is not the same concept as the photoelectric conversion efficiency in the photovoltaic cells, and has nothing to do with the conversion efficiency (η) from light energy to electric energy of solar cells (see Figure s3.8 for details).

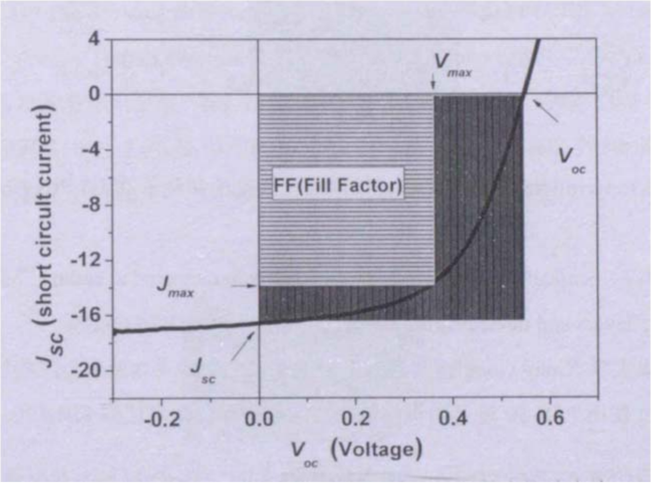

Fis. s3.8 J-V curve of ideal solar cell under illumination conditions. The performance parameters of solar cells mainly include open circuit voltage (V_{OC}), short circuit current (J_{SC}), fill factor (*FF*), power conversion efficiency (*PCE*) and external quantum efficiency (*EQE*). When the voltage is open-circuit, the measured voltage drop between both ends is the open-circuit voltage of solar cell. The conversion efficiency of the solar cell is calculated by the following formula of $\eta=P_{max}/P_{in}=V_{oc}\times I_{sc}\times FF/P_{in}$. Whereby, P_{in} is the power of the incident light. In order to obtain high conversion efficiency, the battery must have high open circuit voltage, large short circuit current, high fill factor and low dark current.
